# A Pyridyl-1,2-azaborine Ligand for Phosphorescent Neutral Iridium(III) Complexes

**DOI:** 10.1021/acs.inorgchem.2c04449

**Published:** 2023-01-25

**Authors:** Andrea Baschieri, Flavia Aleotti, Elia Matteucci, Letizia Sambri, Michele Mancinelli, Andrea Mazzanti, Enrico Leoni, Nicola Armaroli, Filippo Monti

**Affiliations:** † Istituto per la Sintesi Organica e la Fotoreattività, Consiglio Nazionale delle Ricerche, Via P. Gobetti 101, 40129 Bologna, Italy; ‡ Dipartimento di Chimica Industriale “Toso Montanari”, Università di Bologna, Viale Risorgimento 4, 40136 Bologna, Italy; § Laboratorio Tecnologie dei Materiali Faenza, ENEA, Via Ravegnana 186, 48018 Faenza, RA, Italy

## Abstract

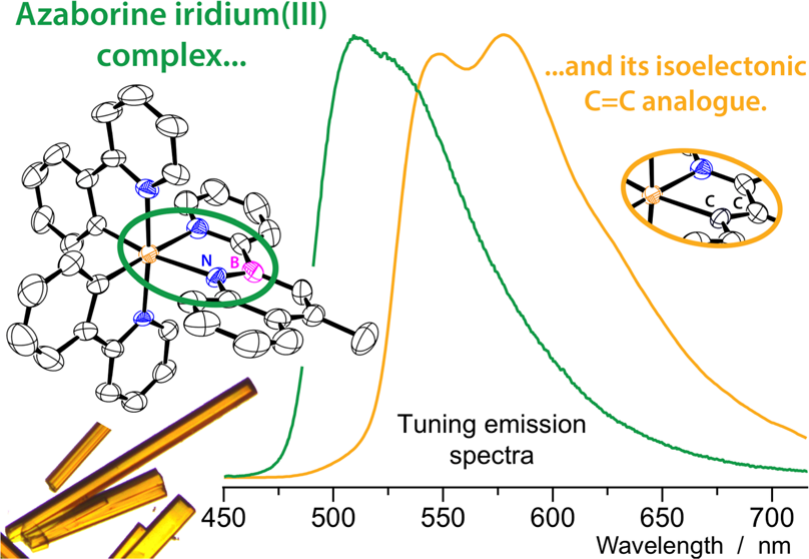

A novel 1,2-azaborine (*i.e.*, 4-methyl-2-(pyridin-2-yl)-2,1-borazaronaphthalene, **1a**) has been synthesized and used for the first time as a B–N alternative to common cyclometalating ligands to obtain neutral phosphorescent iridium(III) complexes (*i.e.*, **2a**, **3**, and **4**) of general formula [Ir(C^∧^N)_2_(N^∧^NB)], where C^∧^N indicates three different cyclometalating ligands (Hppy = 2-phenylpyridine; Hdfppy = 2-(2,4-difluoro-phenyl)pyridine; Hpqu = 2-methyl-3-phenylquinoxaline). Moreover, the azaborine-based complex **2a** was compared to the isoelectronic C=C iridium(III) complex **2b**, obtained using the corresponding 2-(naphthalen-2-yl)pyridine ligand **1b**. Due to the dual cyclometalation mode of such C=C ligand, the isomeric complex **2c** was also obtained. All new compounds have been fully characterized by NMR spectroscopy and high-resolution mass spectrometry (MS), and the X-ray structure of **2a** was determined. The electronic properties of both ligands and complexes were investigated by electrochemical, density functional theory (DFT), and photophysical methods showing that, compared to the naphthalene analogues, the azaborine ligand induces a larger band gap in the corresponding complexes, resulting in increased redox gap (basically because of the highest occupied molecular orbital (HOMO) stabilization) and blue-shifted emission bands (*e.g.*, λ_max_ = 523 *vs* 577 nm for **2a***vs***2b**, in acetonitrile solution at 298 K). On the other hand, the ^3^LC nature of the emitting state is the same in all complexes and remains centered on the pyridyl-borazaronaphthalene or its C=C pyridyl-naphthalene analogue. As a consequence, the quantum yields of such azaborine-based complexes are comparable to those of the more classical C=C counterparts (*e.g.*, photoluminescence quantum yield (PLQY) = 16 *vs* 22% for **2a***vs***2b**, in acetonitrile solution at 298 K) but with enhanced excited-state energy. This proves that such type of azaborine ligands can be effectively used for the development of novel classes of photoactive transition-metal complexes for light-emitting devices or photocatalytic applications.

## Abstract 2

A pyridyl-azaborine ligand is used as a B−N alternative to a more common C=C cyclometalating ligand for the synthesis of neutral photoactive iridium(III) complexes. The replacement of the carbon−carbon bond with the isoelectronic boron−nitrogen equivalent leads to a blueshift in the emission spectra of the corresponding complexes, preserving their luminescence quantum yields and an increase in the radiative rate constants. A series of three azaborine-based iridium(III) complexes emitting in the whole visible spectrum have been synthesized and fully characterized.

## Introduction

The introduction of heteroatoms in carbon-based chemical structures is a popular strategy to expand chemical diversity. In particular, borazaro compounds (also called azaborines),^1^ have raised great interest in recent years for their potential applications in biomedical research^2^ and optoelectronics.^3−6^ The peculiarity of this class of compounds consists in the replacement of one aromatic sp^2^ C=C unit with an isoelectronic B–N one,^7^ leading to a wide range of possible organic structures with different physical and chemical properties.^8−11^

Both theoretical calculations and experimental data prove that the aromaticity and thermal stability of azaborines are only slightly lower than those of the corresponding C=C aromatic systems.^12^ Despite the same number of valence electrons and a similar stability, the polarization of the B–N bond in azaborines modifies their chemical reactivity and molecular properties, with respect to the corresponding “all-carbon” analogues. Indeed, this local dipole moment can significantly alter the character of the frontier molecular orbitals (FMOs) and the intermolecular interactions in the solid phase.^13,14^ In fact, the delocalized π orbitals of an aromatic system can interact with the nitrogen-donor and boron–acceptor orbitals, decreasing the highest occupied molecular orbital (HOMO)–lowest unoccupied molecular orbital (LUMO) gap, while preserving molecular planarity.^15^ Such characteristics make most azaborines luminescent molecules,^16,17^ with promising applications in organic light-emitting diodes (OLEDs).^18,19^ In general, iridium-based complexes are widely used in solid-state lighting devices,^20,21^ as probes for in vitro bioimaging,^22−25^ and in photoredox catalysis.^26,27^

At present, the leading emissive materials in electroluminescent devices (such as OLEDs and light-emitting electrochemical cells (LECs)) are transition-metal complexes (TMCs), due to their relatively facile synthesis and their emission from triplet excited states, which allows for greater electroluminescence efficiency compared to singlet emitters.^28−31^ Among all TMCs, cyclometalated iridium(III) complexes have proven to be the most versatile, mainly due to their tunable phosphorescent emission, covering the whole visible spectrum from blue to red, together with high photoluminescence quantum yields and good chemical stability.^32^ Actually, both neutral and ionic (anionic and cationic) luminescent iridium(III) complexes can be synthesized, using chelating ligands, which could be monoanionic, dianionic, and neutral. The neutral complexes are the most suitable for OLED applications since they allow the device preparation *via* vacuum sublimation.^33,34^

Herein, we focus our attention on a novel class of bidentate ligands for transition-metal complexes, based on functionalized 1,2-azaborines. In particular, we decided to functionalize the boron atom of a 2,1-borazaronaphthalene with a pyridyl moiety, to obtain a suitable chelating ligand for iridium(III) complexes.

Molander and co-workers reported an effective methodology for the direct synthesis of 2,1-borazaronaphthalenes containing different *N*-heterocyclic substituents bound to boron, *via* the reaction of substituted 2-aminostyrenes with organotrifluoroborates.^35^ However, no compounds carrying the pyridin-2-yl substituent were reported, probably due to the instability of the pyridyl trifluoroborate used in the proposed reaction method.^36^

**Chart 1:**
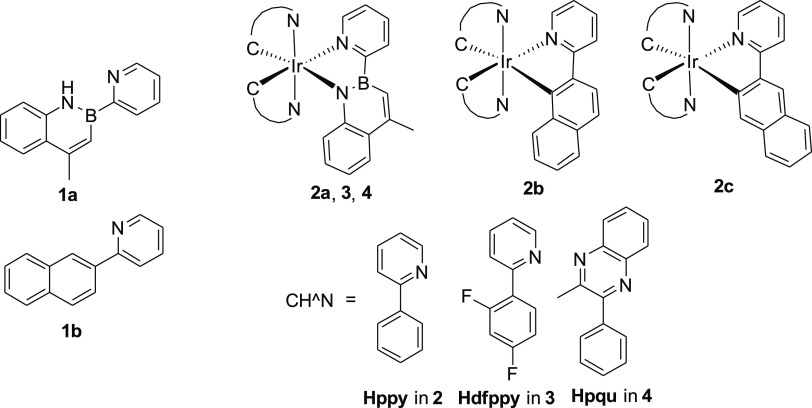
Molecular Structures of the Investigated Compounds

The functionalization of 2,1-borazaronaphthalenes with a pyridin-2-yl unit is of great interest since it could create new chelating ligands able to effectively coordinate a metal center through the two nitrogen atoms (*i.e.*, one from the pyridyl moiety and the other from the azaborine unit), forming the typical five-membered metallacycle, as shown in Chart 1. Since 1,2-azaborines are aromatic compounds, the deprotonation of the nitrogen of the borazaronaphthalene moiety and the resulting binding to the iridium(III) ion could be thought of as a cyclometallation reaction with the anionic azaborine ligand serving as a strong field chelator. This could significantly alter the relative energy of the frontier molecular orbitals of the octahedral complexes, providing novel properties to the corresponding luminescent materials (see below).

Based on the above considerations, we designed and synthesized a novel pyridyl-1,2-azaborine derivative (*i.e.*, 4-methyl-2-(pyridin-2-yl)-2,1-borazaronaphthalene **1a**, see Chart 1), which was subsequently used as a ligand to obtain neutral luminescent iridium(III) complexes (*i.e.*, **2a**, **3**, and **4**). To the best of our knowledge, these are the first examples of phosphorescent iridium(III) complexes containing an azaborine ligand.

In addition, to evaluate the effects of this new B–N ligand on the photophysical properties of the related complexes, we also synthesized and characterized the equivalent isoelectronic C=C ligand **1b** and its isosteric iridium(III) complex **2b** (Chart 1), along with its isomer **2c**, which is unavoidably formed during the synthesis.

## Experimental Section

### General Information

2-Isopropenylaniline (**5**), 2-bromopyridine (**7**), and 2-naphthylboronic acid (**9**) were commercially available. 2-(Naphthalen-2-yl)pyridine (**1b**) was prepared according to reported methods.^37^ Analytical-grade solvents and all of the commercially available reagents were used without any further purification, unless otherwise specified. Chromatographic purifications were performed using 230–400 mesh silica gel (pore size 60 Å) and activated basic Al_2_O_3_ (58 Å, 60 mesh powder). Tetrahydrofuran (THF) and toluene have been dried before use by distillation from Na/benzophenone. Reactions that needed anhydrous conditions were performed under dried nitrogen flow (inert atmosphere). The glassware used in these reactions was placed in an oven at +70 °C for at least 3 h immediately before use. ^1^H, ^19^F, ^11^B, and ^13^C NMR spectra were recorded on a Varian INOVA (600 MHz for ^1^H, 564.3 MHz for ^19^F, 192.4 MHz for ^11^B, 150.8 MHz for ^13^C) spectrometer. All spectra were acquired at +25 °C. Chemical shifts (δ) are reported in ppm relative to residual solvent signals for ^1^H and ^13^C NMR (^1^H NMR: 7.26 ppm for CDCl_3_, 5.33 ppm for CD_2_Cl_2_; ^13^C NMR: 77.0 ppm for CDCl_3_, 53.84 ppm for CD_2_Cl_2_) or relative to internal standard as chemical shift reference for ^19^F and ^11^B NMR (^19^F NMR: −163 ppm for C_6_F_6_; ^11^B NMR: 0 ppm for BF_3_·Et_2_O). ^13^C{^1^H} NMR spectra were acquired with ^1^H broad band decoupled mode. Coupling constants are given in hertz. The abbreviations used to indicate the multiplicity of signals are: s, singlet; d, doublet; t, triplet; dd, double doublet; ddd, double double doublet; dt, double triplet; m, multiplet. The high-resolution mass spectra (HRMS) were obtained with an electrospray ionization-quadrupole time-of-flight (ESI-QTOF) (Agilent Technologies, model G6520A) instrument, and the *m*/*z* values are referred to the monoisotopic mass.

#### Synthesis of Ligand 4-Methyl-2-(pyridin-2-yl)-1,2-dihydrobenzo[*e*][1,2]azaborinine (**1a**)

2-Isopropenylaniline **5** (476 μL, 3.5 mmol) was dissolved in dry toluene (50 mL) at 0 °C under nitrogen atmosphere; then, BCl_3_ and 1 M hexane solution was added dropwise (4.5 mL, 4.6 mmol). The yellowish solid formed dissolved to give a pale-yellow solution when the mixture is refluxed. The mixture was stirred for 1 h and then was cooled to room temperature and the excess BCl_3_ was removed under vacuum together with the solvent. The crude was dissolved in anhydrous THF, and then the solvent was evaporated again under vacuum to remove *via* stripping the BCl_3_ traces. Product **6** was used in the next step without further purification. In a round-bottom flask, 2-bromopyridine **7** (500 μL, 5.25 mmol) was dissolved in dry THF under nitrogen atmosphere. The solution was then cooled down to −78 °C, and *n*-butyl lithium and 1.6 M hexane solution (5.9 mL) was added dropwise to get an orange solution containing 2-lithiumpyridine **8** that was used for the azaborine synthesis, without any additional purification. Product **6** was dissolved in 2 mL of anhydrous THF and was added dropwise to the solution of 2-lithiumpyridine **8** at −78 °C; then, the mixture was left to reach room temperature and stirred overnight. The solvent was then removed in vacuum, and the resulting crude was purified on silica gel (hexane/ethyl acetate 9:1). Product **1a** was obtained as a yellow-brown solid (500 mg, 65% yield). ^1^H NMR (600 MHz, CDCl_3_) δ 9.40 (bs, NH), 8.82 (dt, *J*_D_ = 4.8 Hz, *J*_T_ = 1.3 Hz, 1H), 8.07 (d, *J* = 7.6 Hz, 1H), 7.87 (d, *J* = 8.1 Hz, 1H), 7.78 (t, *J* = 8.1 Hz, 1H), 7.51–7.45 (m, 2H), 7.35–7.31 (m, 1H), 7.24 (ddd, *J* = 8.0, 6.5, 1.7 Hz, 1H), 7.16 (bs, 1H), 2.70 (d, *J* = 0.9 Hz, 3H); ^13^C{^1^H} NMR (150.8 MHz, CDCl_3_) δ 161.1 (B–C, br), 153.0 (C), 149.1 (CH), 140.4 (C), 135.7 (CH), 129.6 (CH), 128.4 (CH), 126.9 (B–CH, br), 125.9 (C), 125.6 (CH), 123.9 (CH), 121.0 (CH), 119.3 (CH), 23.2 (CH_3_); ^11^B NMR (192.4 MHz, CDCl_3_) δ 26.94. HRMS (ESI-QTOF) *m*/*z*: for C_14_H_13_BN_2_: [(M + H)^+^] calcd 220.1281; found 220.1310.

### General Procedure for the Synthesis of Complexes **2a**, **3**, and **4**

The desired iridium(III) dimer **10**, **11**, or **12** (0.03 mmol) was dissolved in a 3:1 mixture of CH_2_Cl_2_/EtOH (6 mL); then, AgPF_6_ (3 equiv) was added in the absence of light. The mixture was kept in the dark and stirred at 60 °C overnight. The solution was evaporated to dryness, and the resulting solid was used in the next step of the synthesis without further purification. A solution of compound **1a** (2.5 equiv, 16.5 mg, 0.075 mmol) and NaH 60% dispersion in mineral oil (6 equiv, 7.2 mg, 0.18 mmol) in anhydrous CH_2_Cl_2_ (5 mL) was stirred for 1 h at room temperature and then added dropwise to the previous solid, and the mixture was stirred for further 24 h at room temperature. After this time, the solvent was evaporated and the crude was purified by flash chromatography.

#### Synthesis of Complex [Ir(ppy)_2_(azab-py)] (**2a**)

Purified by flash chromatography on Al_2_O_3_ using a mixture of hexane/ethyl acetate 95:5. The complex was crystallized by slow diffusion of Et_2_O vapor in a solution of CH_2_Cl_2_ (yield = 61%, 26.2 mg). ^1^H NMR (CDCl_3_, 300 MHz) δ 8.32 (ddd, *J* = 5.8, 1.5, 0.6 Hz, 1H), 8.18 (ddd, *J* = 7.6, 1.6, 0.9 Hz, 1H), 7.81 (d, *J* = 7.8 Hz, 1H), 7.73–7.68 (m, 3H), 7.65–7.44 (m, 7H), 7.10 (d, *J* = 0.8 Hz, 1H), 6.96–6.68 (m, 9H), 6.436.40 (m, 1H), 6.21 (dd, *J* = 7.6, 1.0 Hz, 1H), 2.71 (d, *J* = 0.8 Hz, 3H); ^1^H NMR (CD_2_Cl_2_, 600 MHz) δ 8.32 (dd, *J* = 5.8, 0.9 Hz, 1H), 8.20 (d, *J* = 7.6 Hz, 1H), 7.89 (d, *J* = 8.2 Hz, 1H), 7.79 (d, *J* = 8.2 Hz, 1H), 7.73–7.66 (m, 4H), 7.66–7.61 (m, 2H), 7.58–7.52 (m, 2H), 7.51 (dd, *J* = 8.7, 0.9 Hz, 1H), 7. 12 (bs, 1H), 7.01–6.90 (m, 3H), 6.89–6.77 (m, 5H), 6.64 (ddd, *J* = 8.6, 6.7, 1.5 Hz, 1H), 6.44 (dd, *J* = 7.6, 0.7 Hz, 1H), 6.23 (dd, *J* = 7.4, 0.7 Hz, 1H), 2.68 (bs, 3H); ^13^C{^1^H} NMR (CD_2_Cl_2_, 150.8 MHz) δ 177.2 (C–B), 168.8 (C), 157.2 (C), 156.6 (C), 152.6 (C), 151.7 (CH), 150.3 (CH), 149.5(C), 148.3 (CH), 144.7 (C), 143.5 (C), 136.7 (CH), 136.2 (CH), 135.6 (CH), 132.2 (CH), 131.8 (CH), 130.54 (CH), 130.5 (CH), 129.9 (CH), 129.5 (C), 129.4 (CH–B), 128.6 (C), 127.6 (CH), 126.2 (CH), 125.4 (CH), 124.7 (CH), 124.6 (CH), 124.3 (CH), 122.7 (CH), 121.9 (CH), 121.6 (CH), 120.4 (CH), 119.1 (CH), 118.8 (CH), 118.3 (CH), 23.4 (CH_3_); ^11^B NMR (CD_2_Cl_2_, 192.4 MHz) δ 33.68. HRMS (ESI-QTOF) *m*/*z*: for C_36_H_28_BIrN_4_: [(M + H)^+^] calcd 718.2122; found 718.2144.

#### Synthesis of Complex [Ir(dfppy)_2_(azab-py)] (**3**)

Purified by flash chromatography on Al_2_O_3_ using a mixture of hexane/ethyl acetate 95:5. The complex was precipitated using the double-layer diffusion method with hexane in a CH_2_Cl_2_ solution (yield = 49%, 23.2 mg). ^1^H NMR (CD_2_Cl_2_, 600 MHz) δ 8.33 (dd, *J* = 5.9, 0.8 Hz, 1H), 8.27 (d, *J* = 8.6 Hz, 1H), 8.23 (d, *J* = 7.6 Hz, 1H), 8.18 (d, *J* = 8.4 Hz, 1H), 7.76–7.67 (m, 4H), 7.61 (t, *J* = 7.6 Hz, 1H), 7.53 (d, *J* = 6.0 Hz, 1H), 7.33 (dd, *J* = 8.7, 0.8 Hz, 1H), 7.11 (bs, 1H), 7.03 (ddd, *J* = 7.4, 5.5, 1.4 Hz, 1H), 6.92–6.86 (m, 2H), 6.84 (ddd, *J* = 7.4, 5.9, 1.3 Hz, 1H), 6.77 (ddd, *J* = 8.7, 6.8, 1.6 Hz, 1H), 6.51 (ddd, *J* = 12.6, 9.4, 2.4 Hz, 1H), 6.46 (ddd, *J* = 12.7, 9.2, 2.4 Hz, 1H), 5.87 (dd, *J* = 9.2, 2.2 Hz, 1H), 5.71 (dd, *J* = 8.5, 2.0 Hz, 1H), 2.68 (bs, 3H); ^19^F NMR (CD_2_Cl_2_, 564.3 MHz) δ −108.2 (q, *J*_CF_ = 9.1 Hz, 1F), −109.3 (q, *J*_CF_ = 9.5 Hz, 1F), −110.3 (t, *J*_CF_ = 11.5 Hz, 1F), −110.9 (t, *J*_CF_ = 11.4 Hz, 1F); ^13^C{^1^H} NMR (CD_2_Cl_2_, 150.8 MHz) δ 176.3 (C–B), 165.34 (C, d, *J*_CF_ = 7.4 Hz), 165.24 (C, d, *J*_CF_ = 7.3 Hz), 164.2 (C, dd, *J*_CF_ = 256.3, 11.7 Hz), 163.8 (C, dd, *J*_CF_ = 255.1, 12.7 Hz), 162.0 (C, dd, *J*_CF_ = 258.7, 12.6 Hz), 161.8 (C, dd, *J*_CF_ = 260.5, 12.6 Hz), 161.8 (C, d, *J*_CF_ = 6.0 Hz), 160.9 (C, d, *J*_CF_ = 6.6 Hz), 152.1 (C), 151.8 (CH), 150.1 (CH), 150.0 (C), 148.5 (CH), 137.8 (CH), 137.3 (CH), 136.2 (CH), 130.8 (CH), 129.5 (C), 128.5 (C, t, *J*_CF_ = 3.7 Hz), 127.7 (C, t, *J*_CF_ = 3.5 Hz), 127.0 (CH), 126.9 (CH–B), 126.5 (CH), 125.7 (CH), 124.7 (CH), 123.2 (CH, d, *J*_CF_ = 19.5), 123.0 (CH), 122.9 (CH, d, *J*_CF_ = 19.5), 122.2 (CH), 118.7 (CH), 113.7 (CH, dd, *J*_CF_ = 16.4, 2.5 Hz), 113.6 (CH, dd, *J*_CF_ = 16.3, 3.1 Hz), 98.0 (CH, t, *J*_CF_ = 27.0 Hz), 96.7 (CH, t, *J*_CF_ = 27.0 Hz), 23.3 (CH_3_); ^11^B NMR (CD_2_Cl_2_, 192.4 MHz) δ 33.76. HRMS (ESI-QTOF) *m*/*z*: for C_36_H_24_BF_4_IrN_4_: [(M + H)^+^] calcd 790.1745; found 790.1740.

#### Synthesis of Complex [Ir(pqu)_2_(azab-py)] (**4**)

Purified by flash chromatography on Al_2_O_3_ using a mixture of hexane/ethyl acetate 7:3. The complex was precipitated using the double-layer diffusion method with hexane in a CH_2_Cl_2_ solution (yield = 38%, 19.5 mg). ^1^H NMR (CDCl_3_, 300 MHz) δ 9.32 (dd, *J* = 9.0, 1.0 Hz, 1H), 8.36 (dd, *J* = 8.2, 0.9 Hz, 1H), 7.89 (dd, *J* = 8.2, 1.4 Hz, 1H), 7.82 (dd, *J* = 8.2, 0.8 Hz, 1H), 7.75–7.71 (m, 1H), 7.69 (ddd, *J* = 4.4, 1.4, 0.8 Hz, 1H), 7.66–7.63 (m, 1H), 7.50 (td, *J* = 7.6, 1.5 Hz, 1H), 7.41 (ddd, *J* = 8.2, 6.9, 1.2 Hz, 1H), 7.35 (ddd, *J* = 8.2, 6.9, 1.3 Hz, 1H), 7.10 (ddd, *J* = 8.3, 7.1, 1.4 Hz, 1H), 7.05–7.02 (m, 1H), 6.98–6.79 (m, 5H), 6.76–6.69 (m, 4H), 6.66–6.57 (m, 2H), 6.40 (dd, *J* = 7.7, 0.9 Hz, 1H), 6.36 (d, *J* = 0.9 Hz), 3.36 (s, 3H), 2.62 (s, 3H), 2.49 (d, *J* = 0.9 Hz, 3H); ^1^H NMR (CD_2_Cl_2_, 600 MHz) δ 9.19 (dd, *J* = 8.8, 0.8 Hz, 1H), 8.40 (d, *J* = 8.2 Hz, 1H), 7.92 (d, *J* = 8.1 Hz, 1H), 7.89 (dd, *J* = 8.3, 1.3 Hz, 1H), 7.76–7.72 (m, 2H), 7.68 (d, *J* = 5.5 Hz, 1H), 7.65 (dd, *J* = 8.3, 1.3 Hz, 1H), 7.56 (td, *J*_T_ = 7.6 Hz, *J*_D_ = 1.5 Hz, 1H), 7.44 (ddd, *J* = 8.3, 7.0, 1.3 Hz, 1H), 7.38 (ddd, *J* = 8.1, 7.0, 1.3 Hz, 1H), 7.14 (ddd, *J* = 8.2, 6.9, 1.1 Hz, 1H), 7.09 (d, *J* = 8.8 Hz, 1H), 7.00 (ddd, *J* = 8.1, 6.9, 1.1 Hz, 1H), 6.95 (ddd, *J* = 7.4, 5.6, 1.4 Hz, 1H), 6.85–6.81 (m, 2H), 6.81–6.73 (m, 4H), 6.62–6.57 (m, 2H), 6.45 (dd, *J* = 7.8, 0.9 Hz, 1H), 6.43 (bs, 1H), 3.33 (s, 3H), 2.68 (s, 3H), 2.48 (s, 3H); ^13^C{^1^H} NMR (CD_2_Cl_2_, 150.8 MHz) δ 166.0 (C), 165.6 (C), 160.1 (C), 157.8 (C), 152.2 (C), 152.1 (C), 149.8 (C), 149.2 (C), 148.4 (CH), 146.6 (C), 145.7 (C), 142.3 (C), 141.0 (C), 140.6 (C), 140.4 (C), 135.8 (CH), 134.3 (CH), 132.6 (CH), 130.8 (CH), 130.4 (CH), 130.3 (C), 130.0 (CH), 129.9 (CH), 129.8 (CH), 129.7_4_ (CH), 129.7 (CH), 129.4 (CH), 128.6 (CH), 128.5 (CH), 128.4 (CH), 128.1 127.8 (CH), 125.7 (CH), 125.5 (CH), 125.2 (CH), 124.0 (CH), 121.8 (CH), 121.0 (CH), 118.2 (CH), 28.3 (CH_3_), 26.8 (CH_3_), 23.0 (CH_3_). The signals of two carbon atoms bonded to boron were not observed owing to the strong broadening caused by the boron quadrupole. ^11^B NMR (CD_2_Cl_2_, 192.4 MHz) δ 33.51. HRMS (ESI-QTOF) *m*/*z*: for C_44_H_34_BIrN_6_: [(M + H)^+^] calcd 848.2653; found 848.2715.

### General Procedure for the Synthesis of Complexes **2b** and **2c**

Iridium(III) dimer **10** (150 mg, 0.14 mmol) was dissolved in 2-ethoxyethanol (2 mL); then, ligand **1b** (115 mg, 4 equiv) and K_2_CO_3_ (155 mg, 8 equiv) were added. The mixture was degassed with three vacuum–nitrogen cycles and stirred under a nitrogen atmosphere in the dark at 90 °C for 36 h. The solution was evaporated to dryness, and the resulting solid was purified by flash chromatography on silica deactivated with triethylamine using a mixture of petroleum ether/ethylic ether/dichloromethane 4.5:0.5:0.3.

#### Synthesis of Complex [Ir(ppy)_2_(1naft-py)] (**2b**)

*R_f_* = 0.15, yield = 32% (63.2 mg). ^1^H NMR (CD_2_Cl_2_, 600 MHz) δ 8.09 (d, *J* = 8.5 Hz, 1H), 8.05 (ddd, *J* = 5.8, 1.6, 0.6 Hz, 1H), 7.96 (d, *J* = 8.7 Hz, 1H), 7.89–7.79 (m, 3H), 7.78–7.66 (m, 4H), 7.65–7.46 (m, 5H), 7.20 (ddd, *J* = 8.0, 6.7, 1.2 Hz, 1H), 7.01 (td, *J*_T_ = 7.3 Hz, *J*_D_ = 1.3 Hz, 1H), 6.96–6.90 (m, 2H), 6.89–6.83 (m, 2H), 6.75 (ddd, *J* = 8.4, 6.8, 1.5 Hz, 1H), 6.70 (ddd, *J* = 7.3, 6.3, 1.5 Hz, 1H), 6.65 (ddd, *J* = 7.4, 5.9, 1.4 Hz, 1H), 6.50 (dd, *J* = 7.7, 1.2 Hz, 1H), 6.34 (dd, *J* = 7.2, 1.1 Hz, 1H); ^13^C{^1^H} NMR (CD_2_Cl_2_, 150.8 MHz) δ 185.7 (C), 172.8 (C), 170.3 (C), 169.8 (C), 168.1 (C), 160.4 (C), 154.9 (CH), 151.4 (CH), 148.2 (CH), 145.0 (C), 143.1 (C), 142.9 (C),142.5 (C), 137.3 (CH), 136.6 (CH), 136.2 (CH), 134.9 (C), 134.6 (CH) 131.9 (CH), 131.4 (CH), 130.6 (CH), 129.7 (CH), 128.0 (CH), 126.4 (CH), 124.7 (2 CH), 124.1 (CH), 123.6 (CH), 122.9 (CH), 122.8 (CH), 121.9 (CH), 121.7 (CH), 121.4 (CH), 119.9 (CH), 119.4 (CH), 119.2 (CH), 118.8 (CH). HRMS (ESI-QTOF) *m*/*z*: for C_37_H_26_IrN_3_: [M^+^] calcd 703.1727; found 703.1725.

#### Synthesis of Complex [Ir(ppy)_2_(3naft-py)] (**2c**)

*R_f_* = 0.13, yield = 43% (84.9 mg). ^1^H NMR (CD_2_Cl_2_, 600 MHz) δ 8.31 (s, 1H), 8.22 (d, *J* = 8.1 Hz, 1H), 8.18 (dd, *J* = 5.9, 0.6 Hz, 1H), 8.03 (dd, *J* = 5.6, 0.7 Hz, 1H), 7.86 (d, *J* = 8.4 Hz, 2H), 7.78–7.71 (m, 4H), 7.68 (dd, *J* = 5.9, 0.6 Hz, 1H), 7.57 (ddd, *J* = 8.1, 7.4, 1.5 Hz, 1H), 7.51 (ddd, *J* = 8.3, 7.5, 1.5 Hz, 1H), 7.37 (d, *J* = 8.1 Hz, 1H), 7.28–7.21 (m, 2H), 7.21 (s, 1H), 7.03–6.94 (m, 4H), 6.88 (td, *J*_T_ = 7.5 Hz, *J*_D_ = 1.4 Hz, 1H), 6.75 (ddd, *J* = 7.3, 6.0, 1.4 Hz, 1H), 6.70 (ddd, *J* = 7.3, 6.0, 1.4 Hz, 1H), 6.64 (dd, *J* = 7.0, 0.8 Hz, 1H), 6.48 (dd, *J* = 7.6, 0.8 Hz, 1H); ^13^C{^1^H} NMR (CD_2_Cl_2_, 150.8 MHz) δ 175.4 (C), 171.7 (C), 170.8 (C), 168.2 (C), 168.0 (C), 159.6 (C), 153.3 (CH), 151.8 (CH), 148.1 (CH), 146.4 (C),145.4 (C), 142.9 (C), 137.1 (CH), 136.2 (1C, 1CH), 135.6 (CH), 134.7 (CH) 133.2 (CH), 130.9 (CH), 130.6 (C), 130.3 (CH), 129.9 (CH), 128.9 (CH), 126.4_1_ (CH), 126.3_5_ (CH), 124.6 (CH), 124.4 (CH), 123.6 (2 CH), 123.2 (CH), 122.5 (CH), 121.7 (CH), 121.4 (CH), 120.5 (CH), 119.3 (CH), 119.2 (CH), 119.0 (CH). HRMS (ESI-QTOF) *m*/*z*: for C_37_H_26_IrN_3_: [M^+^] calcd 703.1727; found 703.1722.

### Electrochemistry

Voltammetric experiments were performed using a Metrohm AutoLab PGSTAT 302N electrochemical workstation in combination with the NOVA 2.0 software package. All of the measurements were carried out at room temperature in acetonitrile solutions with a sample concentration of ≈0.5 mM and using 0.1 M tetrabutylammonium hexafluorophosphate (electrochemical grade, TBAPF_6_) as the supporting electrolyte. Oxygen was removed from the solutions by bubbling nitrogen. All of the experiments were carried out using a three-electrode setup (BioLogic VC-4 cell, volume range: 1–3 mL) using a glassy carbon working electrode (having an active surface disk of 1.6 mm in diameter), the Ag/AgNO_3_ redox couple (0.01 M in acetonitrile, with 0.1 M TBAClO_4_ supporting electrolyte) as the reference electrode, and a platinum wire as the counter electrode. At the end of each measurement, ferrocene was added as the internal reference. Osteryoung square-wave voltammograms (OSWV) were recorded with a scan rate of 125 mV s^–1^, an SW amplitude of ±20 mV, and a frequency of 25 Hz. Cyclic voltammograms (CV) were recorded at 100 mV s^–1^.

### Computational Details

Density functional theory (DFT) calculations were carried out using the B.01 revision of the Gaussian 16 program package^38^ in combination with the M06 global-hybrid meta-generalized gradient approximation (meta-GGA) exchange–correlation functional.^39,40^ The fully relativistic Stuttgart/Cologne energy-consistent pseudopotential with multielectron fit was used to replace the first 60 inner-core electrons of the iridium metal center (*i.e.*, ECP60MDF), and it was combined with the associated triple-ζ basis set (*i.e.*, cc-pVTZ-PP basis).^41^ On the other hand, the Pople 6-31G(d,p) basis was adopted for all other atoms.^42,43^ All of the compounds were fully optimized without symmetry constraints, both in the ground state (S_0_) and in their lowest triplet states (T*_n_*), using the polarizable continuum model (PCM) to simulate acetonitrile solvation effects.^44−46^ Frequency calculations were always used to confirm that every stationary point found by geometry optimizations was actually a minimum on the corresponding potential-energy surface (no imaginary frequencies). The structural overlap between the X-ray crystal structure of complex **2a** and its ground-state computed one was obtained using the Visual Molecular Dynamics (VMD) program^47^ by minimizing the root-mean-square deviation (RMSD) of all of the atomic positions, except hydrogen atoms. Time-dependent DFT (TD-DFT) calculations,^48,49^ carried out at the same level of theory used for geometry optimizations, were employed to map the excited-state scenario of the investigated molecules in their optimized S_0_ geometry. For ligands **1a** and **1b**, the lowest six singlet excited states were also computed at the STEOM-DLPNO-CCSD level of theory,^50,51^ as implemented in ORCA 5.0.3,^52^ using the def2-TZVP basis set^53^ for all atoms and perturbative CPCM to simulate acetonitrile solvation. Multiwfn 3.8—a Multifunctional Wavefunction Analyzer was used to process ORCA outputs.^54^ Natural transition orbital (NTO) transformations were adopted to obtain a clear and compact orbital representation for the electronic transition density matrix in the case of complex multiconfigurational excitations.^55^ To investigate the nature of the triplet states, geometry optimizations and frequency calculations were performed at the spin-unrestricted UM06 level of theory, imposing a spin multiplicity of 3; the ground-state minimum-energy geometry was used as initial guess for T_1_, while TD-DFT optimized geometries were taken as input for the UDFT optimization of upper-lying triplets. All of the pictures showing molecular orbitals and spin-density surfaces were created using GaussView 6.^56^

### Photophysical Measurements

The spectroscopic investigations were carried out in spectrofluorimetric-grade solvents (*i.e.*, dichloromethane and acetonitrile). The absorption spectra were recorded with PerkinElmer Lambda 950 spectrophotometer. For the photoluminescence experiments, the sample solutions were placed in fluorimetric Suprasil quartz cuvettes (10.00 mm) and dissolved oxygen was removed by bubbling argon for 30 min. The uncorrected emission spectra were obtained with an Edinburgh Instruments FLS920 spectrometer equipped with a Peltier-cooled Hamamatsu R928 photomultiplier tube (spectral window: 185–850 nm). An Osram XBO xenon arc lamp (450 W) was used as the excitation light source. The corrected spectra were acquired by means of a calibration curve, obtained using an Ocean Optics deuterium–halogen calibrated lamp (DH-3plus-CAL-EXT). The photoluminescence quantum yields (PLQYs) in solution were obtained from the corrected spectra on a wavelength scale (nm) and measured according to the approach described by Demas and Crosby,^57^ using an air-equilibrated water solution of tris-(2,2′-bipyridyl)ruthenium(II) dichloride (PLQY = 0.028)^58^ as reference for the complexes and/or an air-equilibrated water solution of quinine sulfate in 1 N H_2_SO_4_ (PLQY = 0.546)^59^ as reference for the ligands. The emission lifetimes (τ) were measured through the time-correlated single photon counting (TCSPC) technique using a HORIBA Jobin Yvon IBH FluoroHub controlling a spectrometer equipped with a pulsed NanoLED (λ_exc_ = 280 and 370 nm) or pulsed SpectraLED (λ_exc_ = 370 nm) as the excitation source and a red-sensitive Hamamatsu R-3237-01 PMT (185–850 nm) as the detector. The analysis of the luminescence decay profiles was accomplished with the DAS6 Decay Analysis Software provided by the manufacturer, and the quality of the fit was assessed with the χ^2^ value close to unity and with the residuals regularly distributed along the time axis. To record the 77 K luminescence spectra, samples were put in quartz tubes (2 mm inner diameter) and inserted into a special quartz Dewar flask filled with liquid nitrogen. Poly(methyl methacrylate) (PMMA) films containing 1% (w/w) of the complex were obtained by drop-casting, and the thickness of the films was not controlled. Solid-state PLQY values were calculated by corrected emission spectra obtained from an Edinburgh FLS920 spectrometer equipped with a barium sulfate-coated integrating sphere (diameter of 4 in) following the procedure described by Würth et al.^60^ Experimental uncertainties are estimated to be ±8% for τ determinations, ±10% for PLQYs, and ±2 and ±5 nm for absorption and emission peaks, respectively.

## Results and Discussion

### Synthesis

**Scheme 1:**
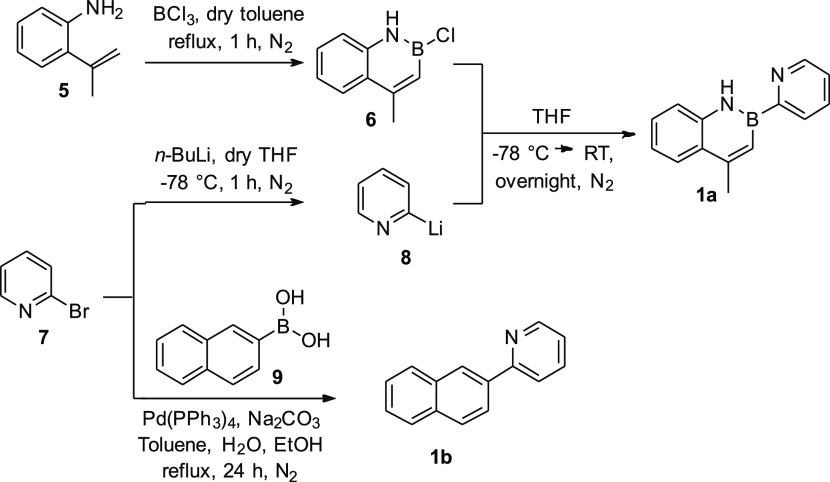
Synthesis of Ligands **1a** and **1b**

The chelating ligand 4-methyl-2-(pyridin-2-yl)-1,2-dihydrobenzo[*e*][1,2]azaborinine **1a** was synthesized by means of a modification of Dewar’s procedure,^13,61^ as represented in Scheme 1. The 1,2-azaborine aromatic ring was obtained through a direct cyclization of 2-isopropenylaniline **5** with BCl_3_ to generate the intermediate **6** that can undergo nucleophilic substitution at the reactive B–Cl unit. 2-Lithiumpyridine **8** was chosen as a nucleophile. Owing to its poor stability, it was prepared in situ by the reaction of 2-bromopyridine **7** with *n*-butyl lithium, under inert nitrogen atmosphere, and immediately reacted with **6** (Scheme 1).

**Scheme 2:**
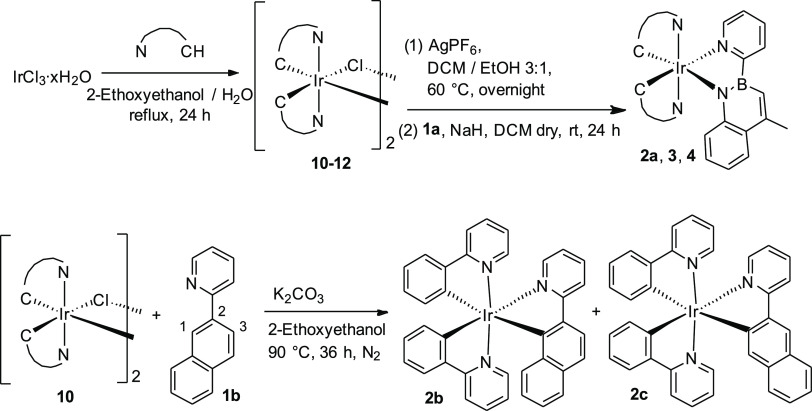
Synthesis of the Azaborine Complexes (**2a**, **3**, and **4**) and Isoelectronic C=C Complexes (**2b** and **2c**)

Ligand **1a** was obtained with good yield (65%) even if the reaction suffers from several problems such as the instability of the pyridine carbanion and the high reactivity of the B–Cl intermediate.^62^ On the other hand, ligand **1b** (*i.e.*, the isoelectronic equivalent of **1a**) was easily obtained through a typical Suzuki reaction, starting from commercially available 2-bromopyridine **7** and 2-naphthylboronic acid **9** (Scheme 1).^37^ To set up a suitable procedure to get complexes **2a**, **3**, and **4**, we prepared cyclometalated μ-dichloro bridged iridium precursors [Ir(C^∧^N)_2_Cl]_2_ (**10**–**12**) following standard procedures by refluxing in a 2-ethoxyethanol/water mixture (3:1) the IrCl_3_·*x*H_2_O salt and the appropriate cyclometalating ligand HC^∧^N (*i.e.*, Hppy = 2-phenylpyridine for **10**, Hdfppy = 2-(2,4-difluoro-phenyl)pyridine for **11**, and Hpqu = 2-methyl-3-phenylquinoxaline for **12**, as reported in Scheme 2).^63,64^

Dimers **10**–**12** were treated with AgPF_6_ to increase their reactivity and then added to a solution of 4-methyl-2-(pyridin-2-yl)-1,2-dihydrobenzo[*e*][1,2]azaborinine **1a**, previously reacted with NaH at room temperature. Such a strong base was necessary since, being the 1,2-azaborine an aromatic ligand, the −NH hydrogen is quite difficult to remove, even because it is involved in a strong hydrogen bond with the nitrogen of the pyridine moiety (see below).

The reaction mixture was stirred at room temperature for 24 h. Eventually, the crude was purified by column chromatography on basic alumina. Finally, precipitation from a hexane/CH_2_Cl_2_ solution gave pure complexes **2a**, **3**, and **4** in good yields (38–61%), which were fully characterized by NMR spectroscopy and mass spectrometry (see the Experimental Section and Figures S4–S13). Although the synthesis of neutral iridium(III) complexes by means of cyclometallation reactions with 2-phenylpyridine derivatives usually requires high temperatures, our methodology avoids such drastic conditions, which the azaborine ligand could not withstand.^65^

With isosteric ligand **1b** in hand, we synthesized the corresponding complexes **2b** and **2c** by a modification of the classical methods reported in the literature (Scheme 2).^65,66^ As a matter of fact, ligand **1b** can undergo cyclometallation in two different positions (*i.e.*, at C-1 and at C-3); therefore, a mixture of complexes is expected. By tuning reaction conditions, it was possible to obtain both isomers and we found that by heating dimer **10** and **1b** in 2-ethoxyethanol at +90 °C for 36 h under an inert atmosphere, a 1:1 mixture of tris-cyclometalated isomers **2b** and **2c** was obtained. The neutral complexes can be separated through accurate column chromatography on deactivated silica gel to give **2b** and **2c** in 32 and 43% yields, respectively. It must be emphasized that, due to the less sterically hindered structure, **2c** is the most thermodynamically stable and **2b** slowly converts into the most stable structural isomer when subjected to more drastic reaction conditions (higher temperature) during the synthesis. Even in this case, they were fully characterized by NMR spectroscopy and mass spectrometry (see the Experimental Section and Figures S14–S17).

### X-ray Characterization

**Figure 1:**
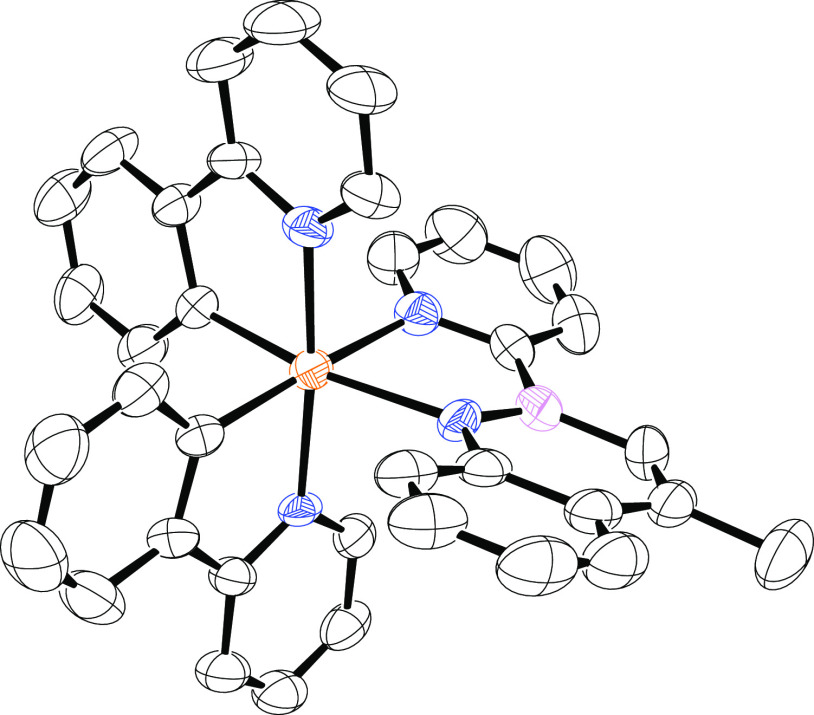
X-ray crystal structure of complex **2a**. Oak Ridge thermal-ellipsoid plot (ORTEP) ellipsoids are at the 50% probability.^67^ Hydrogen atoms are omitted for clarity. Color code: iridium—orange; carbon—black; nitrogen—blue; boron—pink.

Single crystals of complex **2a** were obtained by slow diffusion of ethyl ether in dichloromethane solution. The compound crystallizes in the orthorhombic *Pbcn* space group. The structure showed the arrangement of the three ligands around a pseudo-octahedral iridium ion (Figure 1 and Tables S1–S8). The two nitrogen atoms of the 2-phenylpyridine ligands occupy the two apical positions in a trans arrangement, as in other cyclometalated iridium(III) complexes reported in the literature,^21,32,33^ with N–Ir bond lengths of 2.055 and 2.030 Å. On the other hand, the two remaining adjacent equatorial positions are filled with the two nitrogen of the azaborine ligand; in this case, the N–Ir bonds are longer: 2.184 Å for the azaborine moiety and 2.160 Å for the pyridine one.

The B–N bond length is 1.398 Å, which is slightly shorter than a full-organic azaborine (*i.e.*, 1.427 Å).^13^ The azaborine bite angle (*i.e.*, N–Ir–N angle) is 78.43°, which is comparable to that of the two phenylpyridine ligands (*i.e.*, 80.1 and 79.6°). On the contrary, unlike the phenylpyridines, the azaborine ligand itself is slightly bent (mean distance from ligand least-squares plane: 0.11 *vs* 0.06 Å) and the N–Ir–N–B–C five-membered ring is not planar, with the iridium atom 0.62 Å out of the least-squares plane passing through the ligand. Conversely, the ancillary ligand itself is expected to be planar, corroborating the aromatic character of the borazaronaphthalene moiety (see below).^18^

### DFT Calculations: Ground-State Properties

For a better understanding of the electronic and optical properties of both ligands **1a** and **1b** and the related iridium(III) complexes, DFT and TD-DFT calculations were carried out using the M06 hybrid meta-GGA exchange–correlation functional,^39,40^ together with the polarizable continuum model (PCM) to take into account acetonitrile solvation effects.^44−46^ The validity of the adopted computational method has already been effectively tested on similar systems (as demonstrated by several publications on the topic),^27,32^ and it is also proved by the nearly superimposable geometries of the available X-ray structure of **2a** and its theoretically optimized one (Figure S18).

As far as the ligands are concerned, the main structural difference between such compounds is the unusual full planarity of the azaborine ligand (**1a**), which is estimated to exist merely as a single conformer with the two nitrogen atoms in cis-arrangement (Figure S19). On the contrary, the naphthyl-pyridine analogue (**1b**) is nonplanar, as commonly expected for other phenylpyridine derivatives,^68^ showing two nearly isoenergetic conformers with a dihedral angle of ±20° between its two aromatic rings (Figure S19). Figures S20 and S21 show the energy diagrams and the frontier molecular orbitals of both ligands (considering different conformers), also evaluating the effect of the methyl substituent, which is present in the azaborine ligand **1a**, but not in **1b**. The HOMO–LUMO gap is wider in **1a**, due to both HOMO stabilization and LUMO destabilization (*i.e.*, 4.96 *vs* 4.77 eV for **1a***vs***1b**, respectively—Figure S20). No significant differences were appreciated by comparing the two nearly isoenergetic **1b** conformers (*i.e*., **1b**_**A**_ or **1b**_**B**_—Figure S21). On the contrary, the presence of the methyl substituent on the naphthyl ring of **1b** (as in the theoretical ligand **1b′**) leads to a 0.10 eV destabilization of the HOMO, which is mainly located on such polycyclic moiety, leaving the LUMO virtually unperturbed (Figure S21); taking the methyl effect into consideration, our results suggest that the replacement of the C=C bond with the isoelectronic B–N one is potentially able to stabilize the HOMO by more than 0.2 eV.

**Figure 2:**
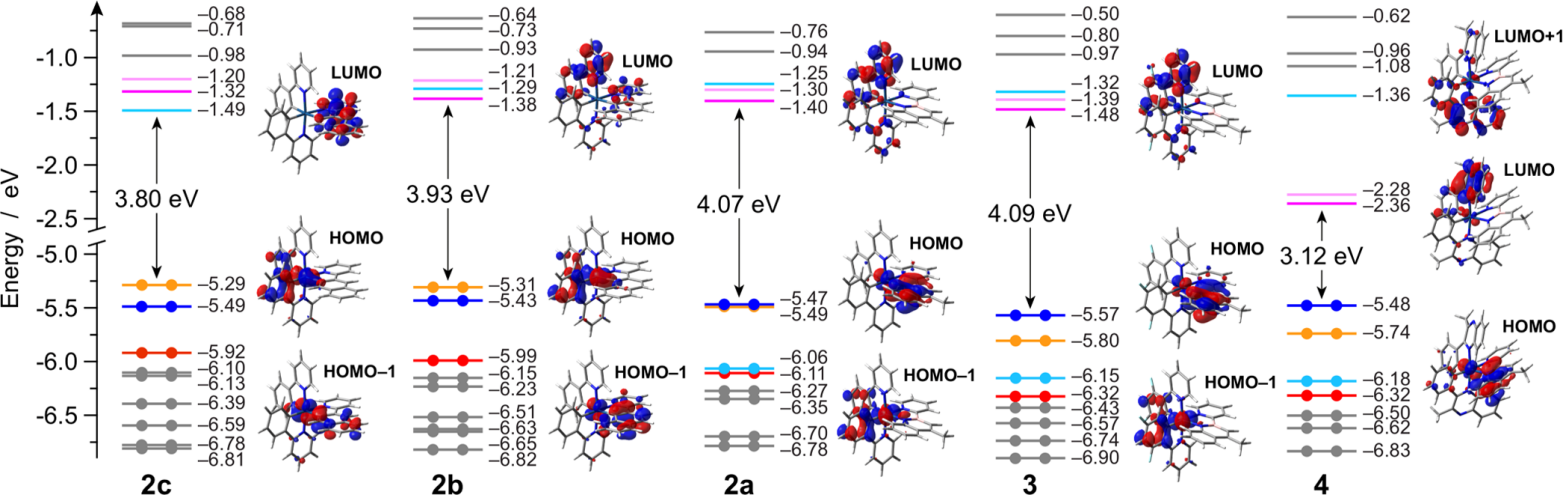
Energy diagram showing the frontier Kohn–Sham molecular orbitals of **2**–**4** in acetonitrile. For some relevant orbitals, the corresponding isosurface is also displayed for the sake of clarity (isovalue = 0.04 e^1/2^ b^–3/2^). Along the series, relevant orbitals with similar topologies are plotted with the same color for an easier comparison.

Concerning the iridium(III) complexes, the energy diagrams and the frontier molecular orbitals (FMOs) of the azaborine-based complexes **2a**, **3**, and **4** are reported in Figure 2, together with those of the isoelectronic C=C analogues **2b** and **2c**. As done for the ligands, also the methylated analogue of complex **2b** was investigated (*i.e.*, **2b′**, see Figure S22). As already observed for **1b***vs***1b′**, only the destabilization of the occupied orbital on the naphthyl ring is observed upon methylation, but such an orbital is the HOMO–1 in the complexes so that the HOMO–LUMO gaps is virtually identical in both **2b** and **2b′**.

Comparing all of the synthesized complexes, a rather complicated scenario is observed due to a tricky orbital interplay within sets of nearby occupied and unoccupied FMOs. In the case of azaborine-based complexes, the HOMO is always predominantly centered on the borazaronaphthalene ligand and the HOMO–1 is mainly located on the iridium d orbitals and on the phenyl moieties of the cyclometalating ligands. On the contrary, the scenario is reversed when the azaborine ligand is replaced with its C=C analogue (as in **2b** and **2c**, Figure 2). Such an interplay between this couple of orbitals is due to the different energy of the orbital centered on the iridium ion and on the phenyl moieties of the cyclometalating ligands, while the highest π orbital located on the ancillary ligand (*i.e.*, the borazaronaphthalen- or naphthalen-pyridine) remains energetically unaffected along all of the series at −5.49 ± 0.05 eV. Indeed, the former orbital is highly stabilized in complex **3** (by 0.31 eV, if compared to **2a**) due to the presence of the electron-withdrawing fluorine substituents,^32^ while it is destabilized by approx. 0.2 eV in **2b** and **2c**, due to the stronger field exerted by the cyclometalating C=C ancillary ligand. Notably, in the case of **2a**, such pairs of orbitals are virtually isoenergetic.

As far as unoccupied FMOs are concerned, analogous sets of three nearby π* orbitals can be identified along all of the complexes of the series (Figure 2). For azaborine-based derivatives **2a** and **3**, LUMO and LUMO+1 are located on each of the pyridine moieties of the two ppy-based ligands, while LUMO+2 is accommodated on the pyridine part of the azaborine ligand. Only a minor overall stabilization of this set of orbitals is observed by passing from **2a** to **3** (approx. 0.08 eV), due to the effect of the electron-withdrawing fluorine substituents.^32^ Notably, although the same orbital order is also preserved in **4**, a remarkable LUMO and LUMO+1 stabilization of ≈0.97 eV (compared to **2a**) is observed due to the replacement of the pyridine moiety on the cyclometalating ligands with the π-extended quinoxaline one. On the contrary, the stabilization on LUMO+2 is only 0.11 eV since centered on the same azaborine ligand.

If comparing **2a** with its isoelectronic C=C analogues **2b** and **2c**, a progressive destabilization of the π* orbitals centered on the ppy ligands is observed, with a concomitant stabilization of those located on the azaborine-based ligand (in **2a**) or its C=C analogue (in **2b** and **2c**). As a result, the LUMO remains centered on the pyridine moiety of the ppy ligand in **2a** and **2b**, but it is located at lower energy and on the naphthyl-pyridine ligand in **2c**.

### Electrochemistry

The scenario envisaged by DFT calculations is substantially confirmed by electrochemical experiments, carried out using square-wave and cyclic voltammetry in acetonitrile solution at ambient temperature (see the Experimental Section for further details). Selected electrochemical data are summarized in Table 1, whereas the square-wave voltammograms of ligands **1a** and **1b** and of all of the investigated complexes are reported in Figures S23 and 3, respectively.

**Figure 3:**
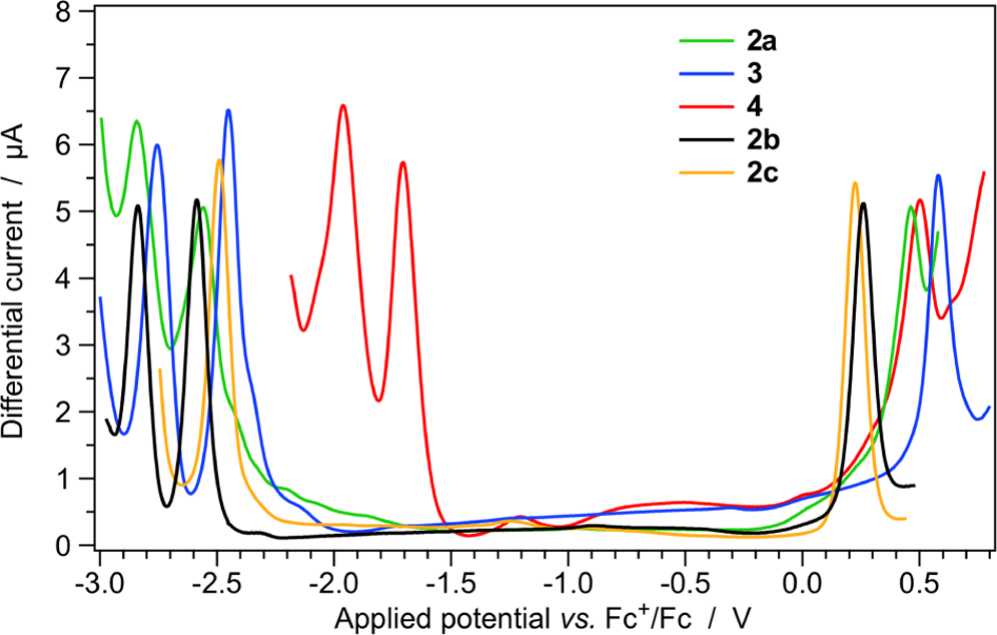
Square-wave voltammograms of the azaborine-based complexes **2a**, **3**, and **4** (0.5 mM) in room-temperature acetonitrile solution, together with those of the isoelectronic C=C complexes **2b** and **2c**.

**Table 1:** Electrochemical Data in Acetonitrile Solution at 298 K and DFT-Calculated HOMO–LUMO Energy Levels

	electrochemical data^a^ (V)			DFT data^c^ (eV)		
	*E*_ox_	*E*_red_	Δ*E*_redox_^b^	*E*_HOMO_	*E*_LUMO_	Δ*E*_DFT_^d^
**1a**	+1.18	–2.59	3.77	–6.24	–1.28	4.96
**1b**	+1.28^rev.^	–2.60^rev.^	3.88	–6.15	–1.36	4.79
**2a**	+0.46	–2.56	3.02	–5.47	–1.40	4.07
**3**	+0.58	–2.45	3.03	–5.57	–1.48	4.09
**4**	+0.50	–1.71	2.21	–5.48	–2.36	3.12
**2b**	+0.25^rev.^	–2.59^rev.^	2.84	–5.31	–1.38	3.93
**2c**	+0.23^rev.^	–2.49^rev.^	2.72	–5.29	–1.49	3.80

a The reported potentials are obtained by square-wave voltammetry and reported *vs* the ferrocene/ferrocenium couple, used as internal reference. All redox processes are irreversible, if not otherwise stated (rev.).

b Δ*E*_redox_ = *E*_ox_ – *E*_red_.

c DFT calculations were performed at the PCM-M06 level of theory in acetonitrile.

d Δ*E*_DFT_ = *E*_HOMO_ – *E*_LUMO_.

None of the redox processes exhibited by the borazaronaphthalene free ligand **1a** or by its corresponding complexes (**2a**, **3**, and **4**) is reversible, as also found for other similar azaborine systems.^69^ On the contrary, full electrochemical reversibility is observed for all of the first oxidation and reduction processes occurring in the isoelectronic C=C ligand **1b** and in the associated complexes **2b** and **2c**, as commonly observed in cyclometalated iridium(III) complexes (Figure S24).^32,33^

The oxidation potentials of all three azaborine-based complexes are comparable, with nearly identical values for **2a** and **4** (*i.e.*, +0.46 and +0.50 V, respectively) and a 0.1 V anodically shifted potential for **3**, due to electron-withdrawing fluorine substituents. Such experimental evidences are in excellent agreement with DFT predictions, expecting virtually identical HOMO topologies for all of the three complexes and a 0.10 eV stabilization in **3**, compared to **2a** (see above). On the other hand, the oxidation of the two C=C isoelectronic complexes (**2b** and **2c**) occurs at much lower values compared to the **2a** analogue, with the oxidation potential of **2b** being slightly more positive than that of **2c** (*i.e.*, +0.25 *vs* +0.23 V, respectively). Again, the correlation with DFT estimates is impressive: (i) the calculated HOMO destabilization in **2b** and **2c**, with respect to **2a**, is just slightly underestimated if compared to the corresponding cathodic shift in their oxidation potential (i.e., 0.17 eV *vs* 0.22 V); (ii) the higher oxidation potential of **2b**, if compared to **2c**, is perfectly justified by the 0.02 eV more stabilized HOMO of the former complex.

As far as reductions are concerned, as predicted by DFT, far less negative potentials are observed for complex **4**, due to the presence of the easily reducible π-extended quinoxaline moiety on each of the two cyclometalating ligands; indeed, two reduction processes are observed at −1.71 and −1.96 V (Figure 3). At more negative potentials, the two reduction peaks of complexes **2a** and **3** are detected, which are attributable to the reduction of the pyridine moiety on each of the two cyclometalating ligands, in analogy to **4**. As normally observed for fluorinated complexes,^21,32^ the two peaks of complex **3** are shifted to more positive values by 0.10 eV compared to fluorine-free analogue **2a**, in line with DFT calculations (Figure 2).

On the other hand, the replacement of the azaborine ancillary ligand with the C=C counterpart does not significantly alter the reduction potential of **2b** and only a minor cathodic shift of 0.03 V is observed compared to **2a** (Table 1); this can be easily rationalized by considering that the first reduction is expected to involve the same pyridine moiety of the ppy ligand, which is identical in both **2b** and **2a**. On the contrary, the different chelation mode of the C=C ancillary ligand in **2c** results in a more positive reduction potential by 0.10 V, compared to **2b** (Table 1). This is because such reduction now involves the highly stabilized π* orbital of the ancillary ligand itself and not the ppy ligand (Figure 2). All of these results are perfectly caught by DFT calculations, which estimate a 0.02 eV LUMO destabilization by passing from **2a** to **2b** and a 0.11 eV LUMO stabilization if **2b** is compared to **2c** (Table 1 and Figure 2).

### Photophysical Characterization of the Ligands

**Figure 4:**
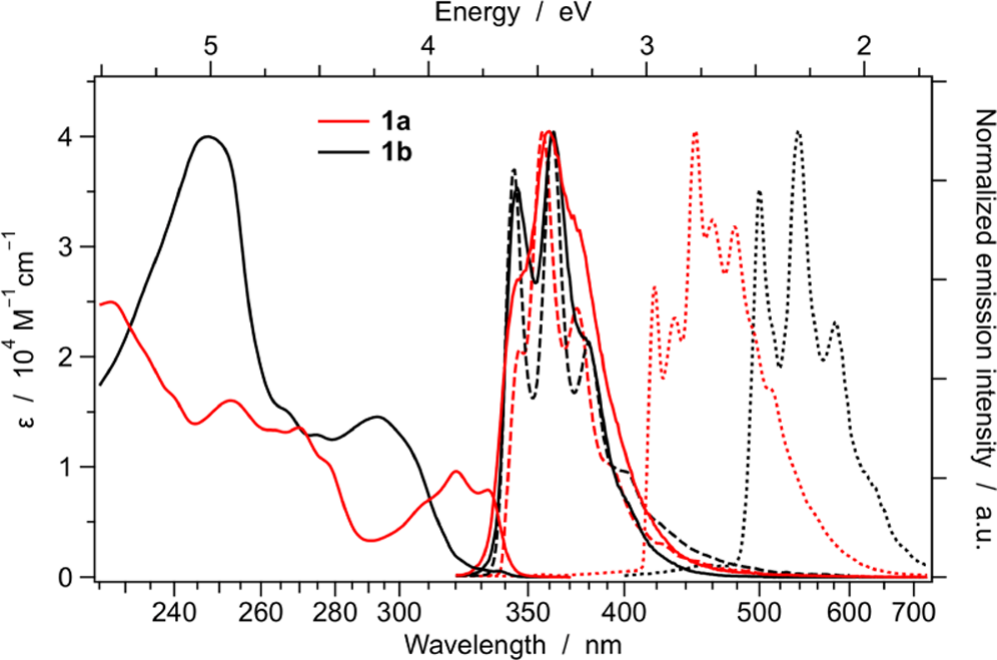
Absorption and fluorescence spectra of **1a** and **1b** in acetonitrile solution at 298 K (full). Spectra in butyronitrile at 77 K are also reported: fluorescence (dashed) and phosphorescence (dotted).

The photophysical properties of the azaborine free ligand **1a** were investigated in room-temperature solution and in rigid matrix at 77 K, and compared to that of the C=C isoelectronic analogue **1b**. The room-temperature absorption and emission spectra of **1a** and **1b** in acetonitrile solution are reported in Figure 4, together with fluorescence and phosphorescence spectra in the rigid matrix at 77 K. The absorption and emission spectra of both ligands are only marginally affected by the solvent polarity, and only a minor red-shift of the absorption profiles is observed by passing from more polar acetonitrile solutions to dichloromethane (Figure S25).

The absorption profile of the azaborine ligand **1a** strongly differs from the one of **1b**, mainly for the lack of the intense absorption band present for the C=C analogue and centered at approx. 250 nm. In addition, the lowest-energy S_0_ → S_1_ transition of **1a** is vibronically structured and rather intense (ε = 9.6 × 10^3^ M^–1^ cm^–1^) with maximum at 320 nm (Figure 4); on the contrary, **1b** displays a similar transition in the same spectral region but with a much weaker profile (ε ≈ 6 × 10^2^ M^–1^ cm^–1^), which is partially covered by a more intense unstructured band centered at 293 nm (Figure 4).

Surprisingly, TD-DFT calculations estimate S_0_ → S_1_ excitations with considerable HOMO → LUMO character and high oscillator strengths for both **1a** and **1b** (*i.e.*, *f* = 0.405 and 0.208, Table S9), which is in contrast to the experimental absorption spectrum of **1b**. Indeed, more advanced STEOM-DLPNO-CCSD calculations reveal that the adopted TD-DFT method fails to predict the correct excited-state order in **1b** (compare Tables S9 and S10). In the case of the azaborine ligand **1a**, both methods agree that the population of S_1_ occurs by an intense π–π* HOMO → LUMO transition having a minor charge-transfer nature from the borazaronaphthalene moiety to the pyridine ring; such vertical excitation is estimated at 303 nm by TD-DFT approach and at 310 nm for the CCSD-based calculation (Tables S9 and S10). Moreover, if vibronic effects are taken into account, the agreement between the lowest-energy absorption band of the experimental spectrum of **1a** in acetonitrile and the corresponding TD-DFT vibronically resolved S_0_ → S_1_ transition is remarkable (Figure S26). Indeed, the computed absorption band is underestimated by approx. 0.05 eV and the vibronic progression is nicely reproduced. The highly structured profile observed for such a lowest-energy band is due to the very similar geometry adopted by **1a** in both S_0_ and S_1_ (Figure S27). In addition, both TD-DFT and STEOM-DLPNO-CCSD methods agree in assigning the S_0_ → S_2_ excitation to a weak n−π* transition (*f* = 0.004) involving the pyridine moiety.

On the other hand, in the C=C isoelectronic analogue **1b**, there are two lowest-lying excited states with π–π* character (with different charge-transfer contributions) and the n−π* transition involving the pyridine moiety is the S_0_ → S_3_ excitation. Anyway, only STEOM-DLPNO-CCSD is able to correctly predict that the S_0_ → S_1_ transition is weakly absorbing (*f* = 0.001 at 318 nm) and the S_0_ → S_2_ is the bright one (*f* = 0.316 at 281 nm); such states are energetically reversed by TD-DFT (compare Tables S9 and S10), leading to the incorrect prediction of the absorption spectrum of **1b**. A possible explanation for such failure can be ascribed to the well-known underestimation of excitation energies that TD-DFT methods suffer in the case of charge-transfer transitions.^70^ The lack of vibronic progressions in the absorption spectrum of **1b** can be tentatively ascribed to pronounced structural rearrangements upon excitation. Indeed, **1b** is not planar in the ground state (Figure S19), while planarization is expected for all of the lowest excitations due to the population of a C=C bonding orbital between the naphthalene and pyridine rings (Tables S9 and S10).

Despite the differences in their absorption profiles, **1a** and **1b** ligands present very similar fluorescence spectra, with rather structured emission profiles centered at approx. 360 nm, regardless of solvent polarity (Figures 4 and S25 and Table 2). Even fluorescence quantum yields are virtually identical for both **1a** and **1b** (*i.e.*, around 20 and 25% in acetonitrile and dichloromethane solutions, respectively), despite the azaborine ligand **1a** displaying a 10 times shorter excited-state lifetime with respect to the C=C isoelectronic analogue **1b** (*i.e.*, τ ≈ 1 *vs* 10 ns, Table 2).

**Table 2:** Luminescence Properties and Photophysical Parameters of the Ligands^e^

	CH_3_CN solution, 298 K					CH_2_Cl_2_ solution, 298 K					BuCN rigid matrix, 77 K	
	λ_em_^a^	PLQY^a^	τ^b^	*k*_r_^c^	*k*_nr_^d^	λ_em_^a^	PLQY^a^	τ^b^	*k*_r_^c^	*k*_nr_^d^	λ_em_^a^	τ^b^
	(nm)	(%)	(ns)	(10^7^ s^–1^)	(10^7^ s^–1^)	(nm)	(%)	(ns)	(10^7^ s^–1^)	(10^7^ s^–1^)	(nm)	(ns)
**1a**	347^sh^, 359, 372^sh^	21.5	1.3	16	59	348^sh^, 362, 376^sh^	27.8	1.4	20	51	347^sh^, 357, 374, 391^sh^	2.3
**1b**	345, 362, 376^sh^	19.3	9.8	2.0	8.3	346, 363, 380, 403^sh^	25.4	11.9	2.1	6.3	344, 361, 380, 400^sh^	12.1

a λ_exc_ = 310 nm.

b λ_exc_ = 280 nm.

c Radiative constant: *k*_r_ = PLQY/τ.

d Nonradiative constant: *k*_nr_ = 1/τ – *k*_r_.

e sh, shoulder.

The 77 K emission spectra of both **1a** and **1b** in butyronitrile frozen glass show the presence of fluorescence and phosphorescence (Figure 4). For both ligands, the fluorescence spectrum at 77 K exhibits just a more vibrationally resolved structure, but it is virtually superimposable with the one at room temperature. If **1a** and **1b** display an S_1_ state with comparable energy, this is not the case for their lowest triplet: the phosphorescence spectrum of the azaborine ligand **1a** is considerably blue-shifted with respect to the one of its C=C analogue **1b** (Figure 4). Indeed, unrestricted DFT calculations confirm the different nature of such triplets, despite both displaying a fully planar geometry, as observed for their lowest singlets. In the case of **1a**, T_1_ is fully centered on the borazaronaphthalene moiety, while, for the triplet of **1b**, the spin-density distribution is also spread on the pyridine ring (Figure S28). This is reflected in the different phosphorescence lifetime of these molecules (*i.e.*, 2.60 *vs* 0.90 s for **1a***vs***1b**, see Table S11) and in a drastically dissimilar vibronic progression of the two phosphorescence spectra, which are nicely reproduced by vibronically resolved DFT calculations (Figure S29).

### Photophysical Characterization of the Complexes

**Figure 5:**
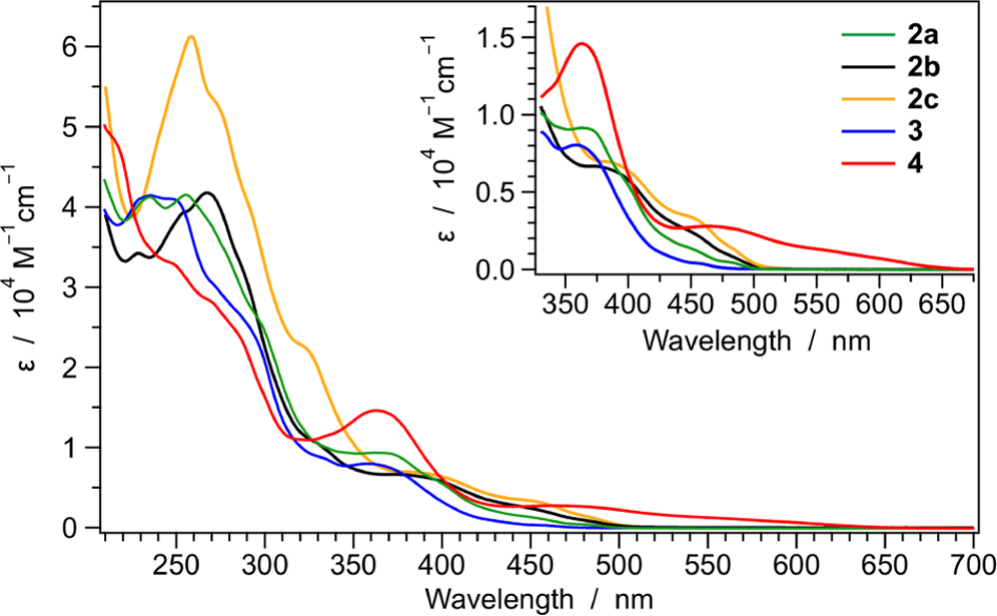
Absorption spectra of azaborine-based complexes (**2a**, **3**, and **4**) together with the ones of the C=C analogues (**2b** and **2c**). Spectra are recorded in acetonitrile at 298 K; lowest-energy transitions are magnified in the inset.

All of the iridium(III) complexes were studied in solution at room temperature (CH_3_CN and CH_2_Cl_2_), in butyronitrile glass at 77 K and in PMMA matrix at a sample concentration of 1% by weight. The absorption spectra of all of the complexes in acetonitrile and dichloromethane solutions are reported in Figures 5 and S30, respectively; no notable changes are observed by changing the polarity of the solvent.

As typically found in cyclometalated iridium(III) complexes, the main absorption bands in the spectral region between 200 and 300 nm are attributed to spin-allowed ligand-centered (LC) π–π* transitions. At longer wavelengths (300–400 nm), the weaker and broader bands can be assigned to charge-transfer transitions with mixed metal-to-ligand, ligand-to-ligand, and intraligand charge-transfer (MLCT/LLCT/ILCT) nature of different multiplicity;^21,32^ an exception to this general assignment is the band peaking at 370 nm in the absorption spectrum of complex **4**, which is attributed to the quinoxaline moiety.^71^ The lowest-energy absorption profiles of all of the complexes are magnified in the inset of Figure 5, and it should be emphasized that such low-energy bands are virtually insensitive to solvent polarity (Figure S30), suggesting a predominantly ligand-centered nature of the lowest-lying triplet states. The lowest-energy absorption profiles of **2a** and **3** are similar, but the latter is blue-shifted due to the electron-withdrawing fluorine substituents on the cyclometalating ligands.^32^ On the contrary, as already suggested by DFT calculations and electrochemical measurements, the spectral profiles of the C=C complexes (**2b** and **2c**) extend to longer wavelengths compared to the azaborine-based analogue **2a**.

**Figure 6:**
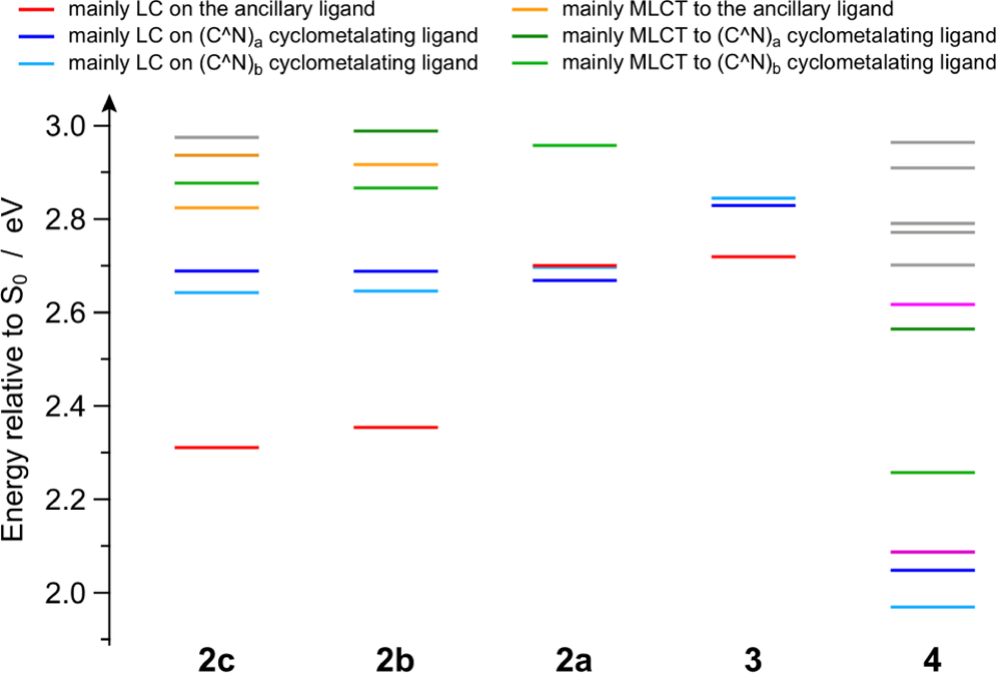
Energy diagram of the lowest-lying triplet states (below 3.00 eV) for all of the complexes, computed in acetonitrile as vertical excitations from the respective ground-state minimum-energy geometries.

To get a deeper insight into the excited-state properties of all of the complexes, the lowest-lying triplet states were computed by means of TD-DFT methods. Tables S12–S16 illustrate the lowest triplet excitations of **2a**–**c**, **3**, and **4**, reported as couples of natural transition orbital (NTO).^55^ Moreover, a compact representation of triplet-state energy landscape at the Franck–Condon region is shown in Figure 6 for all of the investigated complexes. The most complicated excited-state scenario is expected for complex **2a**, due to the presence of three nearly isoenergetic triplets at 2.67, 2.69, and 2.70 eV, which are all ^3^LC states, respectively, localized on the two nonequivalent ppy ligands and on the azaborine ancillary one (Table S12). In the case of **3**, the presence of the fluorine substituents on the cyclometalating ligands increases the energy of the corresponding ^3^LC states by 0.16 eV, while keeping the one centered on the azaborine relatively unperturbed (*i.e.*, 2.72 *vs* 2.70 eV in **2a**, Tables S12 and S13). The scenario is reversed in the case of the isoelectronic C=C analogues **2b** and **2c**, which have the same ppy cyclometalating ligands of **2a**: for both complexes, the two nearly degenerate ^3^LC states localized on the two ppy moieties remain unperturbed at 2.67 ± 0.02 eV, as in **2a**, but a substantial stabilization of the triplet state centered on the C=C ancillary ligand is calculated, and it is expected to be maximized in **2c** (Tables S15 and S16). As a consequence, although for different reasons, complexes **2b**, **2c**, and **3** are all expected to emit from a ^3^LC state centered on the ancillary ligand, which is the lowest triplet. On the contrary, the two lowest triplets in **4** are ^3^LC states centered on each of the two cyclometalating ligands, which are strongly stabilized with respect to **2a** due to the π-extended quinoxaline moiety and lay only 1.97 and 2.05 eV above S_0_ (Table S14).

**Figure 7:**
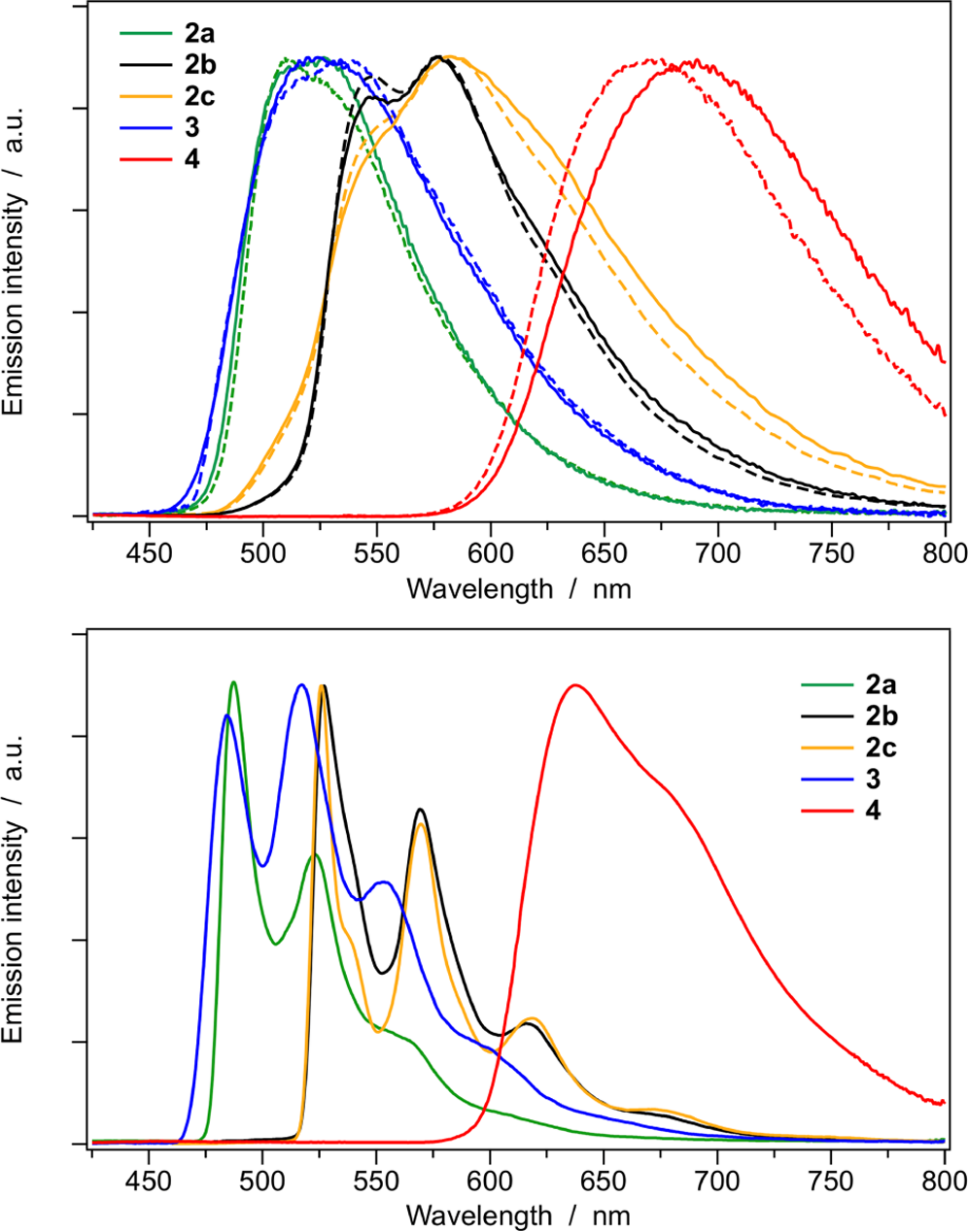
Normalized emission spectra of all of the complexes in acetonitrile (solid) and in dichloromethane (dashed) solutions at 298 K (top) and in butyronitrile glass at 77 K (bottom).

**Figure 8:**
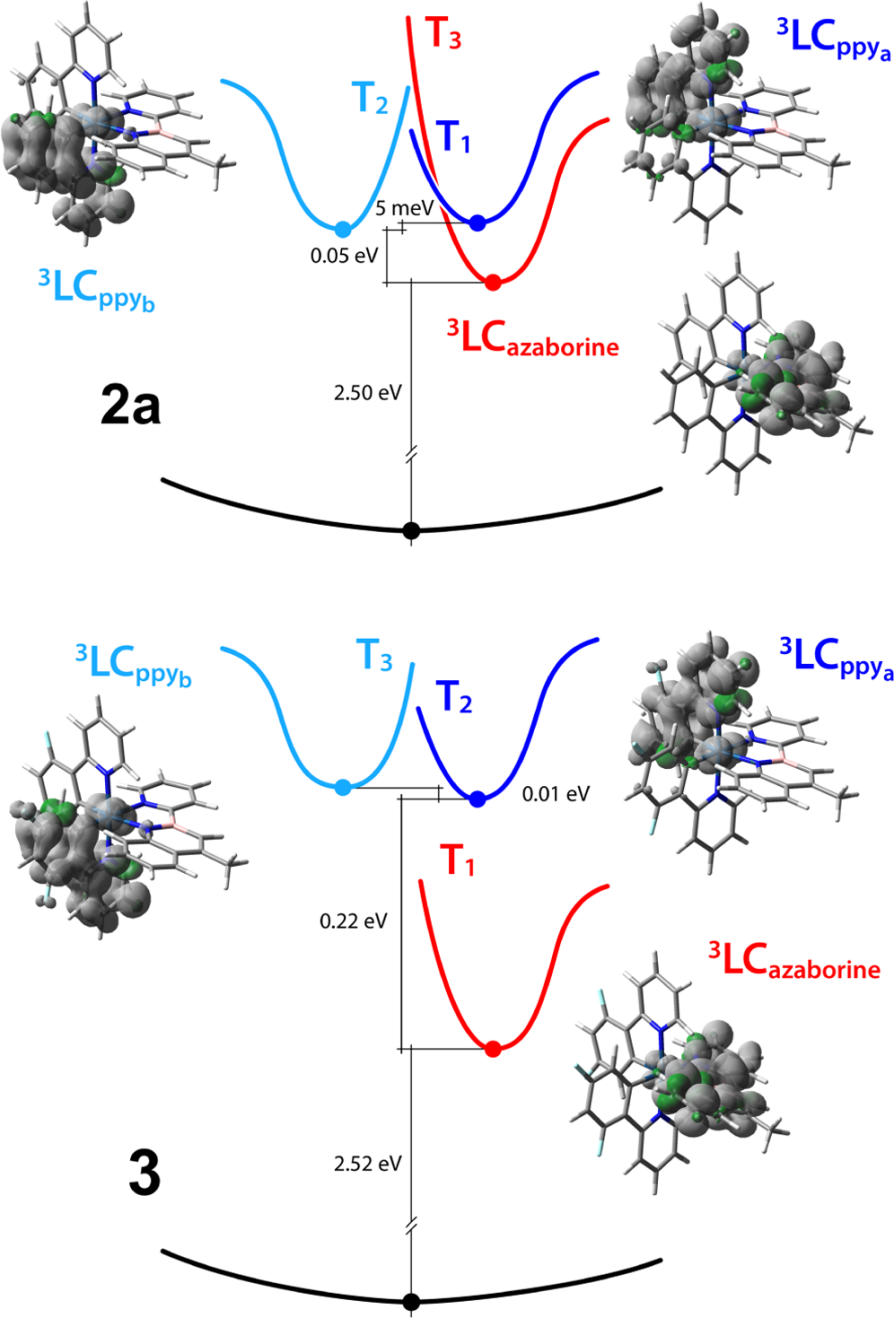
Schematic energy diagram of complexes **2a** (top) and **3** (bottom) showing the adiabatic energy differences between S_0_ and their lowest-energy fully relaxed triplet states (full dots). The unpaired-electron spin-density surfaces calculated at the triplet-state minima are also depicted (isosurfaces: 0.002 e bohr^–3^).

Normalized emission spectra of all of the complexes are reported in Figure 7 in room-temperature acetonitrile and dichloromethane solutions (top), and in butyronitrile glass at 77 K (bottom). The corresponding luminescence properties and photophysical parameters are summarized in Table 3. The emission spectra of the azaborine-based complexes **2a** and **3** are nearly superimposable, with emission maxima at 523 and 524 nm in room-temperature acetonitrile solution (Table 3). At 298 K, the emission bands of both complexes are only marginally affected by changing the solvent polarity and exhibit clues of vibronic progression (Figure 7, top). At 77 K, the spectra of both **2a** and **3** become strongly structured, but their band onset does not shift compared to room temperature. These experimental findings suggest that the emission arises from a ^3^LC state with a virtually identical nature in both complexes. Indeed, spin-unrestricted DFT optimizations carried out on the lowest triplet states of **2a** and **3** demonstrate that, after relaxation, the lowest triplet state of both complexes is the ^3^LC state centered on the azaborine ligand, which will be ultimately responsible for emission (Figure 8). The adiabatic energy difference between such ^3^LC state and S_0_ is estimated to be 2.50 and 2.52 eV for **2a** and **3**, respectively, which is in good agreement with the emission maxima in acetonitrile solution at 298 K (*i.e.*, approx. 2.37 eV, see Table 3).

**Table 3:** Luminescence Properties and Photophysical Parameters of All of the Investigated Complexes^e^

	CH_3_CN solution, 298 K					CH_2_Cl_2_ solution, 298 K					BuCN rigid matrix, 77 K	
	λ_em_^a^ (nm)	PLQY^a^ (%)	τ^b^ (μs)	*k*_r_^c^ (10^4^ s^–1^)	*k*_nr_^d^ (10^5^ s^–1^)	λ_em_^a^ (nm)	PLQY^a^ (%)	τ^b^ (μs)	*k*_r_^c^ (10^4^ s^–1^)	*k*_nr_^d^ (10^5^ s^–1^)	λ_em_^a^ (nm)	τ^b^ (μs)
**2a**	523	15.9	1.6	9.9	5.3	510, 530^sh^	19.2	1.3	14.8	6.2	490, 526, 566	5.9
**3**	524	5.7	1.7	3.4	5.6	545	5.7	1.9	3.0	5.0	484, 516, 556, 598^sh^	21.8
**4**	692	6.5	1.4	4.6	6.7	672	12.0	1.5	8.0	5.9	638, 670^sh^	10.7
**2b**	548, 577	21.5	4.3	5.0	1.8	549, 578	38.8	6.7	5.8	0.91	527, 570, 617, 669^sh^	14.9
**2c**	551^sh^, 583	6.0	1.4	4.3	6.7	552^sh^, 583	8.4	2.6	3.2	3.5	526, 570, 619, 670^sh^	19.3

a λ_exc_ = 450 nm.

b λ_exc_ = 370 nm.

c Radiative constant: *k*_r_ = PLQY/τ.

d Nonradiative constant: *k*_nr_ = 1/τ – *k*_r_.

e sh, shoulder.

It should be noted that, for the fluorinated complex **3**, the order of the three lowest triplet states (T_1_–T_3_) is preserved upon relaxation; indeed, the energy gap between the lowest ^3^LC state centered on the azaborine ligand (*i.e.*, T_1_) and the upper-lying ones located on the two dfppy ligands (*i.e.*, T_2_ and T_3_) increases from 0.12 (at the Franck–Condon region, Table S13) to 0.22 eV (as adiabatic energy difference after triplet relaxation, Figure 8).

On the other hand, a state flipping is observed in the case of **2a**, having nearly isoenergetic T_1_–T_3_ at the Franck–Condon region (Table S12). Due to a larger geometrical relaxation of the ^3^LC state centered on the azaborine ligand (*i.e.*, T_3_), this triplet becomes 0.05 eV lower in energy compared to the ones located on the two ppy ligands (*i.e.*, T_1_ and T_2_, Figure 8). Actually, due to the small energy gap between these three states, a thermal equilibration could not be ruled out and this can possibly explain the higher photoluminescence quantum yield of **2a** compared to **3** (*i.e.*, approx. 20 *vs* 6%, Table 3), with faster radiative decay constants (*i.e.*, *k*_r_ ≈ 1 × 10^5^*vs* 3 × 10^4^ for **2a** and **3**, respectively).

In the case of complex **4**, its room-temperature emission band shows a broad and unstructured profile, and it is strongly red-shifted compared to **2a** and **3**. Notably, such red-shift is more pronounced as the solvent polarity increases (*i.e.*, λ_em_ = 672 *vs* 692 nm in dichloromethane *vs* acetonitrile solution). Moreover, a rigidochromic shift is also observed in frozen matrix at 77 K (*i.e.*, λ_em_ = 638 nm, Table 3). Such experimental findings suggest an emission from a triplet state with a high charge-transfer character, but the relatively long excited-state lifetime at 77 K (*i.e.*, 10.7 μs, Table 3) suggests a more ligand-centered emission. Indeed, a clear attribution of the emitting state is difficult since the three lowest triplet states of **4** are calculated to be isoenergetic after relaxation (*i.e.*, Δ*E* < 5 meV); most probably, a thermal equilibrium between T_1_ and T_3_ exists at room temperature, while the ^3^LC state (*i.e.*, T_2_) can be predominant at 77 K, due to the lack of solvent reorganization supporting the full stabilization of the other two triplets with more charge-transfer character.

Last but not least, the emission properties of the azaborine-based complex **2a** can be compared with that of the two isoelectronic C=C analogues (**2b** and **2c**). U-DFT calculations indicate that, upon full geometrical relaxation, the lowest triplet state of **2b** and **2c** remains T_1_ (Figure S31), so emission is expected to arise from such ^3^LC state centered on the naphthalene–pyridine ancillary ligand in both complexes. Such a state is estimated to be located at 2.29 and 2.32 eV above S_0_ for **2b** and **2c**, respectively (to be compared to 2.50 eV for **2a**, Figure 8). Indeed, a red-shift of 0.18 eV is experimentally observed in the emission profiles **2b** and **2c**, if compared to **2a** (Figure 7). The photophysical properties of the two C=C isomers are very similar and lower photoluminescence quantum yields are only observed for **2c**, basically due to faster nonradiative deactivation pathways (*e.g.*, PLQY = 38.8 *vs* 8.4% for **2b***vs***2c** in oxygen-free CH_2_Cl_2_ solutions at 298 K, Table 3).

The emission properties of the complexes were also investigated in the solid state, that is, dispersed in a poly(methyl methacrylate) (PMMA) matrix at a sample concentration of 1% by weight. The corresponding emission spectra are reported in Figure S32, and the photophysical parameters are gathered in Table 4.

**Table 4:** Luminescence Properties and Photophysical Parameters of All of the Investigated Complexes in 1% PMMA Matrix at 298 K^e^

	1% PMMA matrix				
	λ_em_^a^ (nm)	PLQY^a^ (%)	τ^b^ (μs)	*k*_r_^c^ (10^4^ s^–1^)	*k*_nr_^d^ (10^4^ s^–1^)
**2a**	512, 530^sh^	67.4	3.5	19.3	9.3
**3**	508, 532	68.9	14.2	4.9	2.2
**4**	672	22.3	1.3	17.2	59.8
**2b**	541, 576, 620^sh^	49.6	8.3	6.0	6.1
**2c**	539, 575, 620^sh^	27.1	4.7	5.8	15.5

a λ_exc_ = 450 nm. Photoluminescence quantum yield determined by integrating sphere.

b λ_exc_ = 370 nm.

c Radiative constant: *k*_r_ = PLQY/τ.

d Nonradiative constant: *k*_nr_ = 1/τ – *k*_r_.

e sh, shoulder

A strong enhancement of the luminescence is observed for all of the complexes once dispersed in PMMA (*i.e.*, PLQY = 22–67%, Table 4). Except for the complicated case of **4** (where a complex interplay between nearby triplets is expected, see above), for all other complexes, the enhancement in PLQYs is basically due to a strong reduction of the *k*_nr_ values, due to the rigidity of the polymeric matrix that is able to reduce nonradiative deactivation channels.

## Conclusions

Neutral luminescent iridium(III) complexes carrying a 1,2-azaborine as B–N alternatives to commonly used C=C cyclometalating ligand have been obtained for the first time. An efficient procedure for the synthesis of 2-(pyridin-2-yl)-2,1-borazaronaphthalene **1a** has been set up, and this novel compound has been exploited as a ligand to introduce a new class of neutral phosphorescent iridium(III) complexes equipped with different cyclometalating ligands. Such complexes (**2a**, **3**, and **4**) have been obtained in satisfactory yields and fully characterized. In addition, stereoisomeric complexes (**2b** and **2c**) containing the isoelectronic C=C cyclometalating 2-(pyridin-2-yl)-naphthalene ligand **1b** have been synthesized and compared with the analogue **2a**. The aromatic character of the ligand is maintained despite the B–N bond, and its rigidity seems to effectively limit distortions and nonradiative deactivation processes of the excited state.

The azaborine ligand **1a** displays similar luminescence properties if compared with the isoelectronic C=C analogue **1b** (*e.g.*, λ_max_ = 359 *vs* 362 nm, PLQY = 22 *vs* 19% in acetonitrile solution at 298 K); however, they display very different absorption profiles and excited-state lifetimes (*e.g.*, τ = 1 *vs* 10 ns). The neutral azaborine complexes exhibit good luminescence properties in solution (PLQY up to 19% for **2a**), which are further enhanced in PMMA matrix (PLQY = 22–69%). Except for **4** (which is equipped with highly π-extended cyclometalating ligands), the emission of the azaborine-based complexes arises from ^3^LC states centered on the azaborine ligand itself. The comparison of the electronic properties of **2a** with the two analogous complexes bearing the C=C ligand **1b** showed that the presence of the B–N polarized bond induces a larger band gap than in **2b** or **2c**, resulting in increased redox gap (due to HOMO stabilization) and to blue-shifted emission bands; however, the nature of the emitting state is preserved. The photoluminescence quantum yields and lifetimes of the azaborine-based complex **2a** are comparable with those of the standard C=C counterparts, proving the effectiveness of this approach.

This work introduces a new class of luminescent iridium(III) complexes, which might be expanded by suitably designed 1,2-azaborines to vary the luminescence properties and potential applications thereof.

## Supporting Information Available

NMR spectra of ligands **1** and complexes **2**–**4**; crystal data of compound **2a**; supporting figures and tables, including square-wave voltammograms of ligands **1** and complexes **2a** and **2b**; DFT calculations; and photophysics for ligands **1** and complexes **2**–**4** (PDF)
