# Supplementary material for: A Pyridyl-1,2-azaborine Ligand for Phosphorescent Neutral Iridium(III) Complexes
Source: Inorg Chem. 2023 Jan 25;62(5):2456–69. doi: 10.1021/acs.inorgchem.2c04449 (PMC9906742; doi:10.1021/acs.inorgchem.2c04449)
Supplement: Supplementary file 1 — ic2c04449_si_001.pdf [file ic2c04449_si_001.pdf]

Supporting Information

A Pyridyl-1,2-Azaborine Ligand for Phosphorescent Neutral Iridium(III) Complexes

Andrea Baschieri,*,† Flavia Aleotti,‡ Elia Matteucci,‡ Letizia Sambri,‡ Michele Mancinelli,*,‡ Andrea Mazzanti,‡ Enrico Leoni,†,§ Nicola Armaroli,† and Filippo Monti*,†

† Istituto per la Sintesi Organica e la Fotoreattività, Consiglio Nazionale delle Ricerche, Via P. Gobetti 101, 40129 Bologna, Italy

‡ Dipartimento di Chimica Industriale "Toso Montanari", Università di Bologna, Viale Risorgimento 4, 40136 Bologna, Italy

§ Laboratorio Tecnologie dei Materiali Faenza, ENEA, Via Ravegnana 186, 48018 Faenza (RA), Italy

E-mail: A. B.: andrea.baschieri@isof.cnr.it
M. M.: michele.mancinelli@unibo.it
F. M.: filippo.monti@isof.cnr.it

Table of Contents

Contents	*Pages*
NMR spectra of azaborine ligand **1a**	S2 – S3
NMR spectra of azaborine-based complexes **2a**, **3** and **4**	S4 – S10
NMR spectra of C=C isoelectronic complexes **2b** and **2c**	S11 – S14
X-ray data for complex **2a**	S15 – S23
Computational, electrochemical and photophysical data	S24 – S40

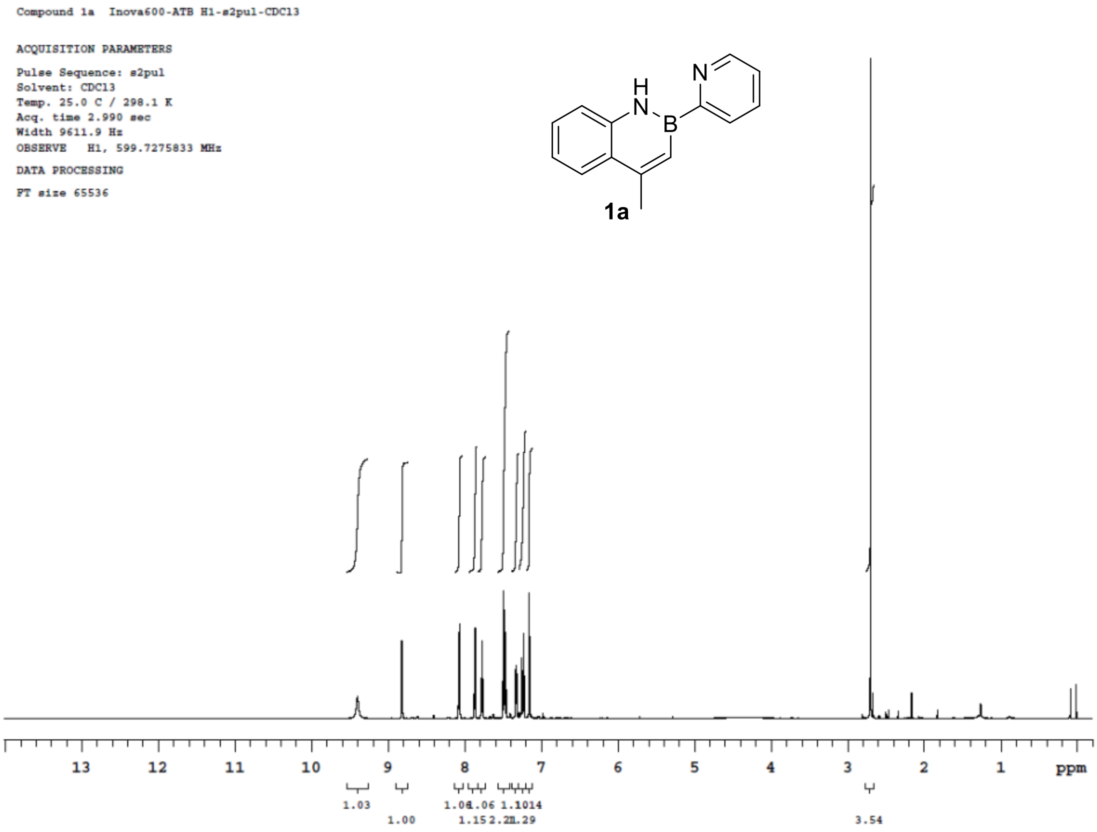

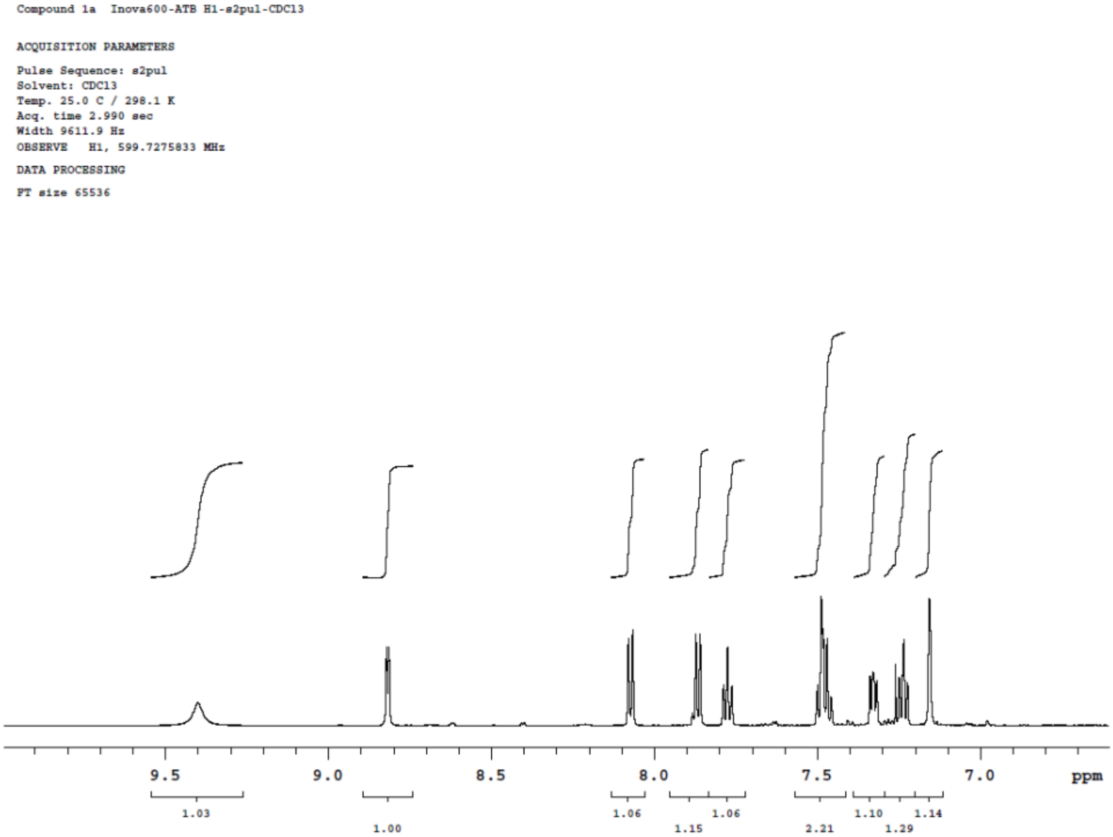

Figure S1. ^{1}H NMR spectrum of azaborine ligand **1a** in $CDCl_3$.

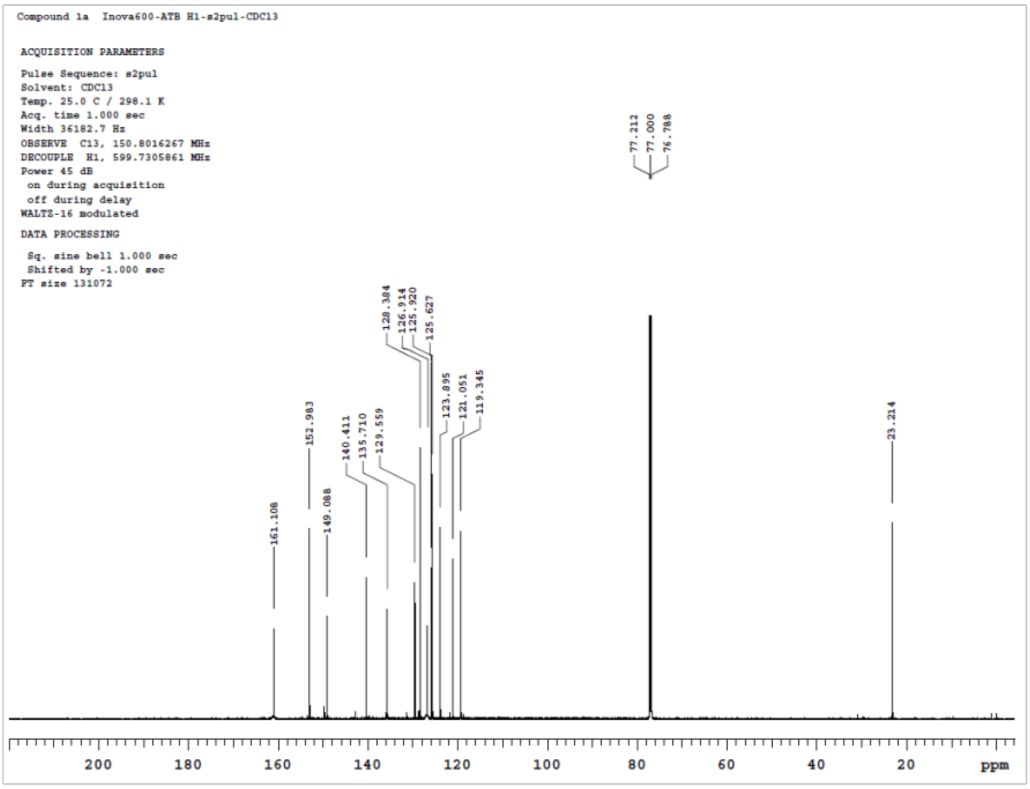

Figure S2. $^{13}C\{^{1}H\}$ NMR spectrum of azaborine ligand **1a** in $CDCl_3$.

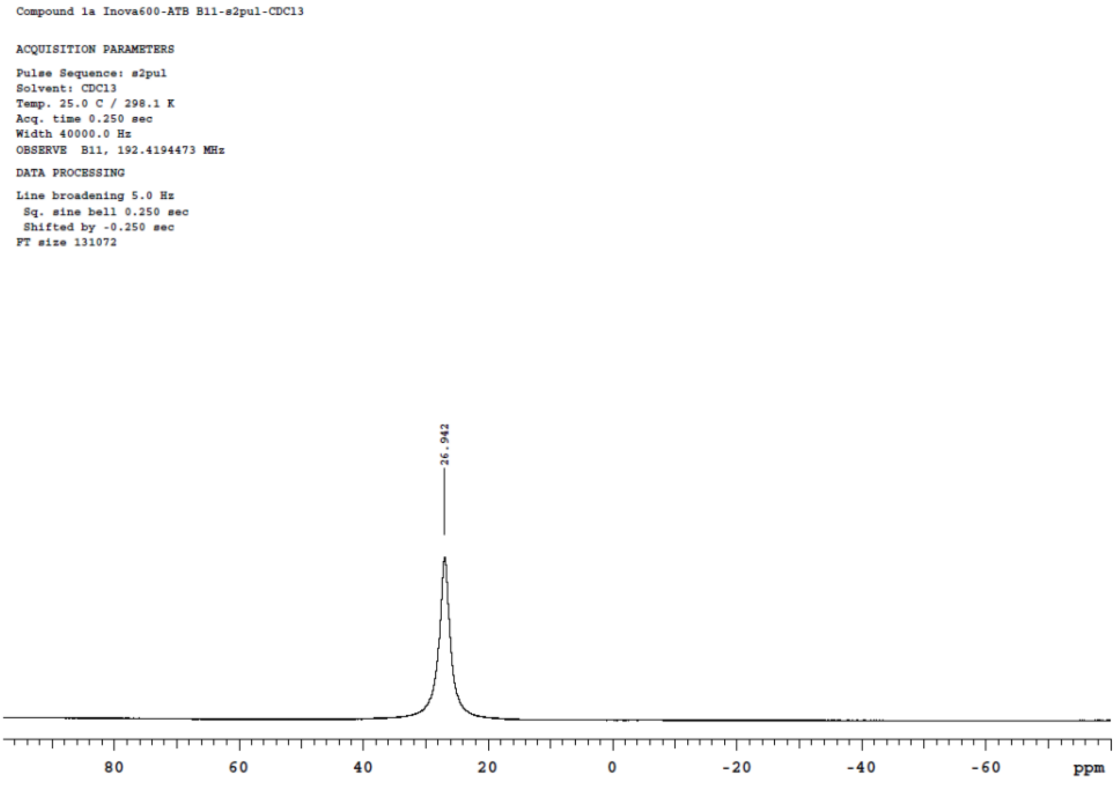

Figure S3. ^{11}B NMR spectrum of azaborine ligand **1a** in $CDCl_3$.

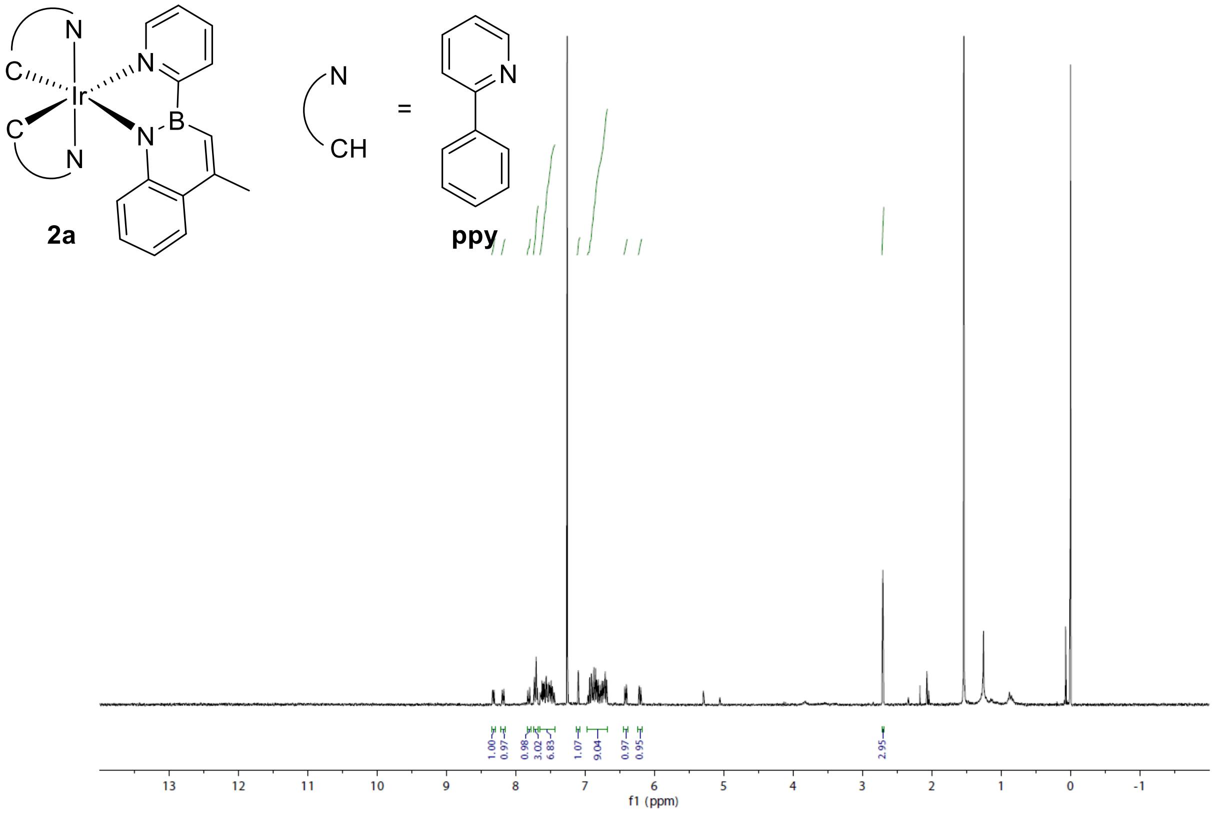

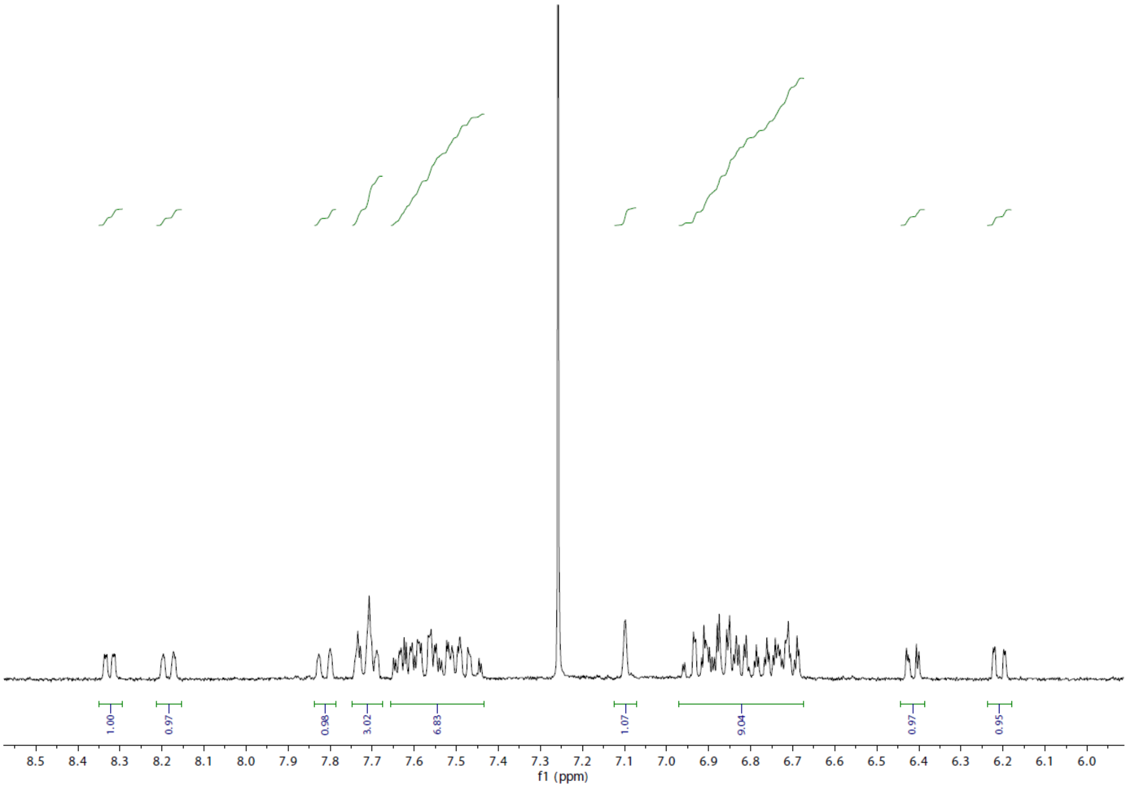

Figure S4. ^{1}H NMR spectrum of complex **2a** in $CDCl_3$.

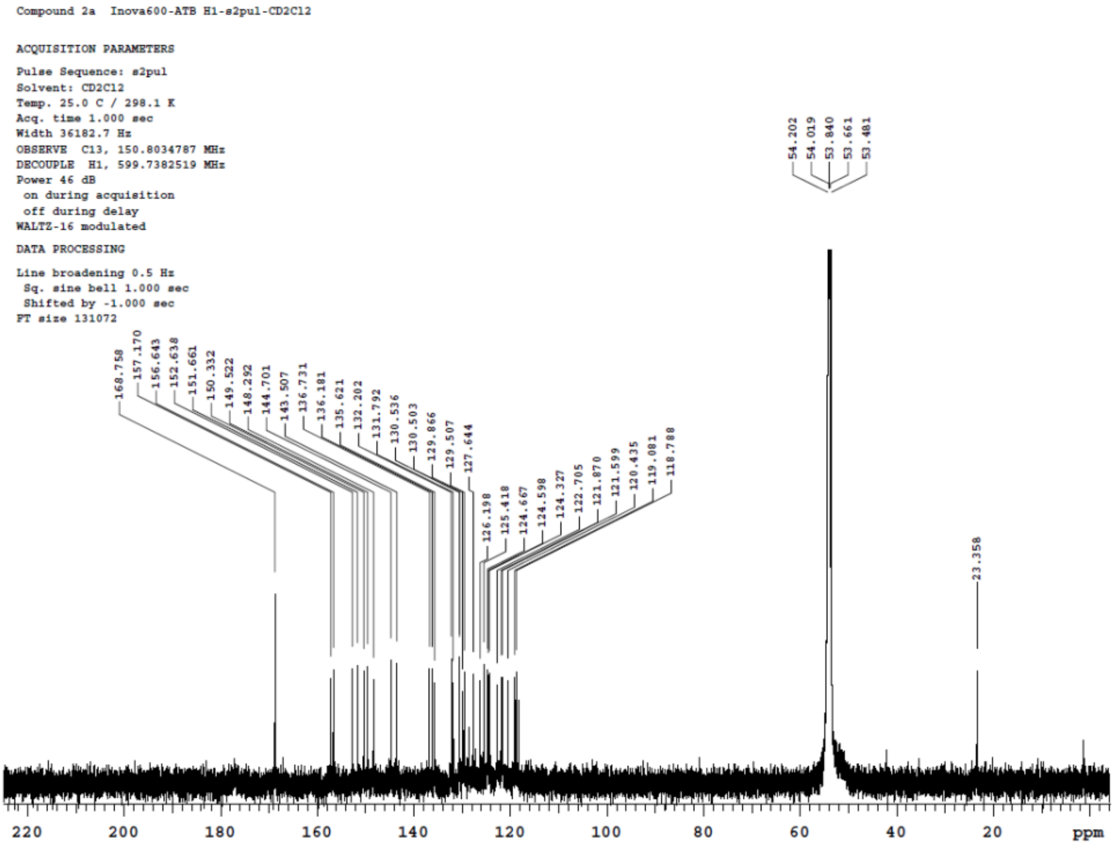

Figure S5. $^{13}C\{^{1}H\}$ NMR spectrum of complex **2a** in CD_2Cl_2.

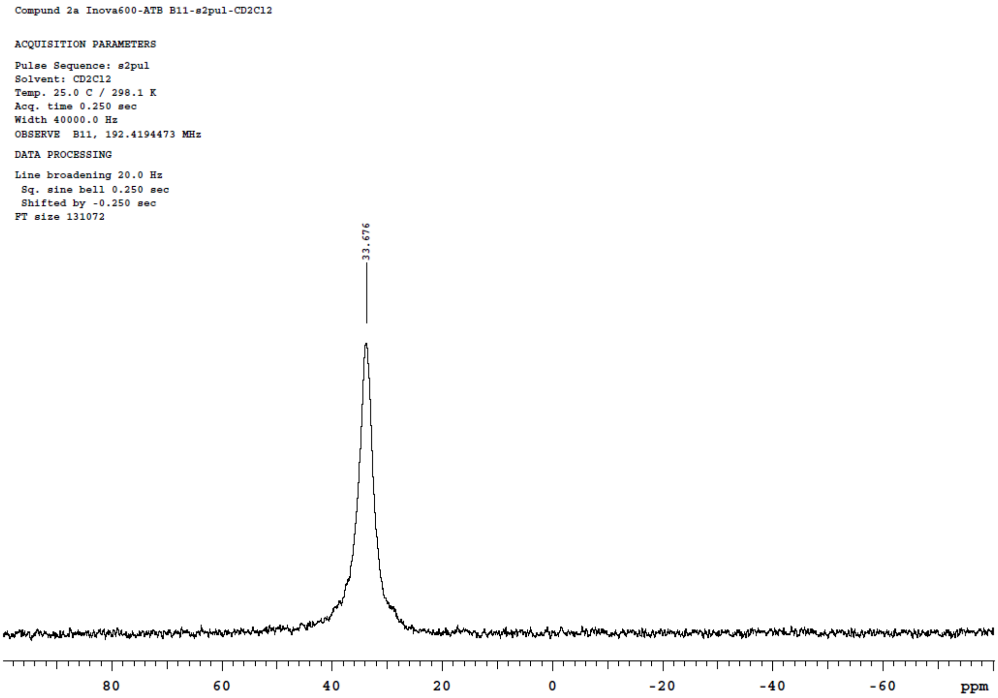

Figure S6. ^{11}B NMR spectrum of complex **2a** in CD_2Cl_2.

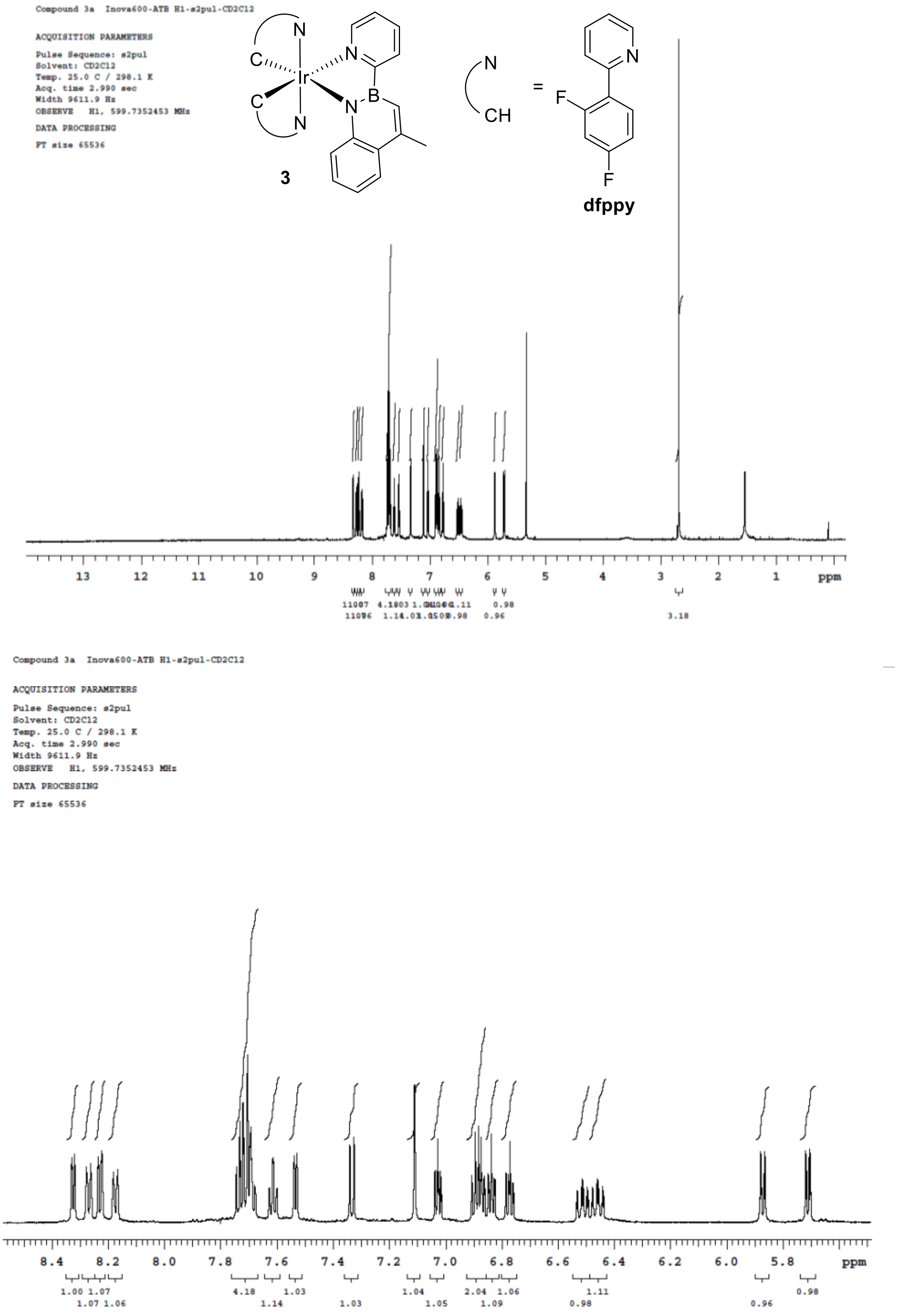

Figure S7. ^{1}H NMR spectrum of complex **3** in CD_2Cl_2.

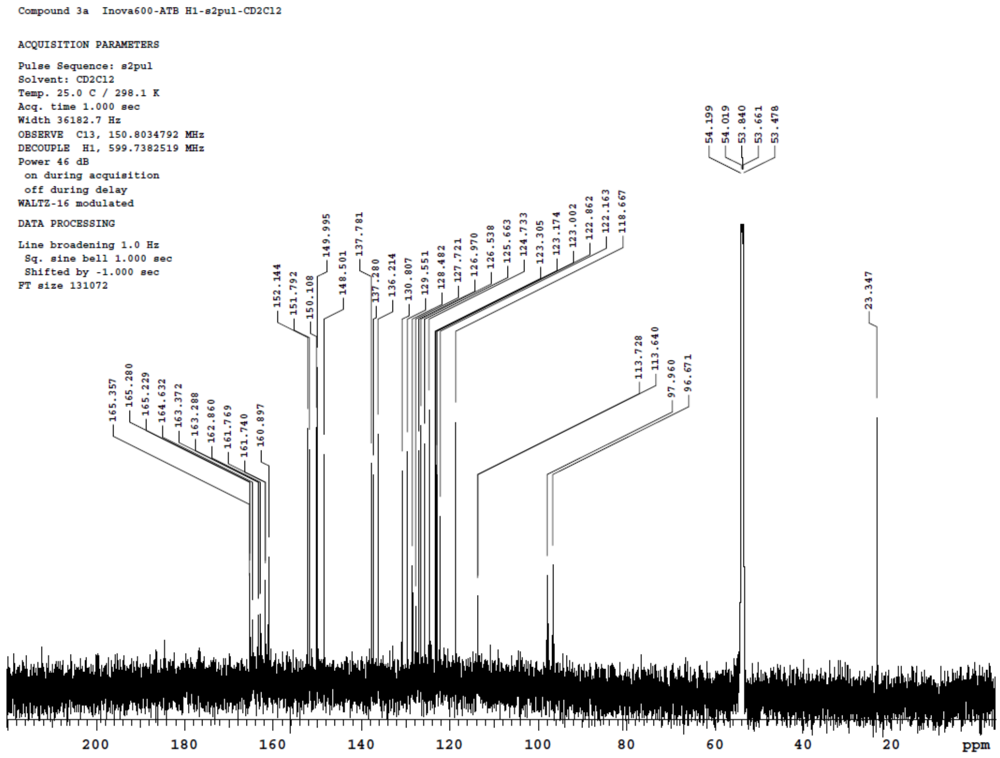

Figure S8. $^{13}C\{^{1}H\}$ NMR spectrum of complex **3** in CD_2Cl_2.

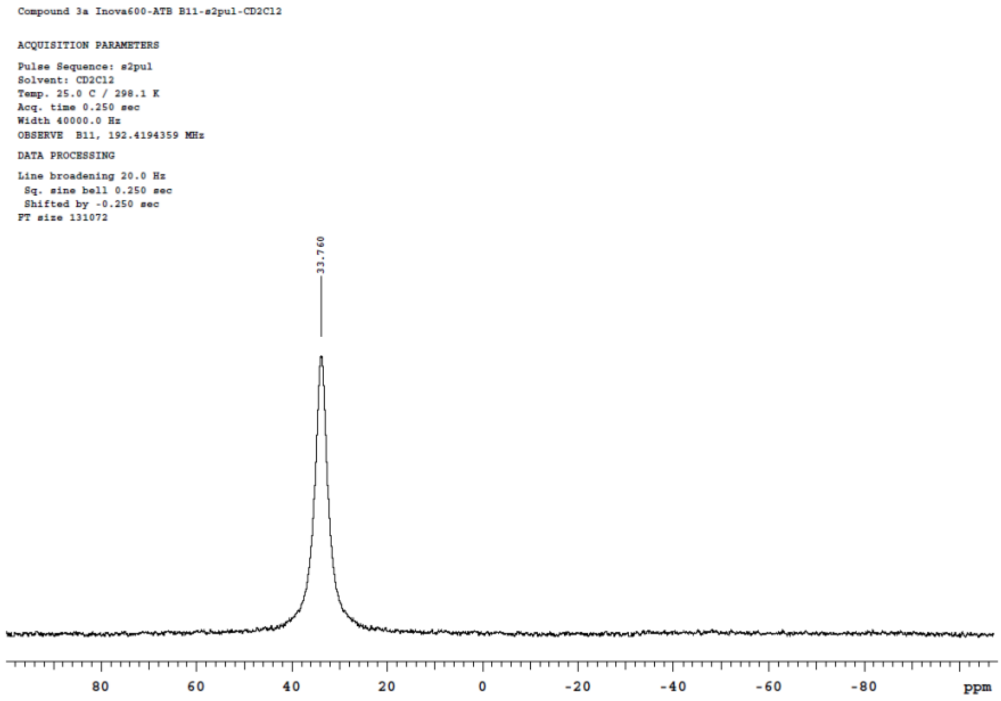

Figure S9. ^{11}B NMR spectrum of complex **3** in CD_2Cl_2.

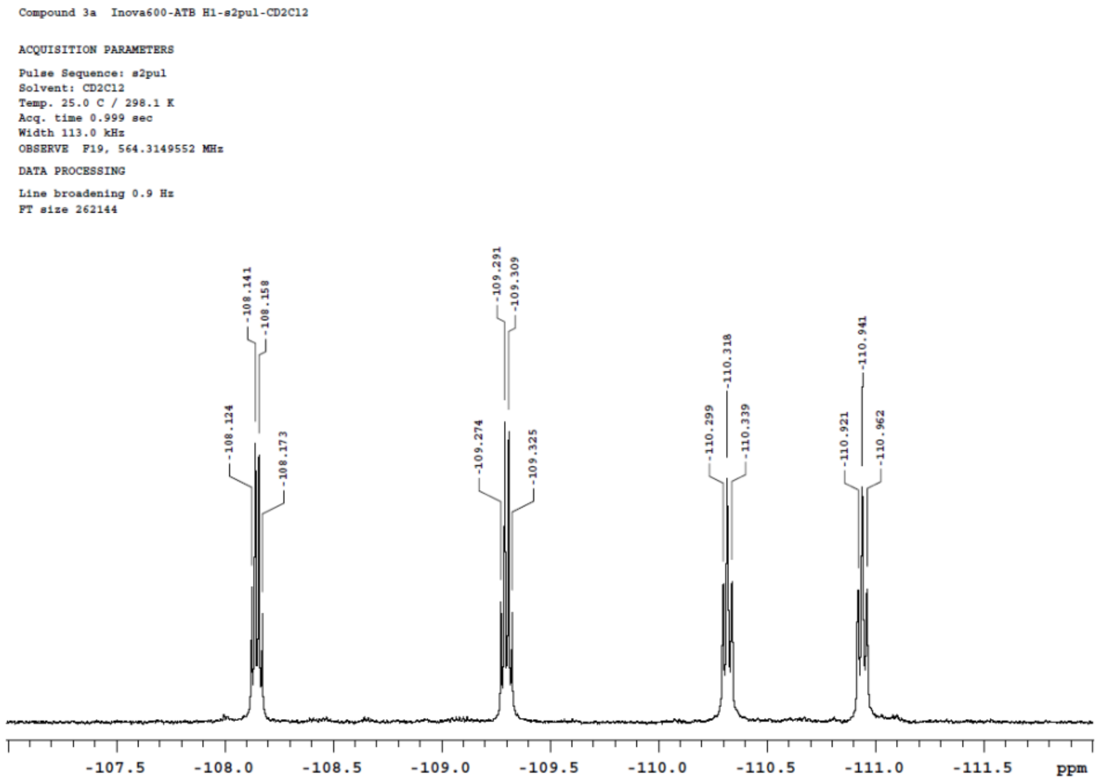

Figure S10. ^{19}F NMR spectrum of complex **3** in CD_2Cl_2.

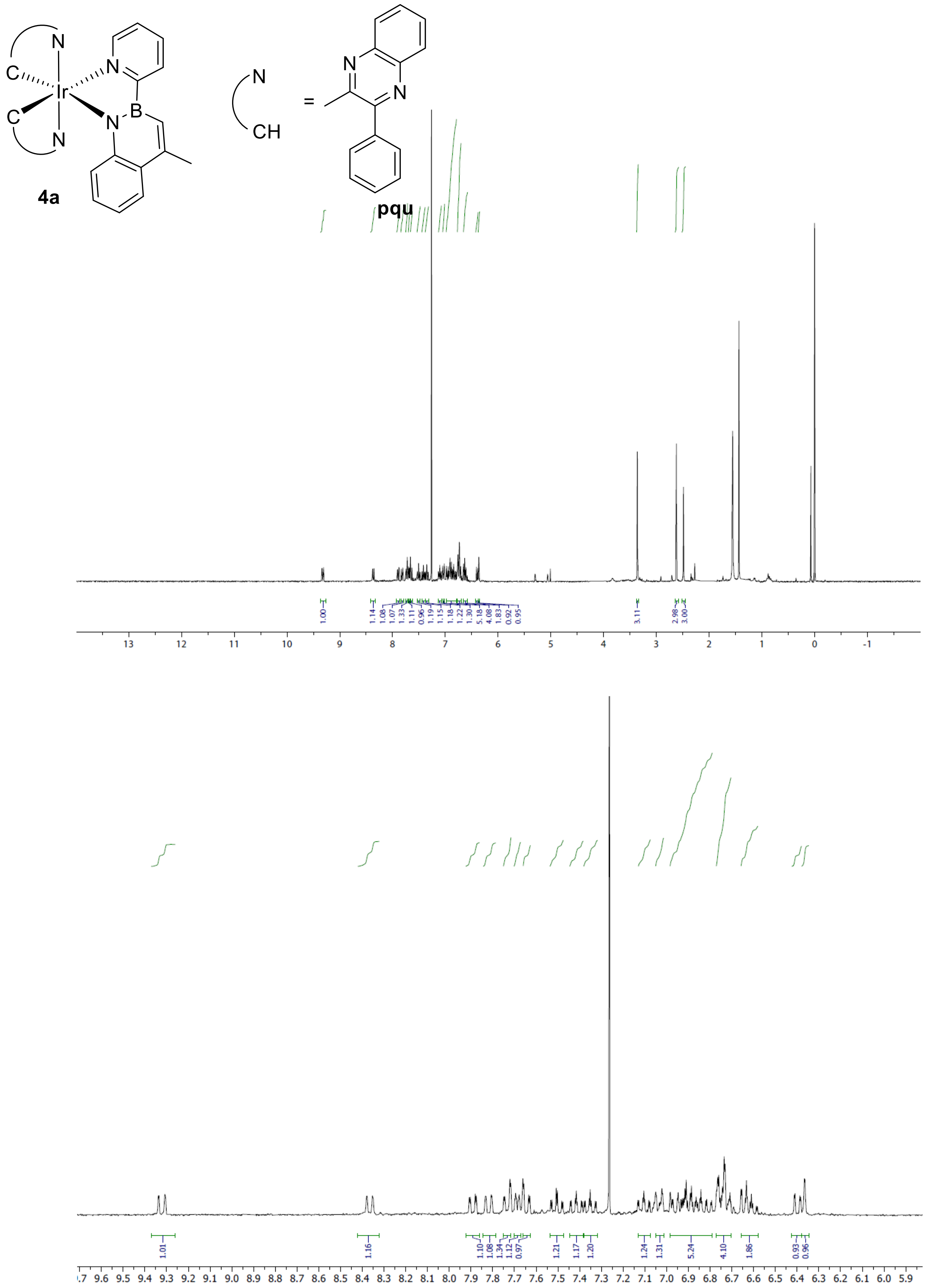

Figure S11. ^{1}H NMR spectrum of complex **4** in $CDCl_3$.

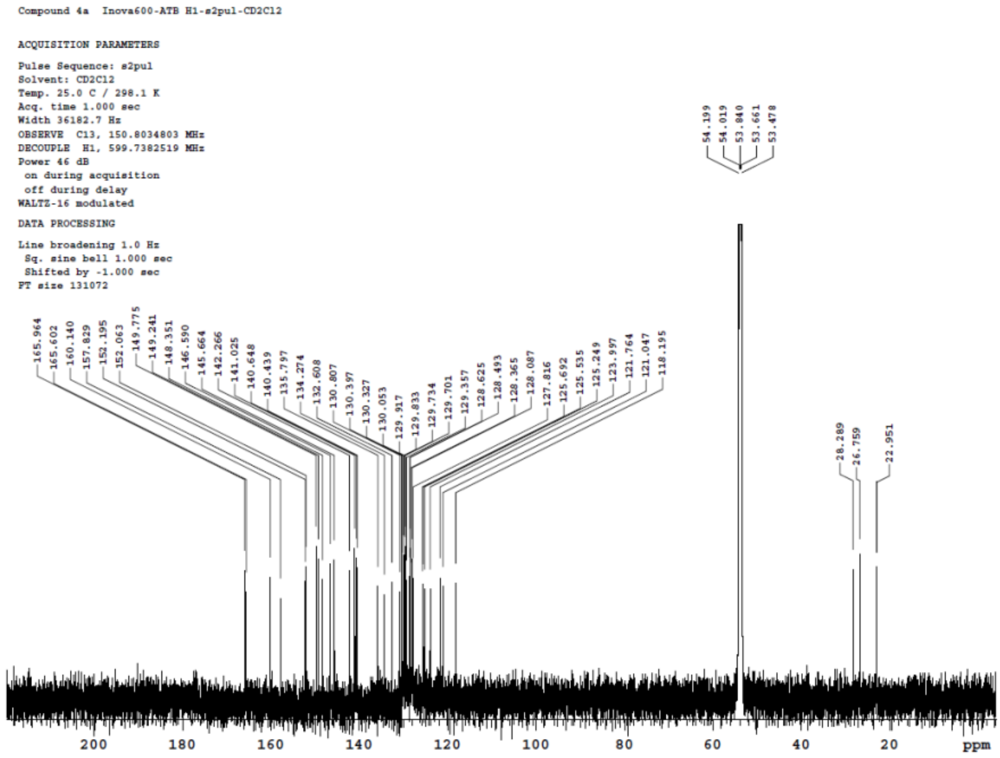

Figure S12. $^{13}C\{^{1}H\}$ NMR spectrum of complex **4** in CD_2Cl_2.

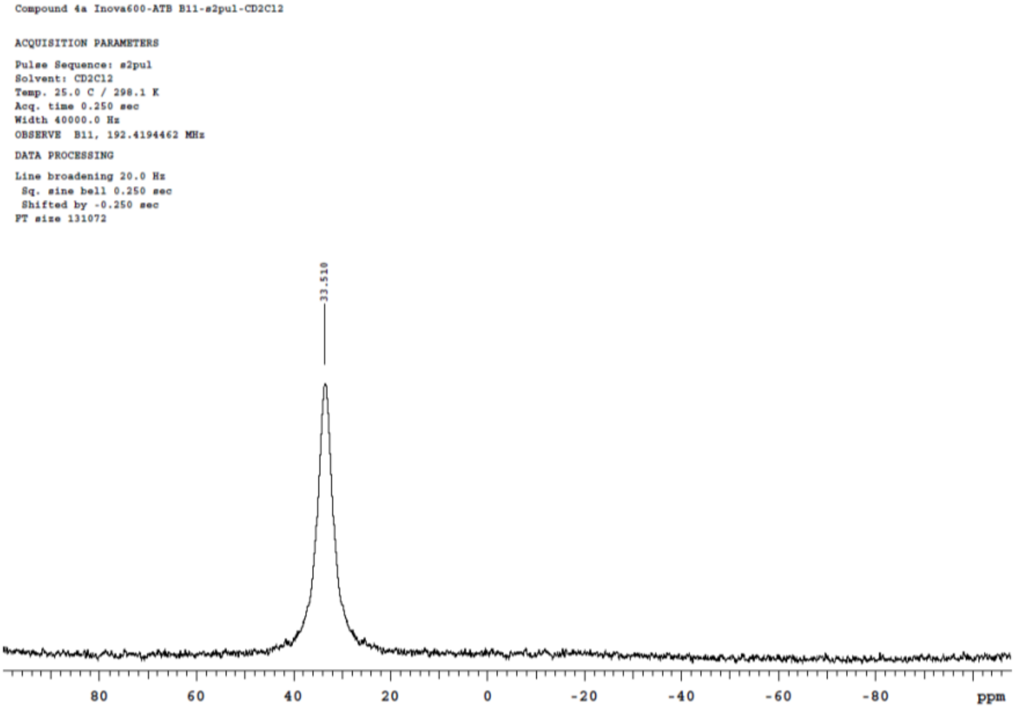

Figure S13. ^{11}B NMR spectrum of complex **4** in CD_2Cl_2.

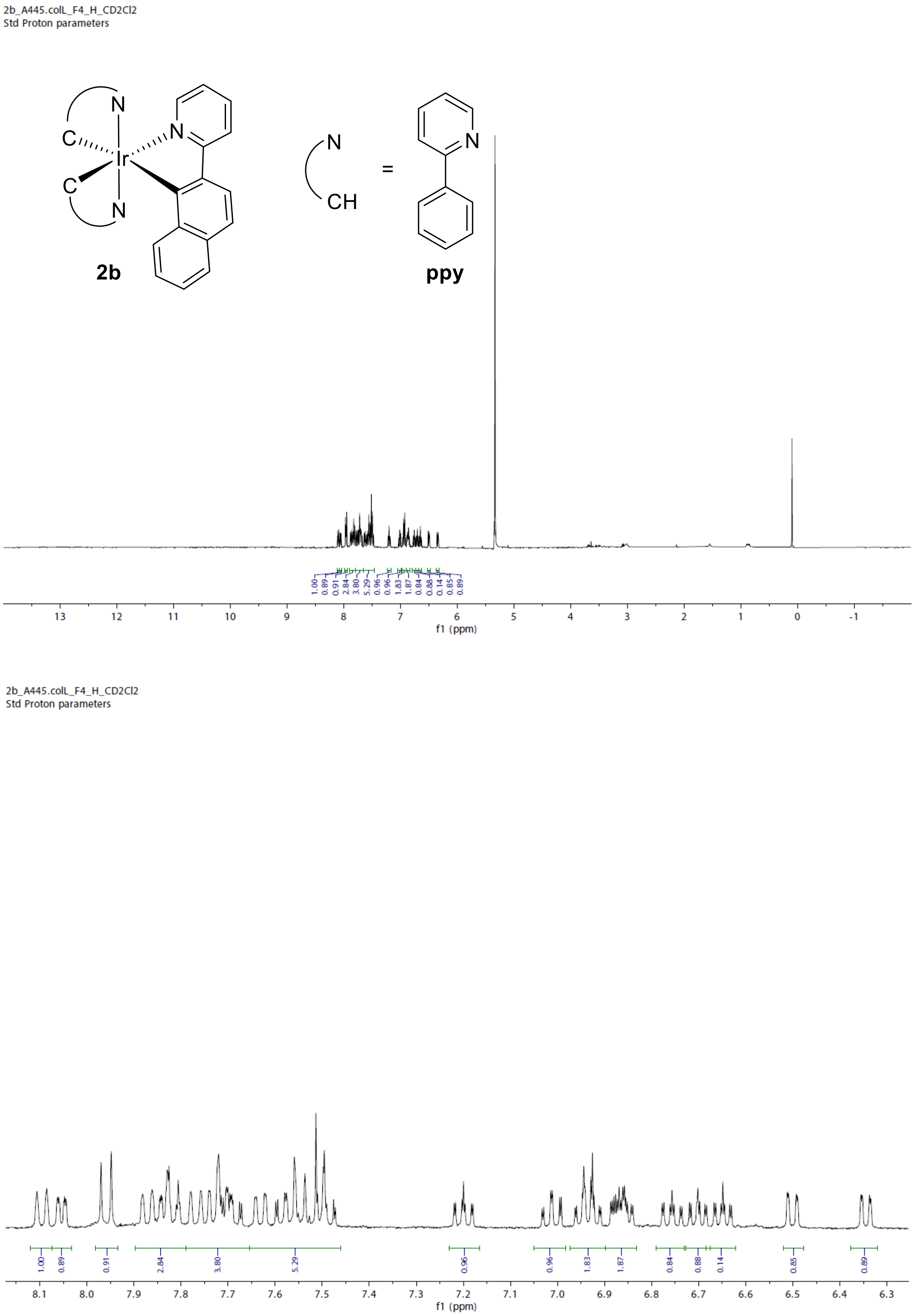

Figure S14. ^{1}H NMR spectrum of complex **2b** in CD_2Cl_2.

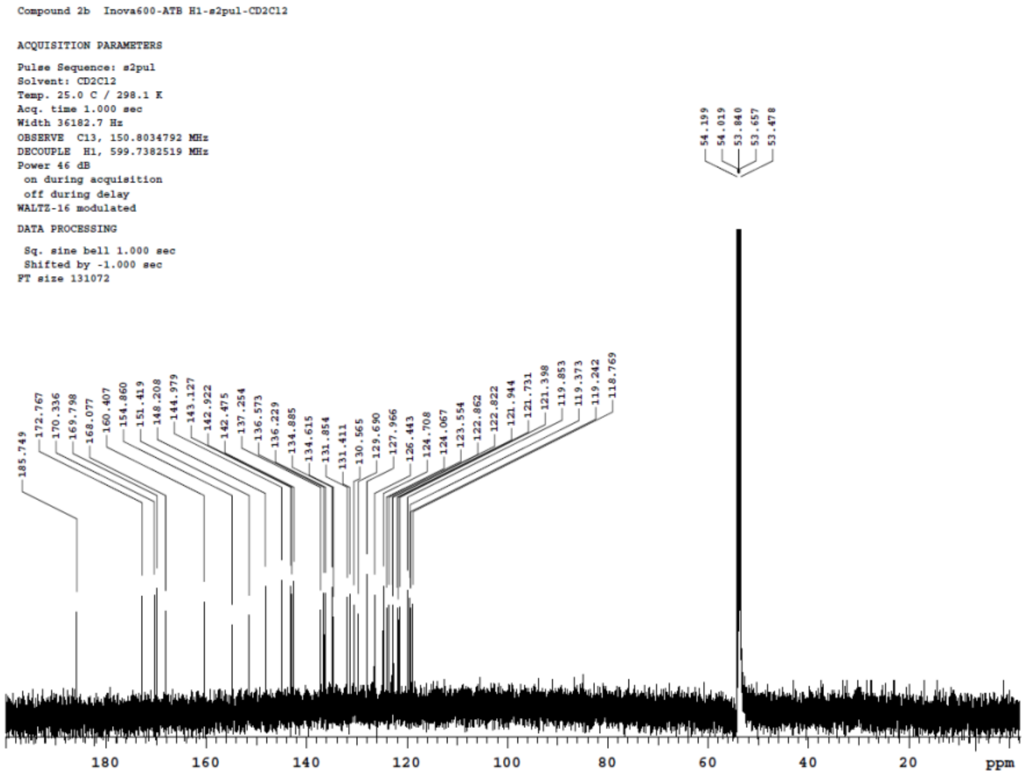

Figure S15. $^{13}C\{^{1}H\}$ NMR spectrum of complex **2b** in CD_2Cl_2.

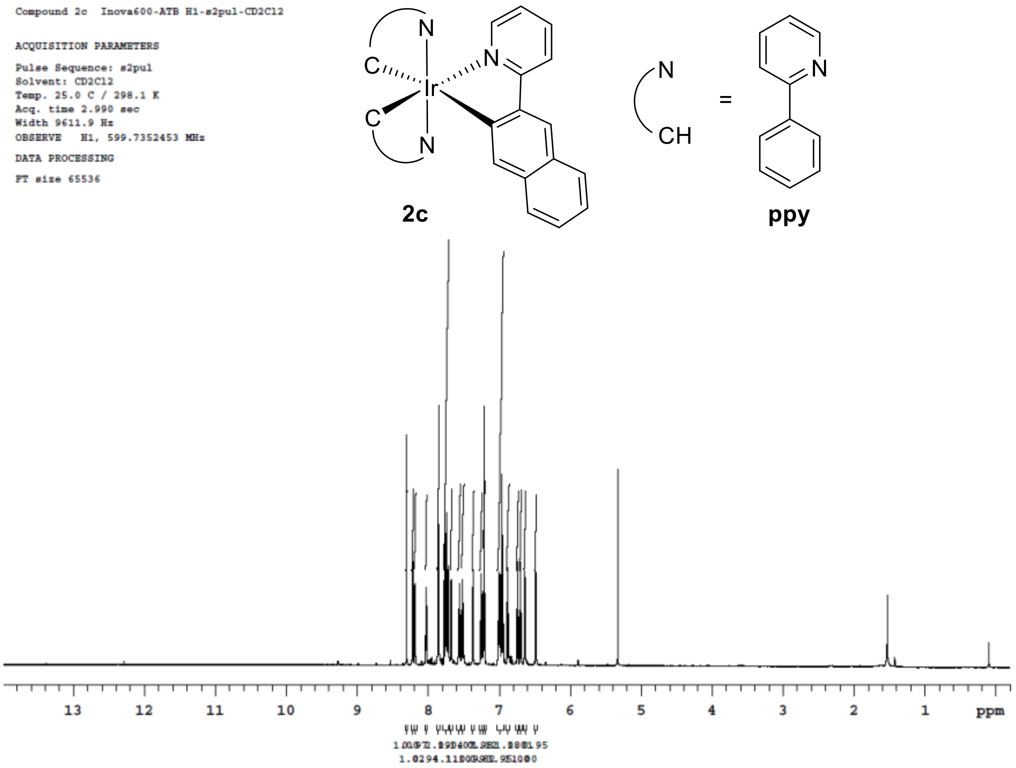

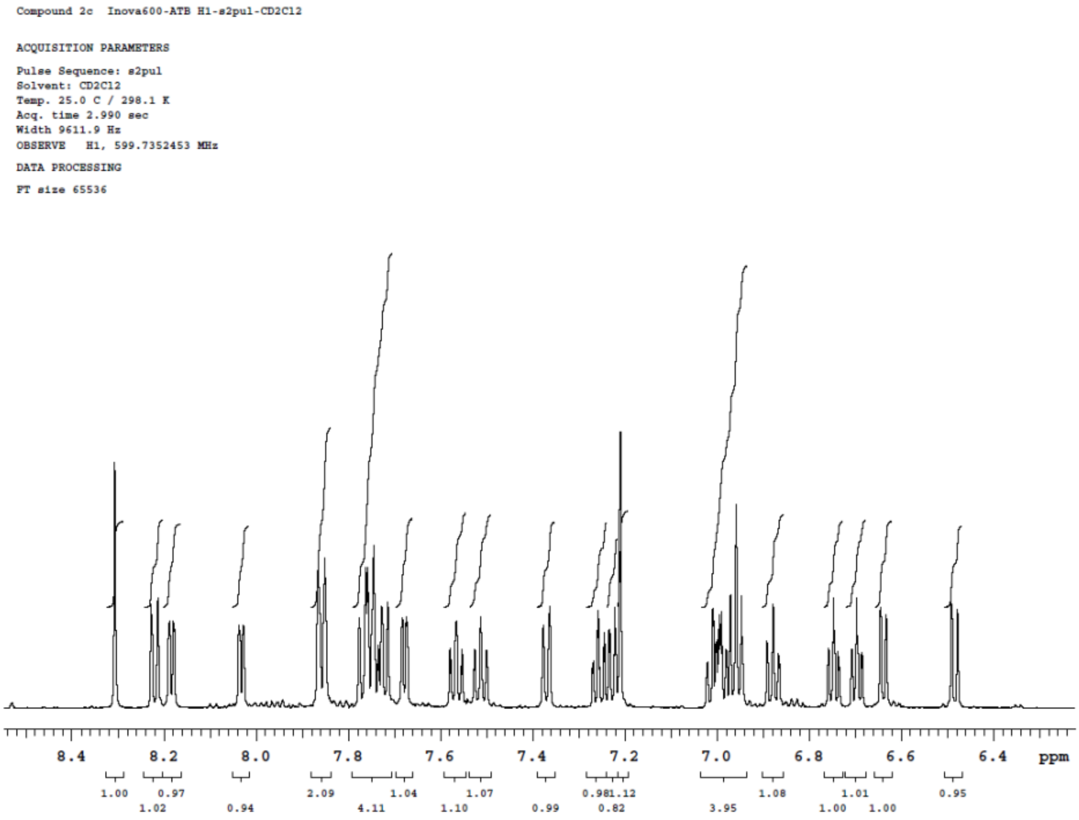

Figure S16. ^{1}H NMR spectrum of complex **2c** in CD_2Cl_2.

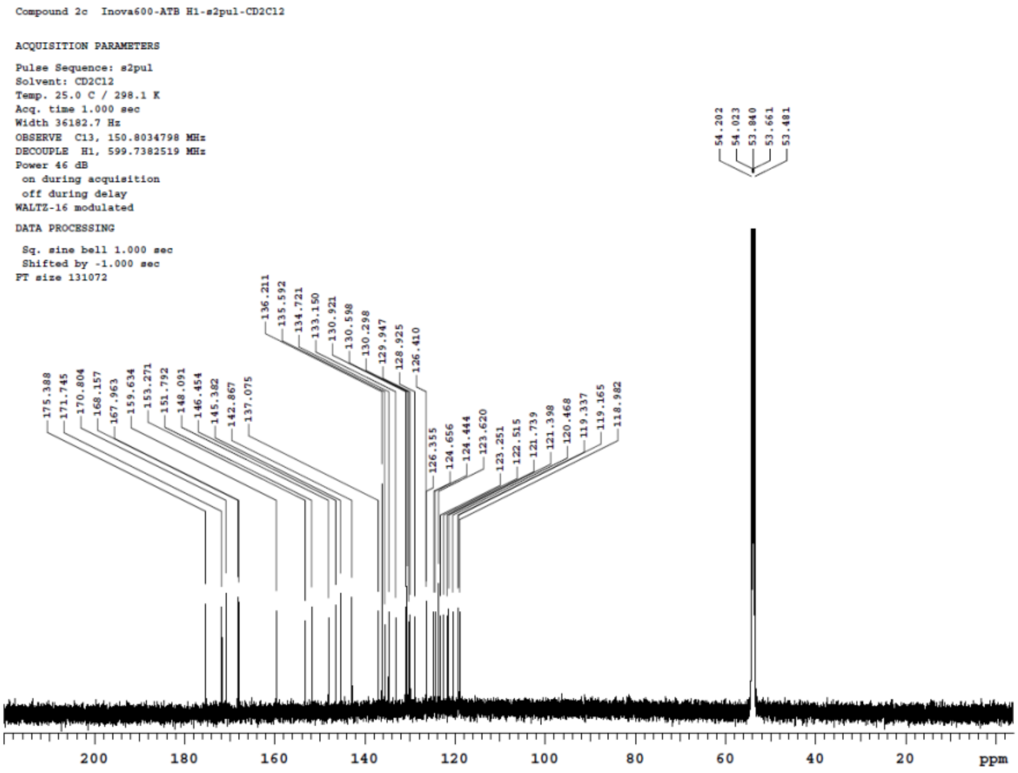

Figure S17. $^{13}C\{^{1}H\}$ NMR spectrum of complex **2c** in CD_2Cl_2.

Crystal Structure Report for complex 2a

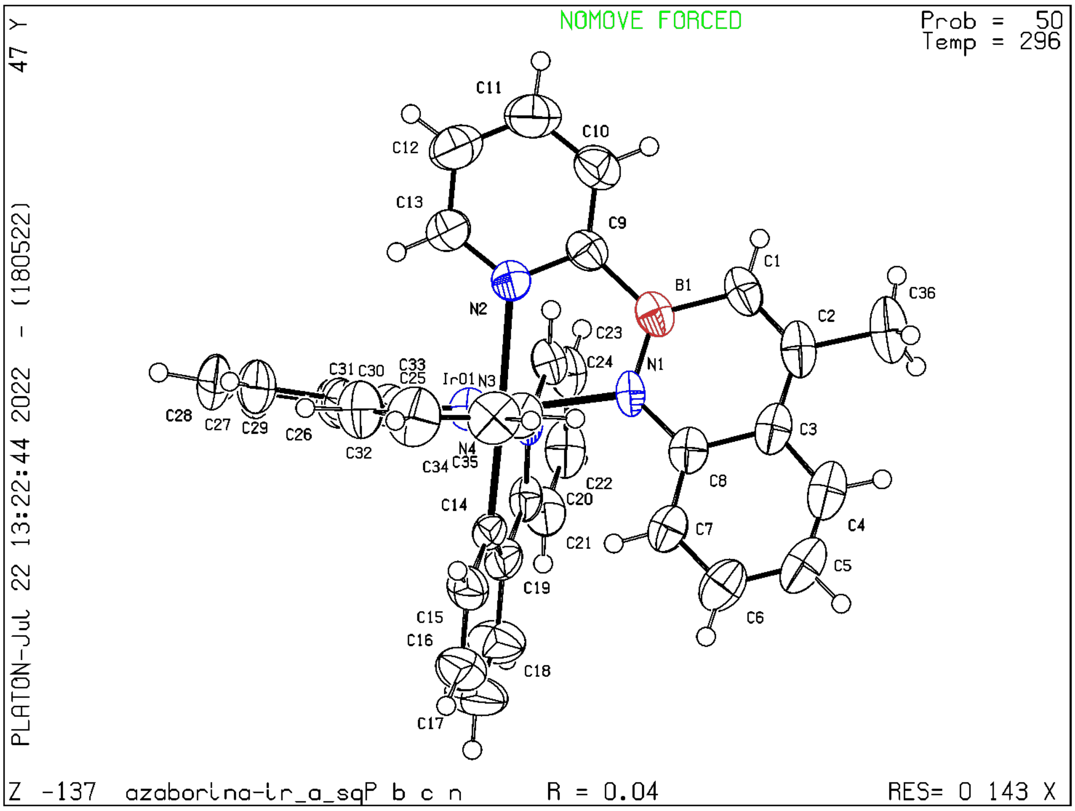

Table S1. Sample and crystal data for compound **2a**.

**Identification code**	leti1702_1	
**Chemical formula**	$C_{36}H_{28}BIrN_4$	
**Formula weight**	719.63 g/mol	
**Temperature**	296(2) K	
**Wavelength**	0.71073 Å	
**Crystal size**	0.100 x 0.100 x 0.200 mm	
**Crystal system**	orthorhombic	
**Space group**	P b c n	
**Unit cell dimensions**	a = 26.512(3) Å	α = 90°
	b = 28.472(3) Å	β = 90°
	c = 10.3066(11) Å	γ = 90°
**Volume**	7779.9(14) Å^3	
**Z**	8	
**Density (calculated)**	1.229 g/cm^3	
**Absorption coefficient**	3.457 mm^{-1}	
**F(000)**	2832	

Table S2. Data collection and structure refinement for compound **2a**.

**Theta range for data collection**	2.24 to 25.00°
**Index ranges**	-31<=h<=31, -33<=k<=33, -12<=l<=12
**Reflections collected**	82938
**Independent reflections**	6851 [R(int) = 0.0476]
**Max. and min. transmission**	0.7240 and 0.5450
**Structure solution technique**	direct methods
**Structure solution program**	SHELXT 2014/5 (Sheldrick, 2014)
**Refinement method**	Full-matrix least-squares on F^2
**Refinement program**	SHELXL-2017/1 (Sheldrick, 2017)
**Function minimized**	$\Sigma w(F_o^2 - F_c^2)^2$
**Data / restraints / parameters**	6851 / 0 / 380
**Goodness-of-fit on F^2**	1.095
**Δ/σ_{max}**	0.001
**Final R indices**	5892 data; I>2σ(I) R1 = 0.0447, wR2 = 0.0976
	all data R1 = 0.0530, wR2 = 0.1020
**Weighting scheme**	$w=1/[\sigma^2(F_o^2)+(0.0363P)^2+37.3090P]$ where $P=(F_o^2+2F_c^2)/3$
**Largest diff. peak and hole**	1.761 and -1.770 eÅ^{-3}
**R.M.S. deviation from mean**	0.096 eÅ^{-3}

Table S3. Atomic coordinates and equivalent isotropic atomic displacement parameters (Å^2) for compound **2a.** U(eq) is defined as one third of the trace of the orthogonalized U_{ij} tensor.

	x/a	y/b	z/c	U(eq)
Ir01	0.66286(2)	0.35525(2)	0.76377(2)	0.03664(9)
N3	0.59602(16)	0.38155(17)	0.8338(4)	0.0347(11)
N2	0.69851(18)	0.42291(17)	0.7397(5)	0.0432(12)
N4	0.72747(17)	0.32351(18)	0.7049(5)	0.0407(11)
N1	0.63785(18)	0.37485(18)	0.5692(4)	0.0399(11)
C25	0.6963(2)	0.3453(2)	0.9354(5)	0.0403(14)
C14	0.6253(2)	0.2948(2)	0.8012(6)	0.0390(13)
C7	0.6059(2)	0.3000(2)	0.4927(6)	0.0494(16)
C8	0.6123(2)	0.3482(2)	0.4777(6)	0.0443(15)
C20	0.5607(2)	0.3493(2)	0.8690(5)	0.0415(14)
C24	0.5848(2)	0.4271(2)	0.8434(5)	0.0423(14)
C1	0.6267(3)	0.4448(3)	0.4192(6)	0.0549(17)
C3	0.5933(2)	0.3694(3)	0.3593(6)	0.0498(16)
C23	0.5394(2)	0.4433(3)	0.8885(6)	0.0530(17)
C30	0.7449(2)	0.3260(2)	0.9293(6)	0.0444(15)
C19	0.5771(2)	0.3010(2)	0.8524(6)	0.0478(15)
C21	0.5142(2)	0.3644(3)	0.9150(7)	0.0550(18)
C26	0.6788(3)	0.3581(2)	0.0589(7)	0.0546(17)

	x/a	y/b	z/c	U(eq)
C9	0.6819(2)	0.4490(2)	0.6380(6)	0.0453(15)
C35	0.7379(2)	0.3117(2)	0.5821(6)	0.0505(16)
C31	0.7610(2)	0.3114(2)	0.7998(7)	0.0460(15)
C22	0.5042(2)	0.4107(3)	0.9241(6)	0.0585(19)
C34	0.7818(3)	0.2879(3)	0.5487(8)	0.063(2)
C2	0.5997(2)	0.4183(3)	0.3362(6)	0.0549(18)
C29	0.7751(3)	0.3214(3)	0.0382(7)	0.063(2)
C15	0.6423(3)	0.2490(2)	0.7842(7)	0.0534(16)
C27	0.7087(3)	0.3532(3)	0.1677(6)	0.063(2)
B1	0.6473(3)	0.4221(3)	0.5418(7)	0.0452(17)
C6	0.5828(3)	0.2724(3)	0.4031(7)	0.065(2)
C33	0.8145(3)	0.2746(3)	0.6427(9)	0.074(2)
C13	0.7317(3)	0.4415(2)	0.8225(7)	0.0564(17)
C16	0.6121(3)	0.2109(3)	0.8160(9)	0.075(2)
C28	0.7573(3)	0.3355(3)	0.1570(7)	0.067(2)
C10	0.6994(3)	0.4948(3)	0.6245(7)	0.0605(18)
C4	0.5688(3)	0.3390(3)	0.2719(7)	0.066(2)
C32	0.8045(3)	0.2866(3)	0.7691(7)	0.065(2)
C18	0.5473(3)	0.2618(3)	0.8832(9)	0.074(2)
C5	0.5641(3)	0.2925(3)	0.2918(8)	0.071(2)
C12	0.7508(3)	0.4865(3)	0.8109(9)	0.075(2)
C11	0.7331(3)	0.5138(3)	0.7101(9)	0.078(2)
C36	0.5767(3)	0.4400(3)	0.2137(7)	0.078(2)
C17	0.5642(3)	0.2179(3)	0.8651(10)	0.086(3)

Table S4. Bond lengths (Å) for compound **2a**.

Ir01-C25	1.999(5)	Ir01-C14	2.025(6)
Ir01-N4	2.030(4)	Ir01-N3	2.055(4)
Ir01-N2	2.160(5)	Ir01-N1	2.184(4)
N3-C24	1.336(7)	N3-C20	1.359(7)
N2-C13	1.335(8)	N2-C9	1.357(8)
N4-C35	1.339(8)	N4-C31	1.366(8)
N1-C8	1.387(8)	N1-B1	1.398(9)
C25-C26	1.402(9)	C25-C30	1.404(8)
C14-C15	1.390(9)	C14-C19	1.394(8)
C7-C6	1.357(9)	C7-C8	1.393(9)
C7-H7	0.93	C8-C3	1.452(9)
C20-C21	1.389(8)	C20-C19	1.452(9)
C24-C23	1.371(8)	C24-H24	0.93
C1-C2	1.346(10)	C1-B1	1.521(9)

C1-H1	0.93	C3-C4	1.407(10)
C3-C2	1.424(10)	C23-C22	1.366(10)
C23-H23	0.93	C30-C29	1.385(9)
C30-C31	1.461(9)	C19-C18	1.404(9)
C21-C22	1.347(10)	C21-H21	0.93
C26-C27	1.379(9)	C26-H26	0.93
C9-C10	1.392(9)	C9-B1	1.552(10)
C35-C34	1.388(9)	C35-H35	0.93
C31-C32	1.388(9)	C22-H22	0.93
C34-C33	1.353(11)	C34-H34	0.93
C2-C36	1.532(9)	C29-C28	1.373(10)
C29-H29	0.93	C15-C16	1.387(10)
C15-H15	0.93	C27-C28	1.389(10)
C27-H27	0.93	C6-C5	1.373(11)
C6-H6	0.93	C33-C32	1.372(11)
C33-H33	0.93	C13-C12	1.384(10)
C13-H13	0.93	C16-C17	1.381(12)
C16-H16	0.93	C28-H28	0.93
C10-C11	1.367(11)	C10-H10	0.93
C4-C5	1.346(12)	C4-H4	0.93
C32-H32	0.93	C18-C17	1.341(11)
C18-H18	0.93	C5-H5	0.93
C12-C11	1.379(12)	C12-H12	0.93
C11-H11	0.93	C36-H36A	0.96
C36-H36B	0.96	C36-H36C	0.96
C17-H17	0.93		

Table S5. Bond angles (°) for compound **2a**.

C25-Ir01-C14	86.0(2)	C25-Ir01-N4	80.1(2)
C14-Ir01-N4	95.4(2)	C25-Ir01-N3	97.1(2)
C14-Ir01-N3	79.6(2)	N4-Ir01-N3	174.38(19)
C25-Ir01-N2	91.9(2)	C14-Ir01-N2	174.2(2)
N4-Ir01-N2	89.62(19)	N3-Ir01-N2	95.32(19)
C25-Ir01-N1	169.5(2)	C14-Ir01-N1	104.0(2)
N4-Ir01-N1	95.48(19)	N3-Ir01-N1	88.15(17)
N2-Ir01-N1	78.43(19)	C24-N3-C20	118.9(5)
C24-N3-Ir01	124.9(4)	C20-N3-Ir01	116.2(4)
C13-N2-C9	119.4(6)	C13-N2-Ir01	124.6(4)
C9-N2-Ir01	115.8(4)	C35-N4-C31	118.6(5)
C35-N4-Ir01	124.7(4)	C31-N4-Ir01	116.6(4)

C8-N1-B1	118.5(5)	C8-N1-Ir01	129.3(4)
B1-N1-Ir01	112.1(4)	C26-C25-C30	116.4(5)
C26-C25-Ir01	128.4(5)	C30-C25-Ir01	115.0(4)
C15-C14-C19	117.6(6)	C15-C14-Ir01	127.9(5)
C19-C14-Ir01	114.5(5)	C6-C7-C8	123.3(7)
C6-C7-H7	118.3	C8-C7-H7	118.3
N1-C8-C7	121.6(6)	N1-C8-C3	121.0(6)
C7-C8-C3	117.4(6)	N3-C20-C21	119.6(6)
N3-C20-C19	113.7(5)	C21-C20-C19	126.7(6)
N3-C24-C23	123.1(6)	N3-C24-H24	118.4
C23-C24-H24	118.4	C2-C1-B1	118.8(7)
C2-C1-H1	120.6	B1-C1-H1	120.6
C4-C3-C2	123.4(7)	C4-C3-C8	116.3(7)
C2-C3-C8	120.3(6)	C22-C23-C24	117.7(7)
C22-C23-H23	121.2	C24-C23-H23	121.2
C29-C30-C25	122.1(6)	C29-C30-C31	123.0(6)
C25-C30-C31	114.9(5)	C14-C19-C18	120.0(6)
C14-C19-C20	116.0(5)	C18-C19-C20	124.0(6)
C22-C21-C20	120.1(7)	C22-C21-H21	119.9
C20-C21-H21	119.9	C27-C26-C25	121.6(6)
C27-C26-H26	119.2	C25-C26-H26	119.2
N2-C9-C10	118.8(6)	N2-C9-B1	114.6(5)
C10-C9-B1	126.5(6)	N4-C35-C34	122.0(6)
N4-C35-H35	119.0	C34-C35-H35	119.0
N4-C31-C32	120.4(6)	N4-C31-C30	113.1(5)
C32-C31-C30	126.5(6)	C21-C22-C23	120.7(6)
C21-C22-H22	119.7	C23-C22-H22	119.7
C33-C34-C35	119.7(7)	C33-C34-H34	120.2
C35-C34-H34	120.2	C1-C2-C3	120.4(6)
C1-C2-C36	120.7(7)	C3-C2-C36	118.9(7)
C28-C29-C30	119.8(7)	C28-C29-H29	120.1
C30-C29-H29	120.1	C16-C15-C14	121.2(7)
C16-C15-H15	119.4	C14-C15-H15	119.4
C26-C27-C28	120.2(7)	C26-C27-H27	119.9
C28-C27-H27	119.9	N1-B1-C1	120.8(6)
N1-B1-C9	116.8(6)	C1-B1-C9	122.3(6)
C7-C6-C5	119.4(8)	C7-C6-H6	120.3
C5-C6-H6	120.3	C34-C33-C32	119.1(7)
C34-C33-H33	120.5	C32-C33-H33	120.5
N2-C13-C12	123.5(7)	N2-C13-H13	118.2
C12-C13-H13	118.2	C17-C16-C15	120.2(7)
C17-C16-H16	119.9	C15-C16-H16	119.9

C29-C28-C27	119.8(6)	C29-C28-H28	120.1
C27-C28-H28	120.1	C11-C10-C9	121.5(7)
C11-C10-H10	119.2	C9-C10-H10	119.2
C5-C4-C3	123.4(7)	C5-C4-H4	118.3
C3-C4-H4	118.3	C33-C32-C31	120.2(7)
C33-C32-H32	119.9	C31-C32-H32	119.9
C17-C18-C19	121.5(7)	C17-C18-H18	119.3
C19-C18-H18	119.3	C4-C5-C6	120.2(7)
C4-C5-H5	119.9	C6-C5-H5	119.9
C11-C12-C13	117.5(8)	C11-C12-H12	121.2
C13-C12-H12	121.2	C10-C11-C12	119.1(7)
C10-C11-H11	120.4	C12-C11-H11	120.4
C2-C36-H36A	109.5	C2-C36-H36B	109.5
H36A-C36-H36B	109.5	C2-C36-H36C	109.5
H36A-C36-H36C	109.5	H36B-C36-H36C	109.5
C18-C17-C16	119.5(8)	C18-C17-H17	120.2
C16-C17-H17	120.2		

Table S6. Torsional angles (°) for compound **2a**.

B1-N1-C8-C7	173.7(6)	Ir01-N1-C8-C7	-10.7(8)
B1-N1-C8-C3	-3.5(8)	Ir01-N1-C8-C3	172.1(4)
C6-C7-C8-N1	-177.4(6)	C6-C7-C8-C3	-0.1(9)
C24-N3-C20-C21	-0.6(8)	Ir01-N3-C20-C21	-178.8(4)
C24-N3-C20-C19	179.1(5)	Ir01-N3-C20-C19	0.9(6)
C20-N3-C24-C23	0.7(8)	Ir01-N3-C24-C23	178.7(4)
N1-C8-C3-C4	178.8(6)	C7-C8-C3-C4	1.5(8)
N1-C8-C3-C2	-1.2(9)	C7-C8-C3-C2	-178.5(6)
N3-C24-C23-C22	-0.4(9)	C26-C25-C30-C29	-2.6(10)
Ir01-C25-C30-C29	173.2(5)	C26-C25-C30-C31	177.6(6)
Ir01-C25-C30-C31	-6.6(7)	C15-C14-C19-C18	-0.9(10)
Ir01-C14-C19-C18	-179.7(6)	C15-C14-C19-C20	179.8(6)
Ir01-C14-C19-C20	1.1(7)	N3-C20-C19-C14	-1.3(8)
C21-C20-C19-C14	178.4(6)	N3-C20-C19-C18	179.5(6)
C21-C20-C19-C18	-0.8(11)	N3-C20-C21-C22	0.3(9)
C19-C20-C21-C22	-179.4(6)	C30-C25-C26-C27	2.1(10)
Ir01-C25-C26-C27	-173.1(5)	C13-N2-C9-C10	-1.2(9)
Ir01-N2-C9-C10	173.4(5)	C13-N2-C9-B1	174.6(6)
Ir01-N2-C9-B1	-10.7(6)	C31-N4-C35-C34	-0.5(10)
Ir01-N4-C35-C34	-176.8(5)	C35-N4-C31-C32	-1.1(9)
Ir01-N4-C31-C32	175.4(5)	C35-N4-C31-C30	-179.5(6)

Ir01-N4-C31-C30	-3.0(7)	C29-C30-C31-N4	-173.6(6)
C25-C30-C31-N4	6.2(8)	C29-C30-C31-C32	8.1(11)
C25-C30-C31-C32	-172.1(7)	C20-C21-C22-C23	0.0(10)
C24-C23-C22-C21	0.1(10)	N4-C35-C34-C33	2.5(12)
B1-C1-C2-C3	-3.7(9)	B1-C1-C2-C36	178.0(6)
C4-C3-C2-C1	-175.0(6)	C8-C3-C2-C1	4.9(9)
C4-C3-C2-C36	3.2(10)	C8-C3-C2-C36	-176.8(6)
C25-C30-C29-C28	0.8(11)	C31-C30-C29-C28	-179.4(7)
C19-C14-C15-C16	0.6(10)	Ir01-C14-C15-C16	179.2(6)
C25-C26-C27-C28	0.1(12)	C8-N1-B1-C1	4.6(9)
Ir01-N1-B1-C1	-171.7(5)	C8-N1-B1-C9	-172.2(5)
Ir01-N1-B1-C9	11.4(7)	C2-C1-B1-N1	-1.0(9)
C2-C1-B1-C9	175.6(6)	N2-C9-B1-N1	-0.8(8)
C10-C9-B1-N1	174.7(6)	N2-C9-B1-C1	-177.6(6)
C10-C9-B1-C1	-2.1(10)	C8-C7-C6-C5	-0.8(11)
C35-C34-C33-C32	-2.7(13)	C9-N2-C13-C12	-0.1(10)
Ir01-N2-C13-C12	-174.2(6)	C14-C15-C16-C17	0.2(12)
C30-C29-C28-C27	1.5(12)	C26-C27-C28-C29	-2.0(12)
N2-C9-C10-C11	0.8(10)	B1-C9-C10-C11	-174.6(7)
C2-C3-C4-C5	177.9(7)	C8-C3-C4-C5	-2.1(10)
C34-C33-C32-C31	1.1(13)	N4-C31-C32-C33	0.9(11)
C30-C31-C32-C33	179.1(7)	C14-C19-C18-C17	0.4(13)
C20-C19-C18-C17	179.6(8)	C3-C4-C5-C6	1.2(12)
C7-C6-C5-C4	0.3(12)	N2-C13-C12-C11	1.9(12)
C9-C10-C11-C12	1.1(12)	C13-C12-C11-C10	-2.3(13)
C19-C18-C17-C16	0.4(15)	C15-C16-C17-C18	-0.7(15)

Table S7. Anisotropic atomic displacement parameters (Å^2) for compound **2a**. The anisotropic atomic displacement factor exponent takes the form: $-2\pi^2[\, h^2 a^{*2} U_{11} + ... + 2\, h\, k\, a^* b^* U_{12}\,]$.

	U_{11}	U_{22}	U_{33}	U_{23}	U_{13}	U_{12}
Ir01	0.03246(13)	0.04585(15)	0.03162(13)	0.00220(9)	0.00029(9)	0.00564(10)
N3	0.023(2)	0.057(3)	0.024(2)	0.002(2)	0.0030(18)	0.011(2)
N2	0.038(3)	0.048(3)	0.043(3)	0.002(2)	0.003(2)	0.001(2)
N4	0.028(2)	0.051(3)	0.042(3)	-0.002(2)	0.008(2)	0.007(2)
N1	0.037(3)	0.056(3)	0.027(2)	0.005(2)	-0.004(2)	0.010(2)
C25	0.037(3)	0.049(4)	0.035(3)	0.007(3)	-0.007(2)	0.003(3)
C14	0.031(3)	0.046(3)	0.041(3)	0.004(3)	-0.006(2)	-0.004(3)
C7	0.038(3)	0.065(4)	0.045(4)	-0.008(3)	0.000(3)	0.004(3)
C8	0.028(3)	0.062(4)	0.042(3)	-0.002(3)	0.007(3)	0.010(3)
C20	0.027(3)	0.066(4)	0.032(3)	0.005(3)	0.001(2)	0.004(3)

	U_{11}	U_{22}	U_{33}	U_{23}	U_{13}	U_{12}
C24	0.043(3)	0.051(4)	0.033(3)	0.004(3)	0.005(3)	0.013(3)
C1	0.056(4)	0.066(5)	0.043(4)	0.017(3)	0.003(3)	0.008(3)
C3	0.033(3)	0.079(5)	0.036(3)	-0.005(3)	0.002(3)	0.008(3)
C23	0.053(4)	0.063(4)	0.043(3)	0.000(3)	-0.004(3)	0.018(4)
C30	0.033(3)	0.050(4)	0.050(4)	0.006(3)	-0.009(3)	0.005(3)
C19	0.029(3)	0.063(4)	0.051(4)	0.007(3)	-0.005(3)	-0.006(3)
C21	0.040(4)	0.076(5)	0.049(4)	0.009(3)	0.006(3)	0.003(3)
C26	0.049(4)	0.063(4)	0.052(4)	0.007(3)	0.000(3)	0.014(3)
C9	0.043(3)	0.051(4)	0.042(3)	0.006(3)	0.011(3)	-0.001(3)
C35	0.041(4)	0.062(4)	0.049(4)	-0.004(3)	0.009(3)	0.003(3)
C31	0.028(3)	0.052(4)	0.058(4)	0.003(3)	0.002(3)	0.005(3)
C22	0.039(4)	0.097(6)	0.039(4)	0.002(4)	0.009(3)	0.017(4)
C34	0.049(4)	0.076(5)	0.064(5)	-0.011(4)	0.020(4)	0.011(4)
C2	0.041(4)	0.088(5)	0.035(3)	0.007(3)	0.004(3)	0.020(4)
C29	0.050(4)	0.075(5)	0.066(5)	0.006(4)	-0.016(4)	0.015(4)
C15	0.048(4)	0.050(4)	0.062(4)	0.007(3)	-0.005(3)	0.009(3)
C27	0.083(5)	0.073(5)	0.034(3)	0.002(3)	-0.009(3)	0.011(4)
B1	0.040(4)	0.056(5)	0.039(4)	0.008(3)	0.009(3)	0.008(3)
C6	0.049(4)	0.079(5)	0.066(5)	-0.020(4)	-0.004(4)	0.005(4)
C33	0.047(4)	0.086(6)	0.089(6)	-0.010(5)	0.010(4)	0.030(4)
C13	0.058(4)	0.056(4)	0.056(4)	-0.001(3)	-0.003(3)	-0.008(3)
C16	0.083(6)	0.048(4)	0.095(6)	0.010(4)	-0.014(5)	-0.001(4)
C28	0.066(5)	0.083(5)	0.052(4)	0.001(4)	-0.026(4)	0.011(4)
C10	0.069(5)	0.058(4)	0.055(4)	0.009(3)	0.007(4)	-0.003(4)
C4	0.050(4)	0.103(6)	0.045(4)	-0.007(4)	-0.007(3)	0.009(4)
C32	0.046(4)	0.082(5)	0.068(5)	-0.001(4)	0.001(4)	0.026(4)
C18	0.047(4)	0.068(5)	0.105(7)	0.012(5)	0.012(4)	-0.010(4)
C5	0.054(4)	0.101(7)	0.059(5)	-0.024(5)	-0.009(4)	-0.004(4)
C12	0.074(5)	0.074(6)	0.076(5)	-0.009(5)	-0.001(5)	-0.021(4)
C11	0.093(6)	0.065(5)	0.077(6)	-0.002(4)	0.009(5)	-0.021(5)
C36	0.082(6)	0.107(7)	0.044(4)	0.007(4)	-0.007(4)	0.032(5)
C17	0.072(6)	0.060(5)	0.127(8)	0.019(5)	0.011(6)	-0.017(4)

Table S8. Hydrogen atomic coordinates and isotropic atomic displacement parameters (Å^2) for **2a**.

	x/a	y/b	z/c	U(eq)
H7	0.6179	0.2859	0.5680	0.059
H24	0.6090	0.4490	0.8183	0.051
H1	0.6326	0.4764	0.4019	0.066
H23	0.5327	0.4753	0.8947	0.064
H21	0.4900	0.3425	0.9396	0.066
H26	0.6464	0.3700	1.0677	0.066
H35	0.7151	0.3196	0.5171	0.061
H22	0.4729	0.4205	0.9550	0.07
H34	0.7887	0.2812	0.4623	0.076
H29	0.8074	0.3088	1.0309	0.076
H15	0.6744	0.2439	0.7510	0.064
H27	0.6962	0.3619	1.2486	0.076
H6	0.5797	0.2403	0.4168	0.077
H33	0.8433	0.2575	0.6220	0.089
H13	0.7427	0.4231	0.8917	0.068
H16	0.6242	0.1805	0.8042	0.091
H28	0.7777	0.3332	1.2301	0.08
H10	0.6879	0.5130	0.5555	0.073
H4	0.5552	0.3517	0.1965	0.079
H32	0.8269	0.2781	0.8344	0.079
H18	0.5151	0.2664	0.9168	0.088
H5	0.5481	0.2739	0.2301	0.085
H12	0.7746	0.4980	0.8690	0.09
H11	0.7440	0.5446	0.7004	0.094
H36A	0.5815	0.4734	0.2151	0.116
H36B	0.5413	0.4331	0.2107	0.116
H36C	0.5929	0.4270	0.1384	0.116
H17	0.5439	0.1924	0.8856	0.104

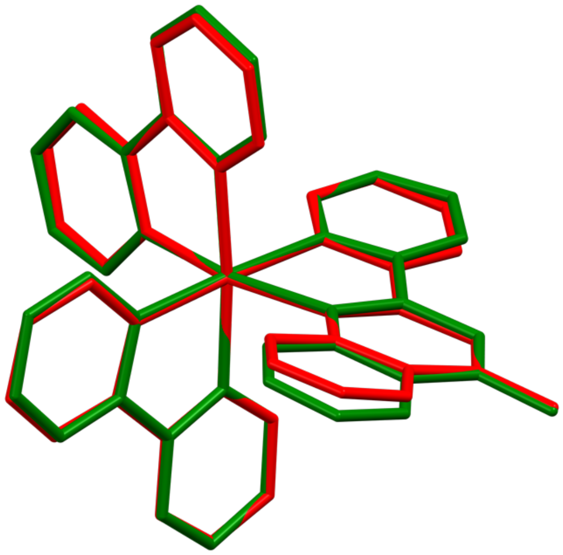

Figure S18. Structural overlay (H atoms omitted) between the experimental X-ray structure of complex **2a** (red) and the DFT-computed one (green). The structural overlay is calculated by minimizing the root-mean-square deviation (RMSD) of all the atomic positions. The low value of RMSD (*i.e.*, 0.159 Å) demonstrates the effectiveness of the adopted theoretical approach.

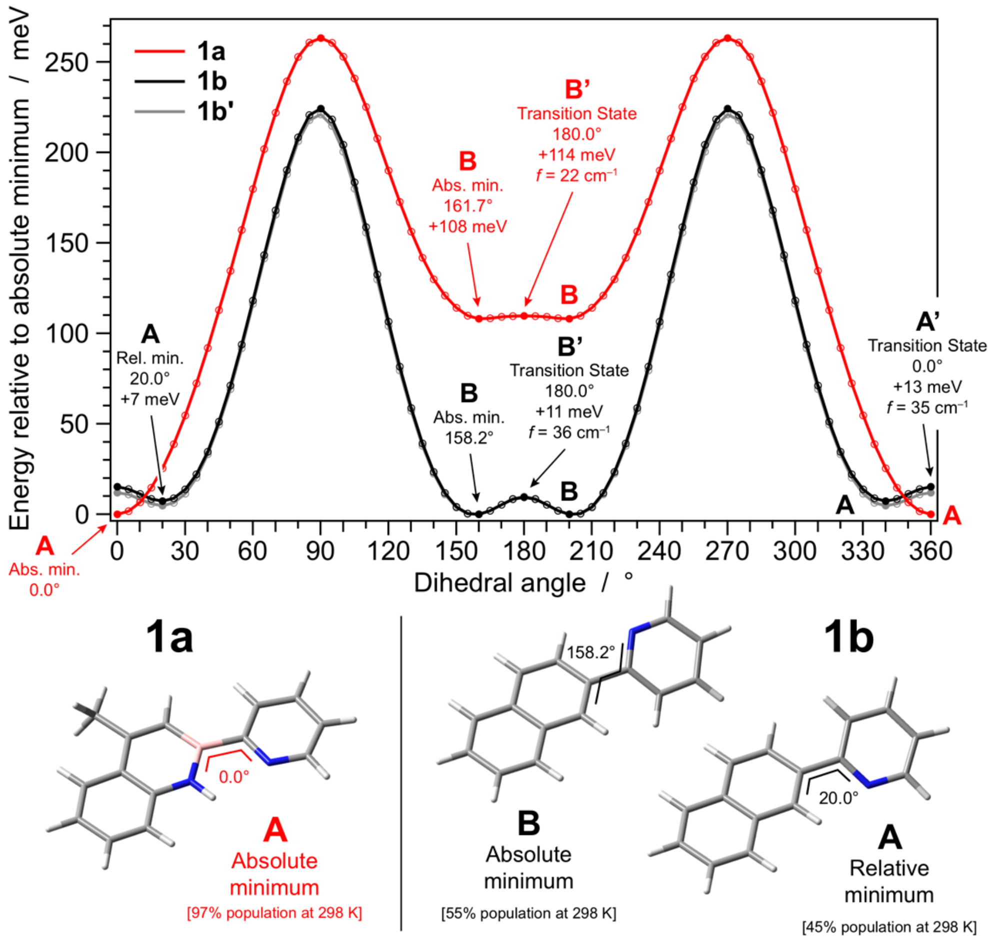

Figure S19. Conformational analysis carried out on the azaborine ligand **1a** and on the C=C counterpart **1b**. The methylated analogue of **1b** (*i.e.*, **1b'**), which would be the exact C=C equivalent of **1a** was also investigated; anyway, the presence of the methyl substituent does not alter the conformational profile of the ligands. A relaxed scan of the dihedral angle between the two aromatic rings is performed in acetonitrile (top), and the most physically important fully-optimized minima are reported together with their Boltzmann population at 298 K (bottom). All energies are reported relative to the absolute minimum of each ligand; two sets of conformers (**A** and **B**) were identified for all molecules; selected transition states (**A'** and **B'**) were also characterized. Since all molecules potentially display reflection symmetry, all scan profiles have π periodicity (*i.e.*, two periods are reported in the top graphs).

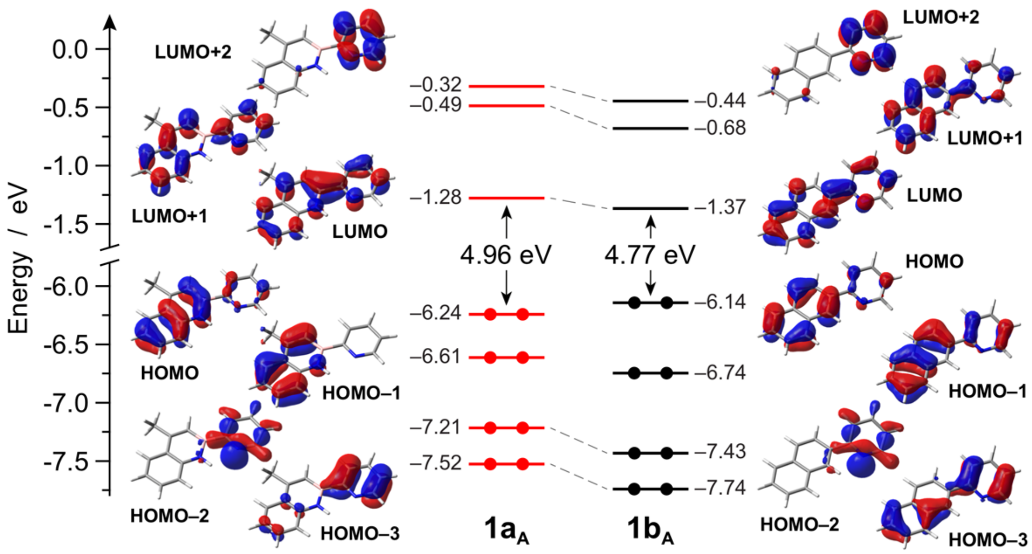

Figure S20. Energy diagram showing the frontier Kohn–Sham molecular orbitals of the azaborine ligand **1a** and of the C=C **1b** in acetonitrile. For both ligands only the **A**-type conformer is reported for a simpler comparison. For all orbitals, the corresponding isosurface is also displayed (isovalue = 0.04 $e^{1/2}$ $bohr^{-3/2}$); orbitals with similar topology are connected by dashed lines.

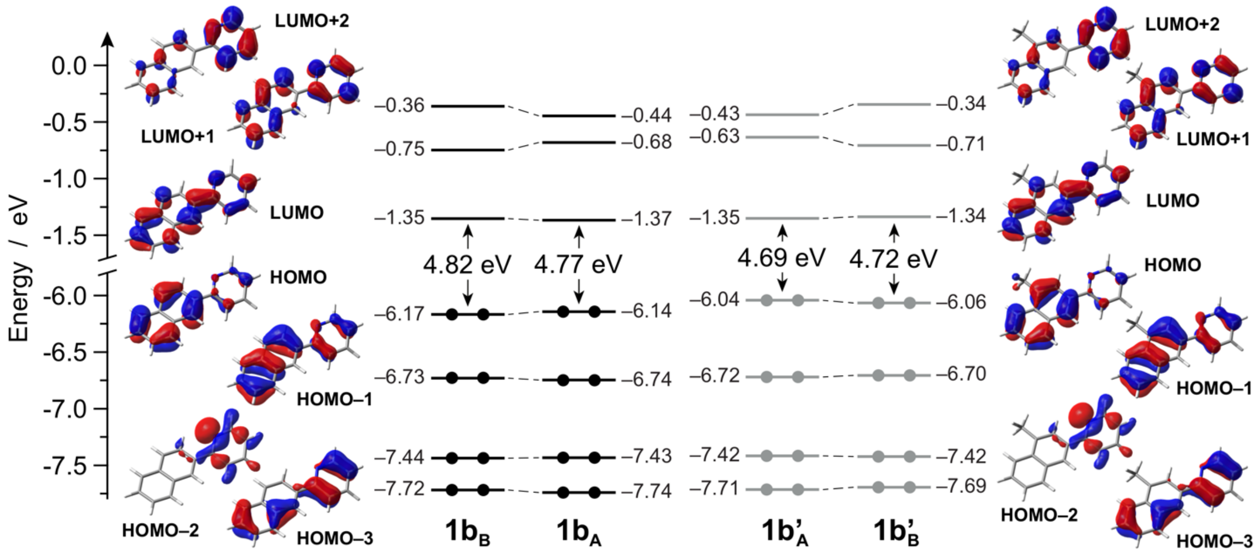

Figure S21. Energy diagram showing the frontier Kohn–Sham molecular orbitals of ligand **1b** and its methylated analogue **1b'** (which would be the exact C=C equivalent of **1a**) in acetonitrile; for both ligands the two nearly isoenergetic conformers (*i.e.,* **A** and **B**) are reported (see Figure S19). No substantial differences are observed between each set of conformers; the effect of the methyl substituent does not significantly alter the energy diagram of **1b'**, compared to **1b**, except for a HOMO destabilization of 0.10 eV. All orbitals are displayed using isosurfaces at 0.04 $e^{1/2}$ $bohr^{-3/2}$; orbitals with similar topology are connected by dashed lines.

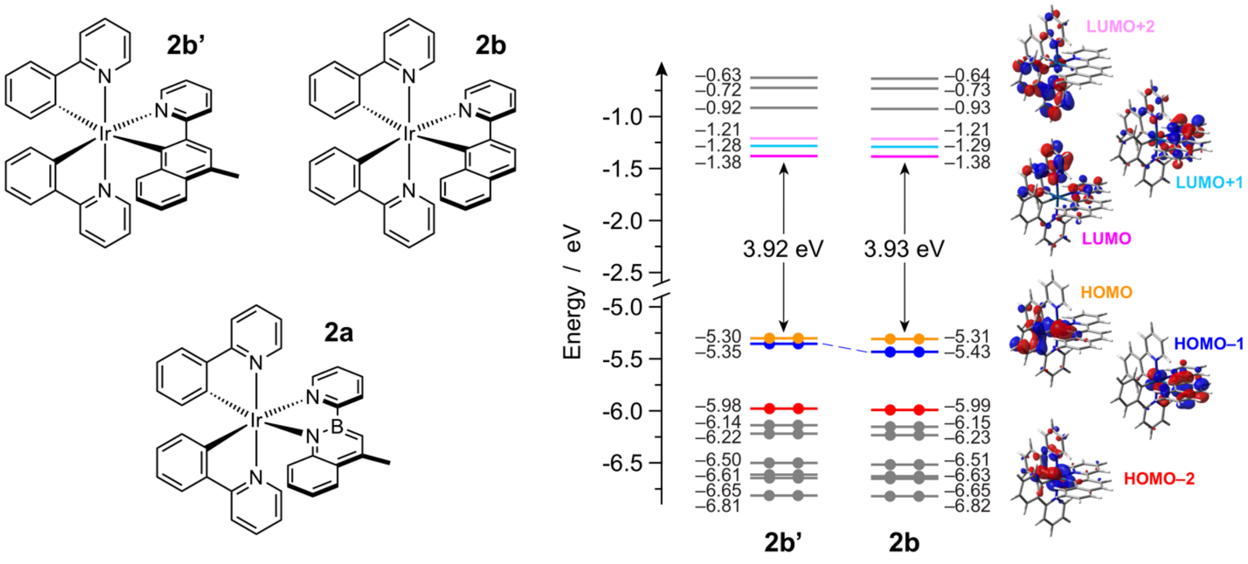

Figure S22. Energy diagram showing the frontier Kohn–Sham molecular orbitals of **2b** together with the one of the methylated analogue **2b'**, which would be the exact C=C equivalent of the azaborine complex **2a**. The presence of the methyl substituent on the ancillary ligand does not alter the energy diagram of **2b'**, if not for a 0.08-eV destabilization of the HOMO–1, which is mainly centered on ancillary ligand itself. Calculations are carried out in acetonitrile, using PCM; relevant orbitals with similar topology are plotted with the same color; orbital-surface isovalue = 0.04 $e^{1/2}$ $bohr^{-3/2}$.

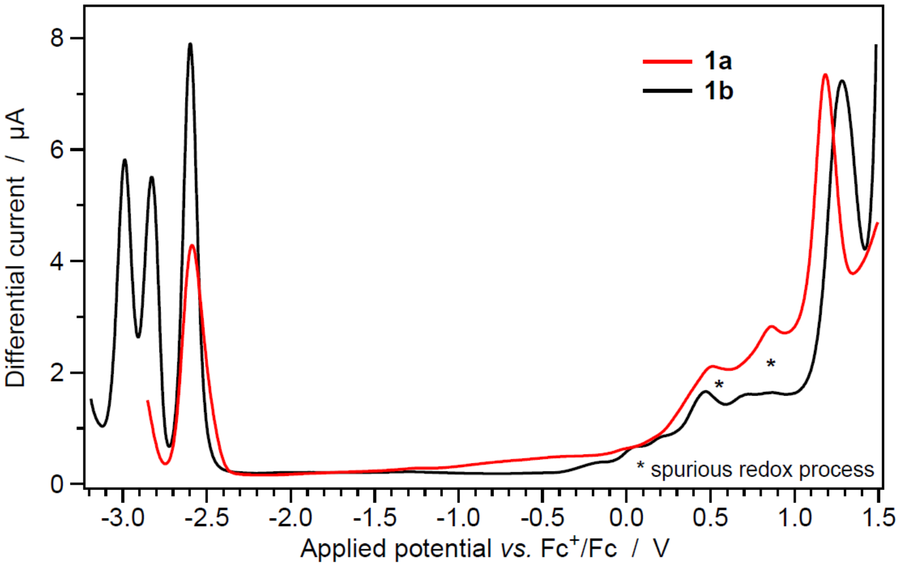

Figure S23. Square-wave voltammograms of ligands **1a** and **1b**. Measurements are made with a glassy carbon electrode in acetonitrile + 0.1 M [*n*Bu_4N][BF_4] at room temperature (frequency 20 Hz, amplitude 20 mV, step 5 mV).

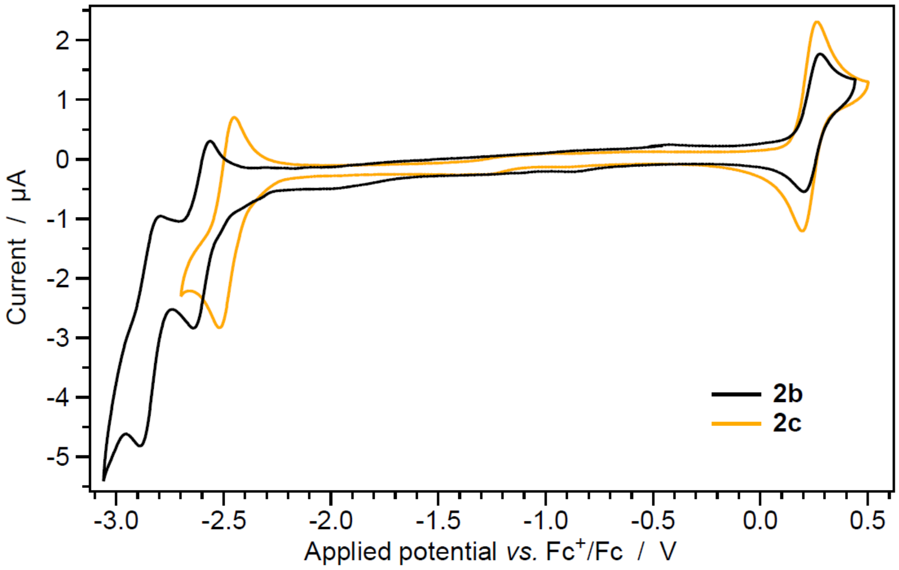

Figure S24. Cyclic voltammograms of complexes **2b** and **2c**. Measurements are made with a glassy carbon electrode in acetonitrile + 0.1 M [nBu$_4$N][BF$_4$] at room temperature.

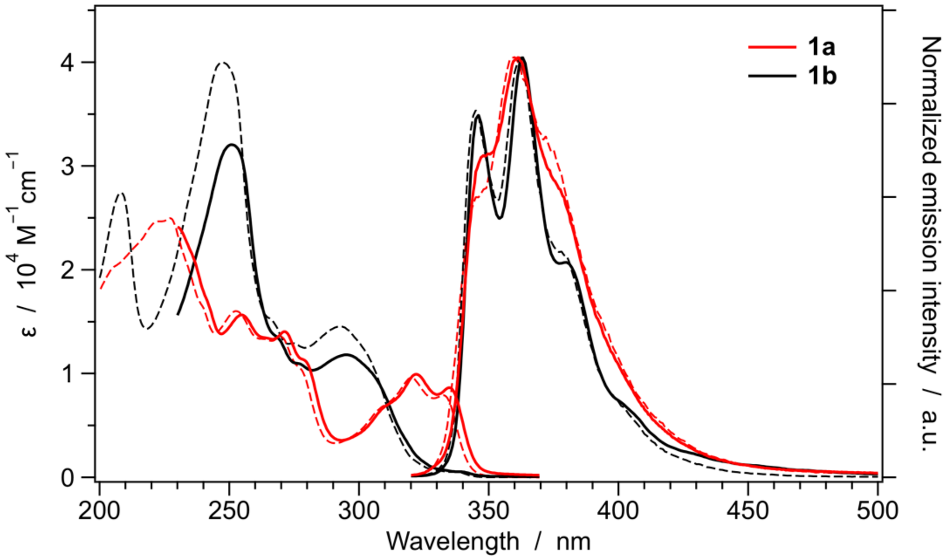

Figure S25. Absorption and fluorescence spectra of **1a** and **1b** in room-temperature dichloromethane solution (thick solid), compared with the ones in acetonitrile solution (thin dashed).

Table S9. NTOs couples describing the lowest singlet excitations (below 5.0 eV) for compounds **1a** and **1b**, calculated at the TD-DFT M06/6-31G(d,p) level in acetonitrile (see Experimental Section for further details). The λ value is the natural transition orbital eigenvalue associated with each NTOs couple; orbital isovalue: 0.04 $e^{-1/2}$ $bohr^{-3/2}$. For **1a** only the predominant conformer **1a$_A$** is considered; for **1b**, both **1b$_A$** and **1b$_B$** conformers were investigated since nearly isoenergetic.

		Transition energy [eV (nm)]	Oscillator strength	NTO couple hole → electron (λ)	Nature
**1a**	$S_0 \rightarrow S_1$	4.09 (303)	0.405	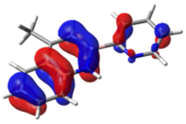 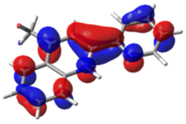 (93.2%)	π–π* partially CT
	$S_0 \rightarrow S_2$	4.39 (282)	0.003	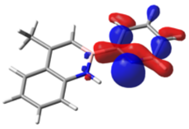 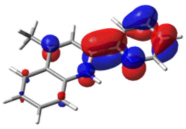 (99.9%)	n–π* pyridine centered
	$S_0 \rightarrow S_3$	4.43 (280)	0.033	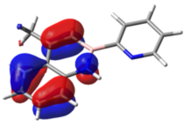 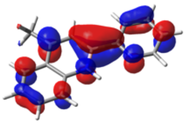 (87.0%)	π–π* partially CT
	$S_0 \rightarrow S_4$	4.82 (257)	0.331	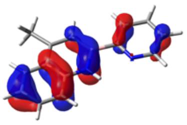 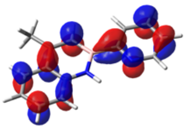 (77.1%)	π–π* partially CT
**1b$_A$**	$S_0 \rightarrow S_1$	3.96 (313)	0.219	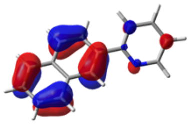 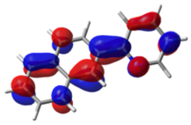 (94.2%)	π–π* partially CT
	$S_0 \rightarrow S_2$	4.17 (298)	0.044	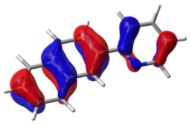 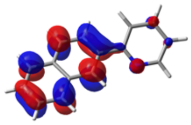 (62.8%)	π–π* mainly centered on naphthyl moiety
	$S_0 \rightarrow S_3$	4.58 (271)	0.002	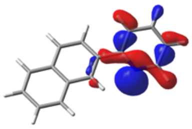 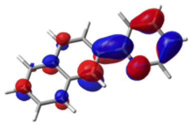 (99.0%)	n–π* pyridine centered
	$S_0 \rightarrow S_4$	4.72 (263)	0.665	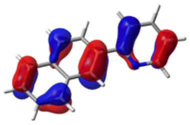 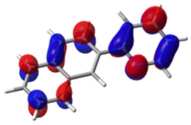 (64.3%)	π–π* partially CT
	$S_0 \rightarrow S_5$	4.85 (256)	0.443	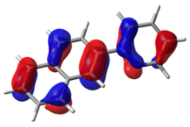 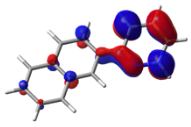 (79.8%)	π–π* predominantly CT

	$S_0 \rightarrow S_1$	3.99 (310)	0.197	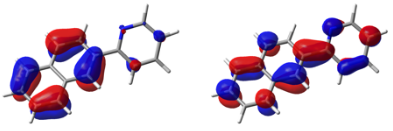 (92.9%)	π–π* partially CT
	$S_0 \rightarrow S_2$	4.18 (297)	0.069	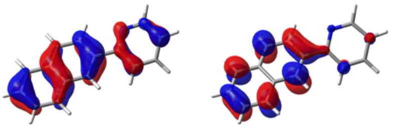 (66.9%)	π–π* mainly centered on naphthyl moiety
**1b$_B$**	$S_0 \rightarrow S_3$	4.62 (268)	0.097	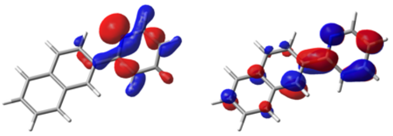 (92.6%)	n–π* pyridine centered
	$S_0 \rightarrow S_4$	4.66 (266)	0.545	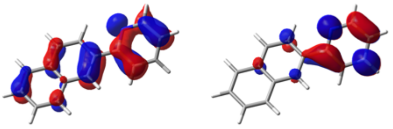 (65.7%)	π–π* predominantly CT
	$S_0 \rightarrow S_5$	4.97 (249)	0.430	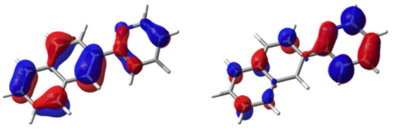 (81.1%)	π–π* partially CT

Table S10. NTOs couples describing the lowest singlet excitations (as in Table S9) for compounds **1a** and **1b**, calculated at the STEOM-DLPNO-CCSD/def2-TZVP level in acetonitrile (see Experimental Section for further details). The λ value is the natural transition orbital eigenvalue associated with each NTOs couple; orbital isovalue: 0.04 $e^{-1/2}$ $bohr^{-3/2}$. For **1a** only the predominant conformer **1a$_A$** is considered; for **1b**, both **1b$_A$** and **1b$_B$** conformers were investigated since nearly isoenergetic.

		Transition energy [eV (nm)]	Oscillator strength	NTO couple hole → electron (λ)	Nature
	$S_0 \rightarrow S_1$	4.01 (310)	0.177	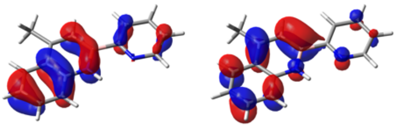 (74.8%)	π–π* minor CT
	$S_0 \rightarrow S_2$	4.52 (274)	0.004	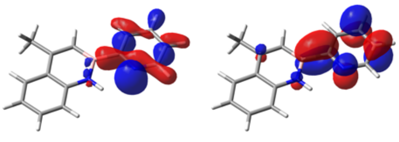 (93.8%)	n–π* pyridine centered
**1a**	$S_0 \rightarrow S_3$	4.68 (265)	0.180	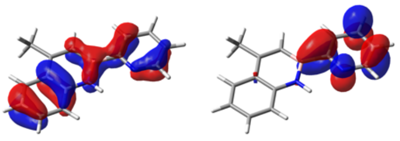 (66.1%)	π–π* partially CT
	$S_0 \rightarrow S_4$	4.88 (254)	0.012	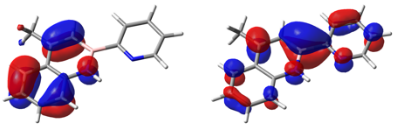 (68.7%)	π–π* partially CT

	$S_0 \rightarrow S_1$	3.90 (318)	0.001	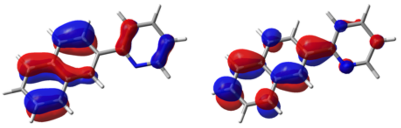 (49.0%)	π–π* mainly centered on naphthyl moiety
	$S_0 \rightarrow S_2$	4.42 (281)	0.316	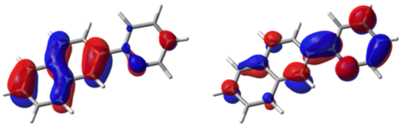 (82.8%)	π–π* partially CT
**1b$_A$**	$S_0 \rightarrow S_3$	4.72 (263)	0.004	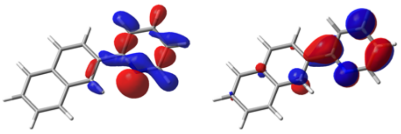 (92.8%)	n–π* pyridine centered
	$S_0 \rightarrow S_4$	4.84 (256)	0.061	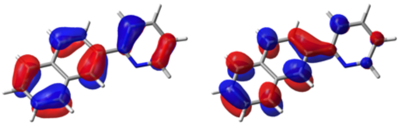 (56.7%)	π–π* partially CT
	$S_0 \rightarrow S_5$	5.34 (232)	1.157	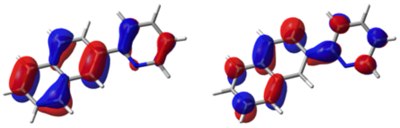 (46.2%) 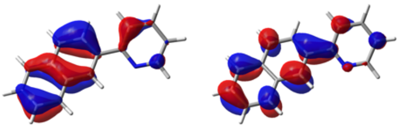 (42.7%)	π–π* partially CT

Table S11. Photophysical data at 77 K for ligands **1a** and **1b** in butyronitrile glass.

	Fluorescence		Phosphorescence	
	λ_{em} [a] [nm]	τ [b] [ns]	λ_{em} [a] [nm]	τ [b] [s]
**1a**	347[sh],357, 374, 391[sh]	2.3	418, 432, 447, 460, 478	2.68
**1b**	344, 361, 380, 400[sh]	12.1	500, 539, 581, 634[sh]	0.90

[a] λ_{exc} = 310 nm. [b] λ_{exc} = 280 nm. [sh] Shoulder.

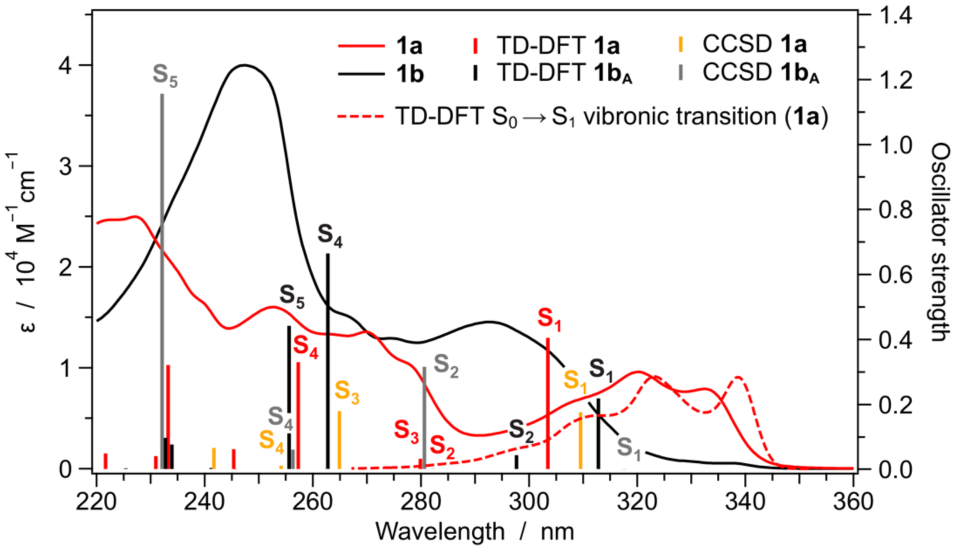

Figure S26. Experimental absorption spectra of **1a** and **1b** in room-temperature acetonitrile solution (full), together with the singlet vertical excitations calculated at the TD-DFT M06/6-31G(d,p) and STEOM-DLPNO-CCSD/def2-TZVP level in acetonitrile (bars). The vibronically resolved $S_0 \rightarrow S_1$ transition for **1a** in acetonitrile is also reported (dashed red line) using the adiabatic hessian approximation and the Franck-Condon and Herzberg-Teller (FCHT) model; each vibronic transition is convoluted with a gaussian function having half-width at half-maximum of 270 cm^{-1}.

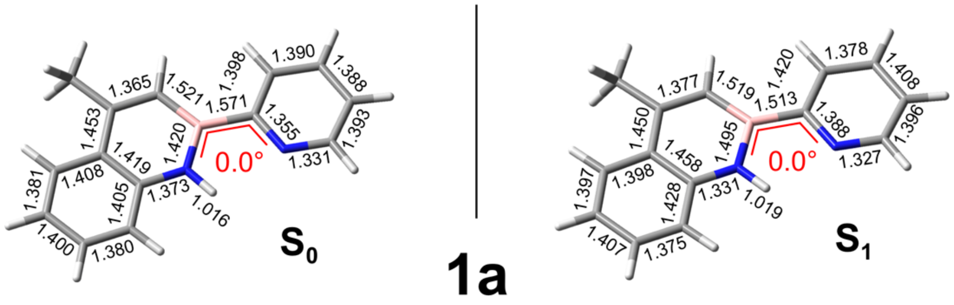

Figure S27. Structural parameters (bond lengths and selected dihedral angles) of **1a** in its S_0 and S_1 minimum-energy geometries. Notably, both structures are very similar and retain planarity.

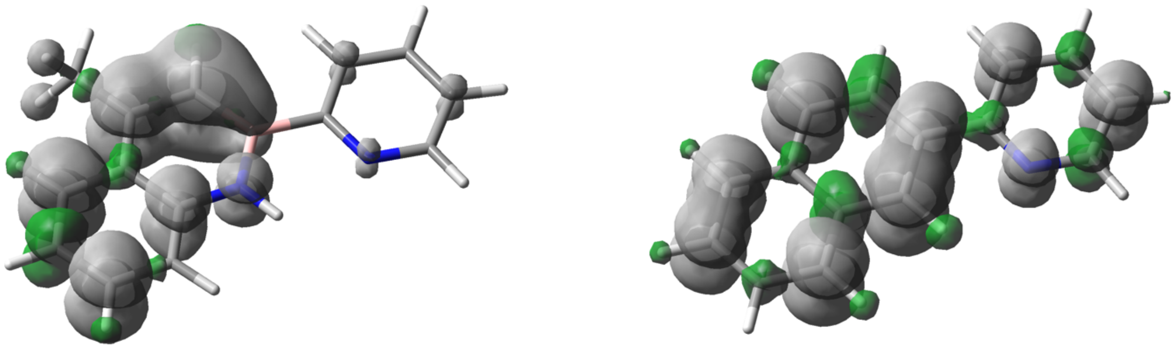

Figure S28. Spin-density distribution of the lowest triplet state of **1a** (left) and **1b** (right) calculated by unrestricted DFT in acetonitrile solution (using PCM). For ligand **1b** only the **A** conformed is depicted for a more direct comparison with the azaborine analogue; anyway, the scenario is identical for conformer $\mathbf{1b_B}$. Spin-density isosurfaces contours are set at 0.002 e bohr^{-3}.

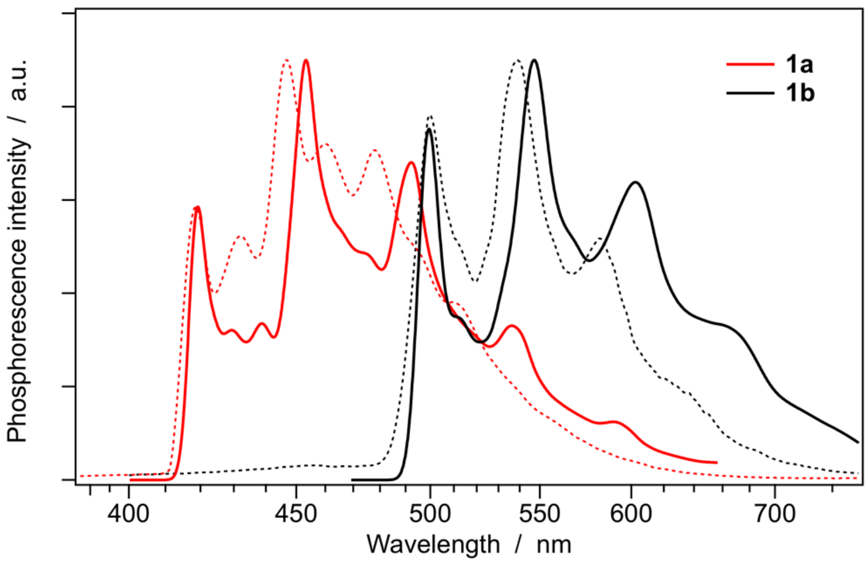

Figure S29. Vibronically-resolved phosphorescence spectra of **1a** and **1b** (thick lines) computed by DFT in acetonitrile solution, using PCM. The vibronic transitions were calculated at 77 K, using the adiabatic-hessian approach and considering up to three simultaneously vibrationally excited modes in the electronic excited state. Simulated spectra are broadened with Gaussian curves with a half-width at half-maximum of 135 cm^{-1} and are blue-shifted by 0.15 eV to allow a better comparison with the experimental spectra (thin dotted lines).

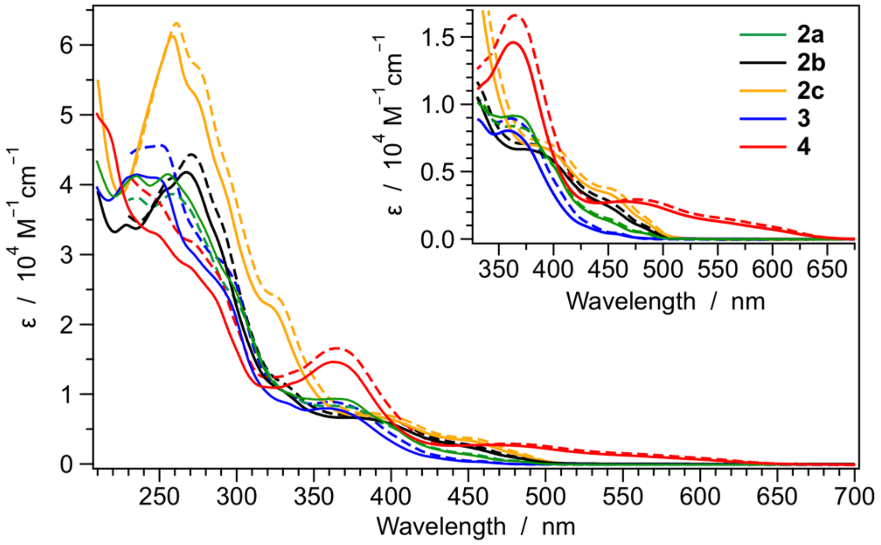

Figure S30. Absorption spectra of the azaborine-based complexes (**2a**, **3** and **4**) and of the isoelectronic C=C analogues (**2b** and **2c**). Spectra are recorded in room-temperature acetonitrile solution (solid) and compared with the correspondents in dichloromethane solution (dashed). Lowest-energy transitions are magnified in the inset. No substantial differences are observed by changing the solvent.

Table S12. Calculated NTOs couples describing the lowest five triplet excitations for complex **2a** in acetonitrile (see Experimental Section for further details). The λ value is the natural transition orbital eigenvalue associated with each NTOs couple; orbital isovalue: 0.04 $e^{-1/2}$ $bohr^{-3/2}$.

	Transition energy [eV (nm)]	NTO couple hole → electron (λ)	Nature
$S_0 \rightarrow T_1$	2.67 (465)	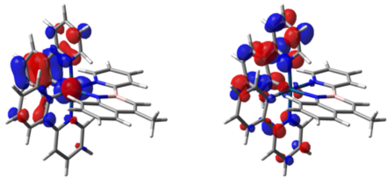 (76.3% + 17.3% mixed with T_2 NTOs)	mainly ^{3}LC on the cyclometalating ligands
$S_0 \rightarrow T_2$	2.69 (460)	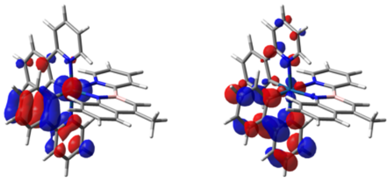 (69.5% + 21.4% mixed with T_1 NTOs)	mainly ^{3}LC on the cyclometalating ligands
$S_0 \rightarrow T_3$	2.70 (459)	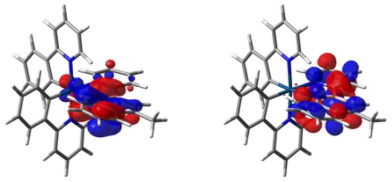 (86.0%)	mainly ^{3}LC on the azaborine ligand
$S_0 \rightarrow T_4$	2.96 (419)	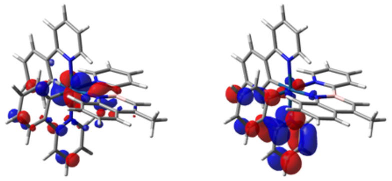 (92.4%)	mainly ^{3}MLCT from the iridium ion to the pyridine of the cyclometalating ligand
$S_0 \rightarrow T_5$	3.07 (404)	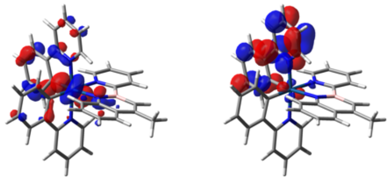 (86.5%)	mainly ^{3}MLCT from the iridium ion to the pyridine of the cyclometalating ligand

Table S13. Calculated NTOs couples describing the lowest six triplet excitations for complex **3** in acetonitrile (see Experimental Section for further details). The λ value is the natural transition orbital eigenvalue associated with each NTOs couple; orbital isovalue: 0.04 $e^{-1/2}$ $bohr^{-3/2}$.

	Transition energy [eV (nm)]	NTO couple hole → electron (λ)	Nature
$S_0 \rightarrow T_1$	2.72 (456)	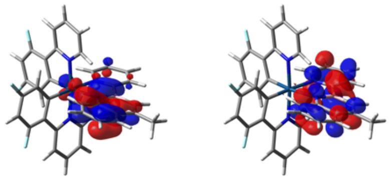 (86.9%)	mainly ^{3}LC on the azaborine ligand
$S_0 \rightarrow T_2$	2.83 (438)	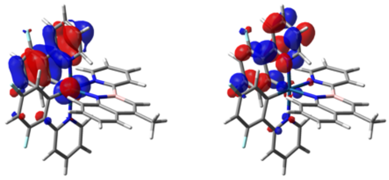 (73.6% + 16.4% mixed with T_3 NTOs)	mainly ^{3}LC on the cyclometalating ligands
$S_0 \rightarrow T_3$	2.85 (436)	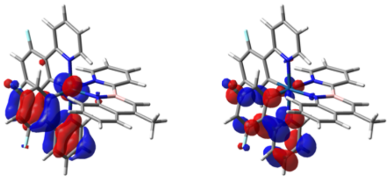 (70.1% + 18.6% mixed with T_2 NTOs)	mainly ^{3}LC on the cyclometalating ligands
$S_0 \rightarrow T_4$	3.09 (401)	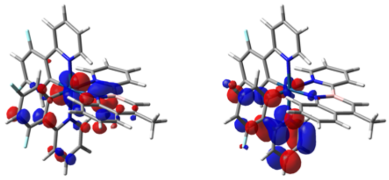 (94.6%)	mainly ^{3}MLCT from the iridium ion to the pyridine of the cyclometalating ligand
$S_0 \rightarrow T_5$	3.15 (394)	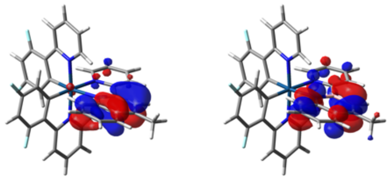 (81.6%)	purely ^{3}LC on the borazaronaphtalene moiety of the azaborine ligand
$S_0 \rightarrow T_6$	3.17 (392)	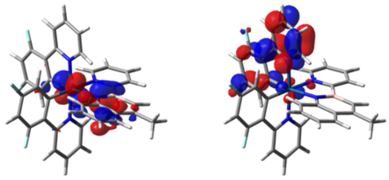 (89.6%)	mainly ^{3}MLCT from the iridium ion to the pyridine of the cyclometalating ligand

Table S14. Calculated NTOs couples describing the lowest six triplet excitations for complex **4** in acetonitrile (see Experimental Section for further details). The λ value is the natural transition orbital eigenvalue associated with each NTOs couple; orbital isovalue: 0.04 $e^{-1/2}$ $bohr^{-3/2}$.

	Transition energy [eV (nm)]	NTO couple hole → electron (λ)	Nature
$S_0 \rightarrow T_1$	1.97 (630)	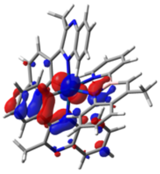 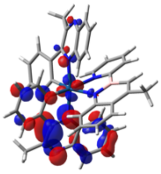 (95.9%)	mainly ^{3}LC on the cyclometalating ligands with minor ^{3}LL'CT contribution
$S_0 \rightarrow T_2$	2.05 (605)	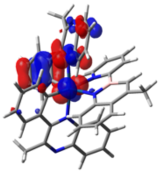 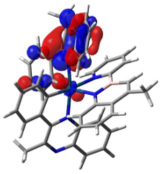 (91.7%)	mainly ^{3}LC on the cyclometalating ligands
$S_0 \rightarrow T_3$	2.09 (594)	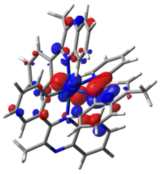 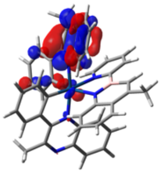 (94.7%)	mainly ^{3}MLCT from the iridium ion to the cyclometalating ligand with minor ^{3}LL'CT contribution
$S_0 \rightarrow T_4$	2.26 (549)	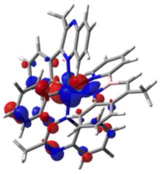 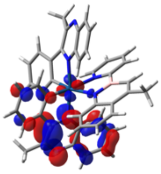 (95.4%)	mainly ^{3}MLCT from the iridium ion to the cyclometalating ligand
$S_0 \rightarrow T_5$	2.56 (483)	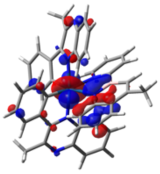 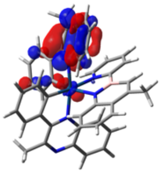 (92.1%)	mixed ^{3}MLCT/^{3}LL'CT involving the iridium ion and both ancillary and cyclometalating ligands
$S_0 \rightarrow T_6$	2.61 (474)	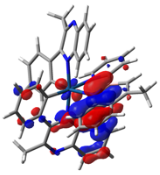 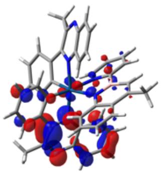 (76.6%)	mainly ^{3}LL'CT from the ancillary ligand to the cyclometalating one

Table S15. Calculated NTOs couples describing the lowest six triplet excitations for complex **2b** in acetonitrile (see Experimental Section for further details). The λ value is the natural transition orbital eigenvalue associated with each NTOs couple; orbital isovalue: 0.04 $e^{-1/2}$ $bohr^{-3/2}$.

	Transition energy [eV (nm)]	NTO couple hole → electron (λ)	Nature
$S_0 \rightarrow T_1$	2.35 (527)	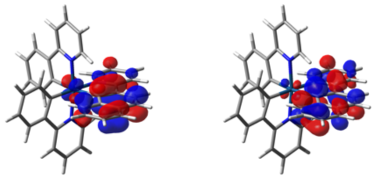 (90.5%)	mainly ^{3}LC on the isoelectronic C=C ancillary ligand
$S_0 \rightarrow T_2$	2.65 (469)	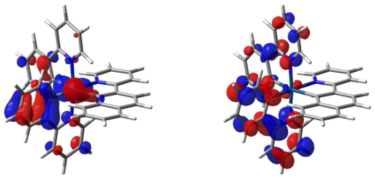 (83.2%)	mainly ^{3}LC on the cyclometalating ligands
$S_0 \rightarrow T_3$	2.69 (461)	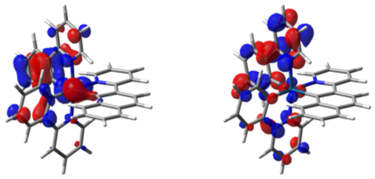 (74.6%)	mainly ^{3}LC on the cyclometalating ligands
$S_0 \rightarrow T_4$	2.87 (432)	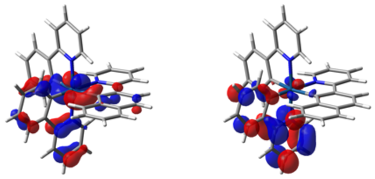 (84.5%)	mainly ^{3}MLCT from the iridium ion to the cyclometalating ligand
$S_0 \rightarrow T_5$	2.92 (425)	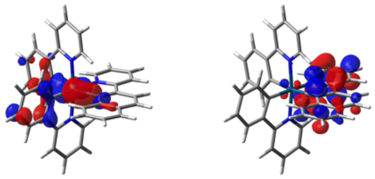 (95.0%)	mainly ^{3}MLCT from the iridium ion to the isoelectronic C=C ancillary ligand
$S_0 \rightarrow T_6$	2.99 (415)	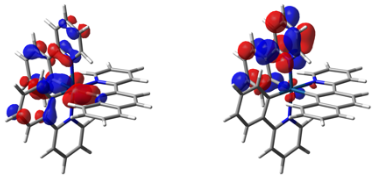 (83.2%)	mainly ^{3}MLCT from the iridium ion to the cyclometalating ligand

Table S16. Calculated NTOs couples describing the lowest six triplet excitations for complex **2c** in acetonitrile (see Experimental Section for further details). The λ value is the natural transition orbital eigenvalue associated with each NTOs couple; orbital isovalue: 0.04 $e^{-1/2}$ $bohr^{-3/2}$.

	Transition energy [eV (nm)]	NTO couple hole → electron (λ)	Nature
$S_0 \rightarrow T_1$	2.31 (537)	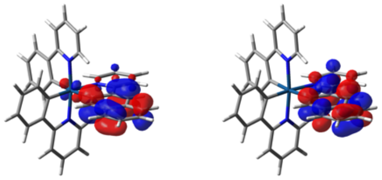 (91.7%)	mainly ^{3}LC on the isoelectronic C=C ancillary ligand
$S_0 \rightarrow T_2$	2.64 (469)	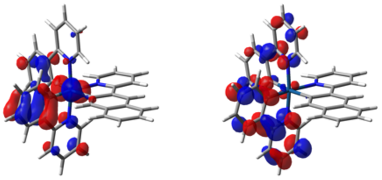 (82.0%)	mainly ^{3}LC on the cyclometalating ligands
$S_0 \rightarrow T_3$	2.69 (461)	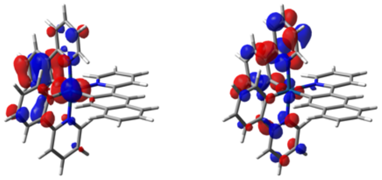 (75.4%)	mainly ^{3}LC on the cyclometalating ligands
$S_0 \rightarrow T_4$	2.82 (439)	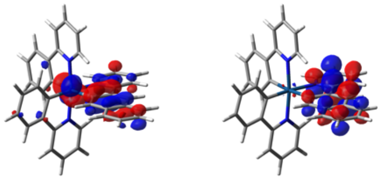 (95.0%)	mixed ^{3}MLCT/^{3}LC involving the iridium ion and isoelectronic C=C ancillary ligand
$S_0 \rightarrow T_5$	2.88 (431)	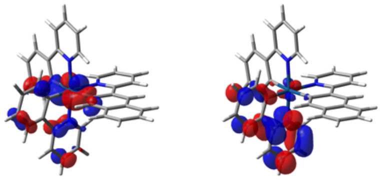 (88.7%)	mainly ^{3}MLCT from the iridium ion to the cyclometalating ligand
$S_0 \rightarrow T_6$	2.93 (422)	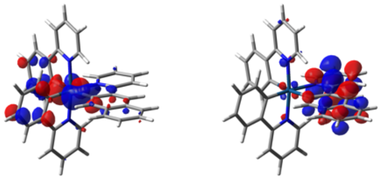 (89.0%)	mainly ^{3}MLCT from the iridium ion to the isoelectronic C=C ancillary ligand

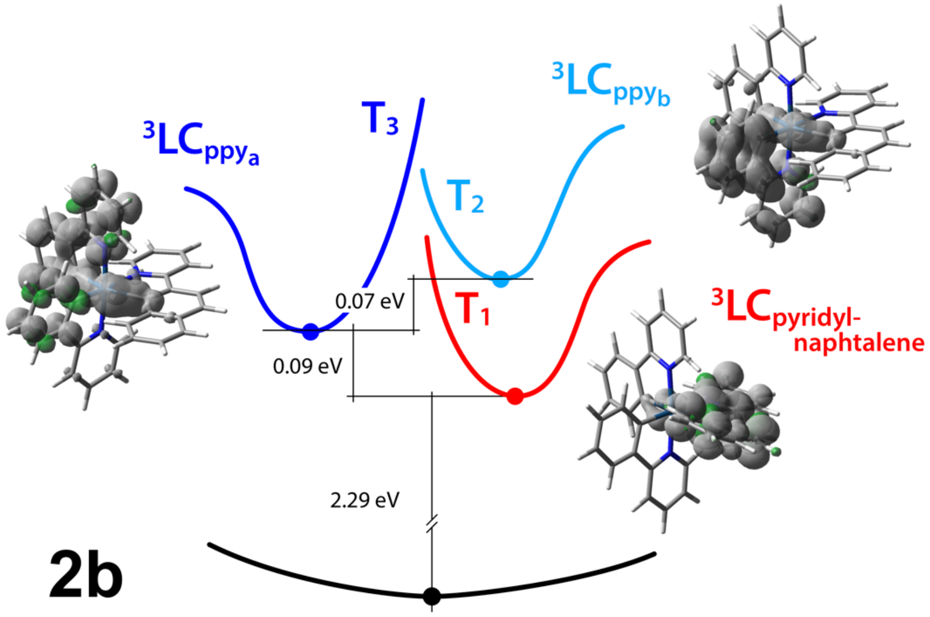

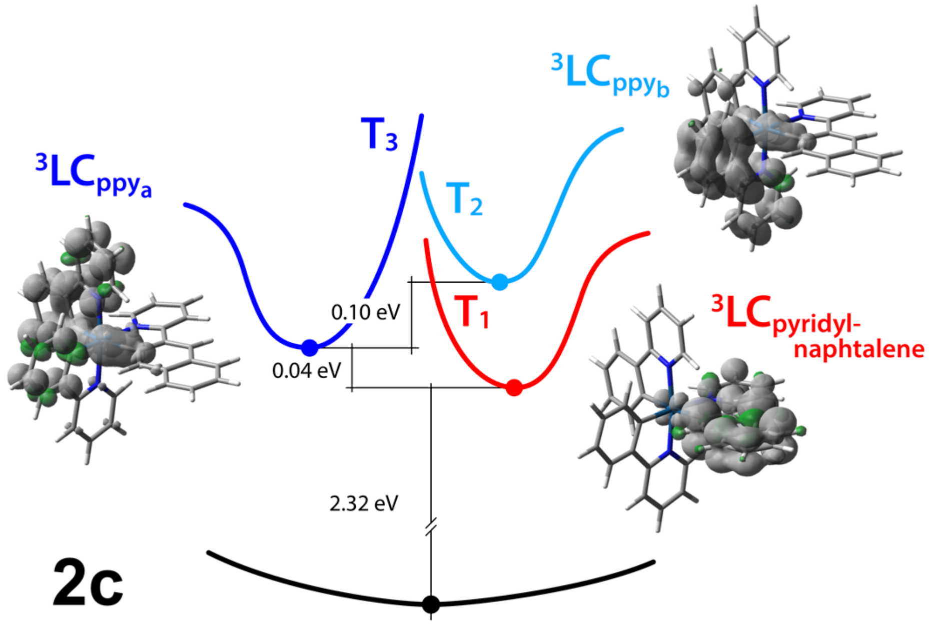

Figure S31. Schematic energy diagram showing the lowest three triplet states of the isoelectronic C=C complexes **2b** and **2c**. Triplet-state numbering follows the order indicated by TD-DFT vertical excitations from S_0 minimum. The unpaired-electron spin-density surfaces calculated at the fully relaxed triplet-state minima are also depicted (isosurfaces: 0.002 e bohr^{-3}).

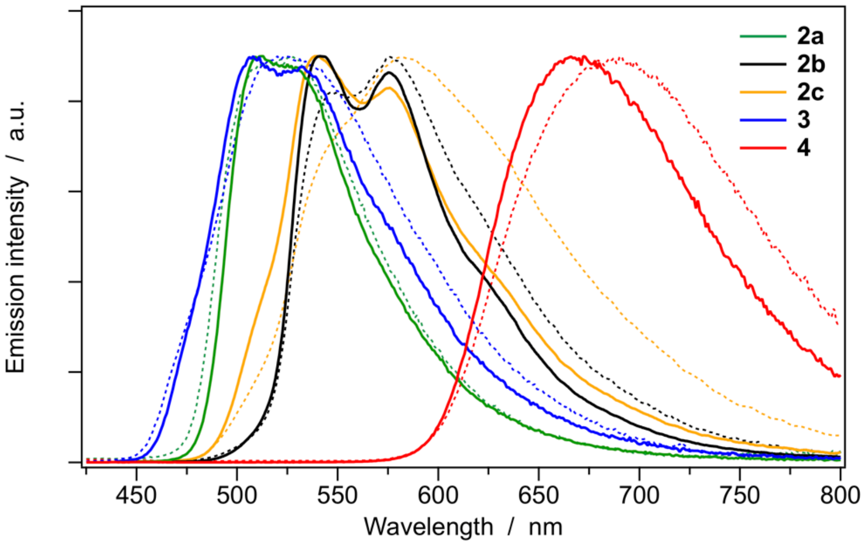

Figure S32. Emission spectra of the complexes with the azaborine ligand (**2a**, **3** and **4**) and with the isoelectronic C=C ligand (**2b** and **2c**) in PMMA matrix at a concentration of 1% by weight. Spectra in acetonitrile are also reported for comparison (dotted).
